# Instrumental Heterogeneity in Sex-Specific Two-Sample Mendelian Randomization: Empirical Results From the Relationship Between Anthropometric Traits and Breast/Prostate Cancer

**DOI:** 10.3389/fgene.2021.651332

**Published:** 2021-06-09

**Authors:** Yixin Gao, Jinhui Zhang, Huashuo Zhao, Fengjun Guan, Ping Zeng

**Affiliations:** ^1^Department of Epidemiology and Biostatistics, School of Public Health, Xuzhou Medical University, Xuzhou, China; ^2^Center for Medical Statistics and Data Analysis, School of Public Health, Xuzhou Medical University, Xuzhou, China; ^3^Department of Pediatrics, Affiliated Hospital of Xuzhou Medical University, Xuzhou, China

**Keywords:** two-sample Mendelian randomization, sex-specific and sex-combined instrumental variable, sex heterogeneity, causal effect estimation, summary statistics, breast cancer, prostate cancer, anthropometric traits

## Abstract

**Background:**

In two-sample Mendelian randomization (MR) studies, sex instrumental heterogeneity is an important problem needed to address carefully, which however is often overlooked and may lead to misleading causal inference.

**Methods:**

We first employed cross-trait linkage disequilibrium score regression (LDSC), Pearson’s correlation analysis, and the Cochran’s *Q* test to examine sex genetic similarity and heterogeneity in instrumental variables (IVs) of exposures. Simulation was further performed to explore the influence of sex instrumental heterogeneity on causal effect estimation in sex-specific two-sample MR analyses. Furthermore, we chose breast/prostate cancer as outcome and four anthropometric traits as exposures as an illustrative example to illustrate the importance of taking sex heterogeneity of instruments into account in MR studies.

**Results:**

The simulation definitively demonstrated that sex-combined IVs can lead to biased causal effect estimates in sex-specific two-sample MR studies. In our real applications, both LDSC and Pearson’s correlation analyses showed high genetic correlation between sex-combined and sex-specific IVs of the four anthropometric traits, while nearly all the correlation coefficients were larger than zero but less than one. The Cochran’s *Q* test also displayed sex heterogeneity for some instruments. When applying sex-specific instruments, significant discrepancies in the magnitude of estimated causal effects were detected for body mass index (BMI) on breast cancer (*P* = 1.63E-6), for hip circumference (HIP) on breast cancer (*P* = 1.25E-20), and for waist circumference (WC) on prostate cancer (*P* = 0.007) compared with those generated with sex-combined instruments.

**Conclusion:**

Our study reveals that the sex instrumental heterogeneity has non-ignorable impact on sex-specific two-sample MR studies and the causal effects of anthropometric traits on breast/prostate cancer would be biased if sex-combined IVs are incorrectly employed.

## Introduction

In the literature of causal inference in observational studies, Mendelian randomization (MR) is a novel statistical method to establish causal relationship between an exposure and an outcome by leveraging genetic variants as instrumental variables (IVs) (Lawlor et al., 2008; Sheehan et al., 2008). To guarantee MR to be valid, each IV needs to satisfy three critical modeling assumptions: (i) strongly associated with the exposure of interest; (ii) not associated with other confounders that are related to both the exposure and the outcome; (iii) influences the outcome only through the pathway of the exposure and does not exhibit any horizontal pleiotropy. The popularity of MR is particularly accelerated by recent successes in large-scale genome-wide association studies (GWASs) (Altshuler et al., 2008; Visscher et al., 2017; McMahon et al., 2019), which make it feasible to choose appropriate single-nucleotide polymorphisms (SNPs) to be eligible instruments for a series of exposures.

However, due to the limitation of data sharing and participant privacy concern, individual-level GWAS datasets are often not accessible; instead, publicly available summary-level statistics are employed in practice, which brings one great benefit that the exposure and the outcome are not required to be measured on the same set of individuals, leading to the so-called two-sample MR study (Lawlor, 2016). Briefly, in the two-sample MR analysis, a genome-wide significant SNP associated with the exposure is first selected as instrument based on which the causal effect is estimated with only marginal effect sizes of the exposure and the outcome. To enhance power, multiple IVs are often leveraged and the individual causal effects can be combined with an inverse-variance weighted (IVW) manner (Burgess et al., 2017). Indeed, the two-sample MR is considerably powerful and flexible and appears technically straightforward to undertake. Due to those reasons, the past few years have witnessed the rapid development and application of MR for causal inference in genetics and epidemiology (Hartwig et al., 2017b; Davies et al., 2018; Zeng and Zhou, 2019a; Yu et al., 2020; Yuan et al., 2020; Liu et al., 2021).

Nevertheless, the two-sample MR still encounters many practical challenges that need to be addressed carefully. For example, the individuals of the two GWASs included in MR studies should take from non-overlapping populations; otherwise, misleading causal effect estimates may be generated (Burgess et al., 2016; Haycock et al., 2016). In addition, because of the difference in SNP effects among diverse populations, the individuals analyzed in the GWASs of exposure and outcome should be of the same ancestry (Zeng and Zhou, 2019b; Zeng et al., 2019). Besides the two issues mentioned above, another important problem is the sex heterogeneity of instruments arising in two-sample MR studies for sex-specific diseases such as breast cancer or prostate cancer. For instance, it is intuitive and natural to employ female-specific (or male-specific) IVs when evaluating the causal association between exposures and breast cancer (or prostate cancer) *via* the two-sample MR. Here, we do not consider male breast cancer, as it only accounts for less than 1% of cases.

However, this seems not to be true in sex-specific two-sample MR studies in terms of our literature review, and we discover that only a few MR studies mentioned in their analyses the problem of sex-specific instruments (Supplementary Tables 1, 2). It is a little surprising that a large amount of sex-specific MR studies exploited sex-combined IVs, which essentially assumed that no sex heterogeneity was present in IVs. However, such an assumption may not hold, since previous GWASs have displayed sex differences in genetic architecture for many exposures including anthropometric traits (Table 1). For example, substantial discrepancies were observed at several adiposity-associated loci, and multiple waist-to-hip ratio (WHR)-associated SNPs showed consistently stronger effects in females compared with males (Heid et al., 2011; Randall et al., 2013; Shungin et al., 2015).

**TABLE 1:** Genetic variants with significant sex difference in effect size for four anthropometric traits.

Traits	Gene	CHR	POS	SNP	Effect		*P*_*Q*_	Folds	References
	
					Female	Male			
BMI	*ZFP64*	20	51,087,862	rs6091540	0.030 (0.004)	0.007 (0.005)	9.05E-05	4.3	Locke et al., 2015
BMI	*SEC16B*	1	177,889,480	rs543874	0.060 (0.005)	0.034 (0.005)	5.23E-05	1.8	Locke et al., 2015
WHR	*LYPLAL1*	1	217,820,132	rs2820443	0.062 (0.005)	0.002 (0.005)	2.60E-17	31.0	Shungin et al., 2015
WHR	*LYPLAL1*	1	217,817,340	rs4846567	0.059 (0.005)	0.005 (0.005)	1.18E-13	11.8	Heid et al., 2011
WHR	*GRB14*	2	165,221,337	rs10195252	0.054 (0.005)	0.010 (0.005)	1.41E-11	5.4	Heid et al., 2011
WHR	*VEGFA*	6	43,872,529	rs1358980	0.060 (0.005)	0.015 (0.005)	3.70E-11	4.0	Shungin et al., 2015
WC	*OR2W5-NLRP3*	1	245,717,763	rs10925060	0.002 (0.005)	0.045 (0.006)	1.70E-08	22.5	Shungin et al., 2015
WC	*CCNJL*	5	159,626,935	rs17472426	−0.014 (0.009)	0.052 (0.010)	3.90E-08	3.7	Shungin et al., 2015
HIP	*KLHL31*	6	53,648,294	rs7739232	0.063 (0.011)	−0.004 (0.014)	2.90E-05	15.8	Shungin et al., 2015
HIP	*C5-FBXW2*	9	122,533,883	rs7044106	0.039 (0.007)	−0.003 (0.008)	1.30E-05	13.0	Shungin et al., 2015
HIP	*KLF14*	7	130,090,402	rs13241538	0.033 (0.005)	−0.003 (0.005)	2.00E-09	11.0	Shungin et al., 2015

*CHR, chromosome; POS, position; SNP, single-nucleotide polymorphism; BMI, body mass index; WHR, waist-to-hip ratio; WC, waist circumference; HIP, hip circumference. Note that the SNP effect of WHR, WC, and HIP was generated under the control of BMI.*

*The value in the parentheses is the standard error.*

**P*_*Q*_ denotes the *P* value of the Cochran’s *Q*-test. Fold is computed based on the absolute effect and is always shown to be larger than 1.*

Although it is particularly important, a formal investigation about the sex instrumental heterogeneity in sex-specific two-sample MR studies is lacking and its consequence seems to be also overlooked by many MR researchers. As a result, improper causal inference might be generated (Lawlor, 2016; Tan et al., 2019). Therefore, the main goal of this work is to explore the influence of sex instrumental heterogeneity on causal effect estimation in two-sample MR analyses when applying sex-specific and sex-combined effects of IVs. In the following, we first described sex genetic similarity and heterogeneity among instruments and demonstrated that the sex instrumental heterogeneity had a non-ignorable impact on causal inference in the sex-specific two-sample MR; this statement was further supported by our numerical simulation. Moreover, as an illustrative example, we chose breast/prostate cancer as the outcome and four anthropometric traits as exposures to explain the possible consequence in real data analysis. We revealed that the causal effects of anthropometric traits on both breast and prostate cancers would be to some extent changed when using sex-specific IVs compared with those generated with sex-combined instruments. We finally offered several valuable suggestions in practical sex-specific two-sample MR studies.

## Materials and Methods

### Genome-Wide Association Study Dataset Sources and Instrument Selection

We initially obtained sex-combined and sex-specific summary statistics of four anthropometric traits [i.e., body mass index (BMI), waist-to-hip ratio (WHR), waist circumference (WC), and hip circumference (HIP)] for individuals of European ancestry from the Genetic Investigation of ANthropometric Traits (GIANT) Consortium (Locke et al., 2015; Shungin et al., 2015). For each SNP in the GIANT study, the association was performed while adjusting for age, age^2^ and study-specific covariates *via* linear regression. In addition, the SNP effect of WHR, WC, or HIP was estimated under the control of BMI. Based on these GWAS datasets, we yielded a set of uncorrelated associated SNPs (*P* < 5.00E-8) to serve as sex-combined or sex-specific IVs for each anthropometric trait (Supplementary Table 3).

We next acquired summary statistics of breast cancer from the Breast Cancer Association Consortium (BCAC) (Michailidou et al., 2017) and summary statistics of prostate cancer from the Prostate Cancer Association Group to Investigate Cancer-Associated Alterations in the Genome (PRACTICAL) consortium (Schumacher et al., 2018). In the GWAS of the two types of cancer, the association was also undertaken with individuals of European descent. The SNP effect size was estimated *via* logistic regression with principal components as covariates and sometimes additionally adjusted for study-specific covariates. The GWAS datasets employed in our study are summarized in Table 2.

**TABLE 2:** Summary information of the GWAS datasets employed in our sex-specific two-sample MR analysis.

Trait	Sample size (case/control)	*k*_0_	*M*	References
**BMI**				
Sex-combined	322,154	97	2,517,828	Locke et al., 2015
Female-specific	171,977	38	2,459,695	Locke et al., 2015
Male-specific	152,893	30	2,443,565	Locke et al., 2015
**WHR**				
Sex-combined	210,086	39	2,542,431	Shungin et al., 2015
Female-specific	116,742	34	2,467,778	Shungin et al., 2015
Male-specific	93,480	3	2,146,136	Shungin et al., 2015
**WC**				
Sex-combined	231,355	70	2,545,772	Shungin et al., 2015
Female-specific	127,470	25	2,473,035	Shungin et al., 2015
Male-specific	104,079	29	2,294,965	Shungin et al., 2015
**HIP**				
Sex-combined	211,117	89	2,540,653	Shungin et al., 2015
Female-specific	117,340	41	2,466,814	Shungin et al., 2015
Male-specific	93,965	31	2,188,855	Shungin et al., 2015
**Breast cancer**	228,951 (122,977/105,974)			
Female-specific			13,011,123	Michailidou et al., 2017
**Prostate cancer**	140,306 (79,194/61,112)			
Male-specific			16,486,833	Schumacher et al., 2018

*BMI, body mass index; WHR, waist-to-hip ratio; WC, waist circumference; HIP, hip circumference; SNP, single-nucleotide polymorphism; GWAS, genome-wide association study; MR, Mendelian randomization.*

*Note that k0 denotes the number of candidate instruments that were obtained directly with those associated SNPs (*P* < 5.00E-8) reported in the original GWAS papers; *M* is the total number of SNPs.*

### Detection of Sex Genetic Similarity and Heterogeneity for Anthropometric Traits

To evaluate genetic similarity between the sex-combined and sex-specific effects of anthropometric traits as well as between the male-specific and female-specific effects of anthropometric traits, we applied cross-trait linkage disequilibrium score regression (LDSC) to calculate overall genetic correlation ρ*_*g*_* with all available SNPs (Bulik-Sullivan et al., 2015). The LD scores were computed with genotypes of 503 European individuals in the 1000 Genomes Project (The 1000 Genomes Project Consortium, 2015) and then regressed on the product of *Z* statistics of two traits. The regression slope provides an unbiased estimate for ρ*_*g*_*. The software of LDSC (version v1.0.1) was downloaded from https://github.com/bulik/ldsc.

We also carried out the Pearson’s correlation analysis to quantify the genetic effect correlation *r*_*g*_ among those independently associated SNPs that served as IVs. Note that, unlike ρ*_*g*_*, which quantifies the global genetic overlap of two traits using genome-wide variants, *r*_*g*_ can be viewed as a measurement of marginal genetic sharing between two traits because of only a small set of associated SNPs involved. We further assessed sex heterogeneity in each IV via the Cochran’s *Q* test. Specifically, the sex heterogeneity was tested based on sex-specific SNP effect estimates and standard errors; the *P* value of heterogeneity was corrected with the Bonferroni’s method to take multiple comparisons into account. Finally, the sex heterogeneity was quantified with the I^2^ statistic that was widely used in the literature.

### Simulation Study to Assess the Influence of Sex Instrumental Heterogeneity

Given the potential sex instrumental heterogeneity in IVs (Heid et al., 2011; Randall et al., 2013; Shungin et al., 2015), we implemented a simple simulation to assess its influence on causal inference in sex-specific two-sample MR studies. To obtain exposures, we first generated *m* uncorrelated genetic variants with *m* following a uniform distribution ranging from 50 to 150. The minor allele frequency (MAF) of these SNPs was independently sampled from a uniform distribution ranging from 0.01 to 0.50. The genotypes (denoted by G_1_ or G_2_) were separately generated for *N*_1_ (1 × 10^5^) male or *N*_2_ (1 × 10^5^) female individuals under the assumption of linkage equilibrium and Hardy–Weinberg equilibrium (HWE). The effect sizes (denoted by α_1_ and α_2_) for the two sets of SNPs were drawn from a bivariate normal distribution with μ = (0, 0) and Σ = ([1, *r*_*g*_], [*r*_*g*_, 1]), with *r*_*g*_ varying from 0.1, 0.3, 0.5, to 0.7. Note that *r*_*g*_ also partly quantified the genetic effect heterogeneity of the exposure between females and males for these SNPs; that is, smaller *r*_*g*_ indicated larger heterogeneity. The residual error terms (denoted by *e*_1_ or *e*_2_) were separately sampled from independent standard normal distributions. We further rescaled α_1_ and α_2_ so that the phenotypic variance explained (PVE) by SNPs can be set to the given value for the male exposure and the female exposure.

(1) PVEl=δl2×∑j=1mαl⁢j2δl2×∑j=1mαl⁢j2+1,l=1⁢or⁢ 2

where δ is the scale parameter and can be estimated (denoted by $\hat{\delta }$) in terms of (1). Note that *r*_*g*_ would not be impacted by the scaled effect sizes. After doing those, the exposures (denoted by *x*_1_ and *x*_2_) can be obtained.

(2) $$x_{1\,i}={\hat{\delta }}_{1}\times \sum_{j=1}^{K}G_{1\,i\,j}\,\alpha_{1\,j}+e_{1\,i},x_{2\,i}={\hat{\delta }}_{2}\times \sum_{j=1}^{K}G_{2\,i\,j}\,\alpha_{2\,j}+e_{2\,i},i=1,\cdots ,N_{1}\,\text{or}\,N_{2}$$

Then, a *female-specific* outcome was created as *y* = x_2_ × θ + ε based on the same set of individuals, where θ was the true causal effect size varying from 0.1, 0.3, to 0.5 and ε was the residual error term following a standard normal distribution. The single SNP association analysis was conducted to obtain summary statistics for each genetic variant (Zeng et al., 2015). Specifically, the sex-specific summary statistics of the exposure were yielded by regressing *x*_1_ on G_1_ (or *x*_2_ on G_2_), while the sex-combined summary statistics of the exposure were yielded with the fixed-effects IVW meta-analysis based on the two sets of sex-specific summary statistics. The SNPs with marginal *P* values less than 0.05/*m* were identified to be female-specific or sex-combined IVs. If no SNPs satisfied this criterion, the one with the minimum *P* value would be employed. With the selected IVs, the female-specific and sex-combined causal effects of the exposure on the outcome were evaluated with the fixed-effects IVW MR approach (Burgess et al., 2017; Hartwig et al., 2017a; Yavorska and Burgess, 2017).

(3) $$\hat{\theta }=\frac{1}{\sum_{i=1}^{k}\text{var}\,{\left({\hat{a}}_{i}^{y}\right)}^{-1}\,{\left({\hat{a}}_{i}^{x}\right)}^{2}}\,\sum_{i=1}^{k}\text{var}\,{\left({\hat{a}}_{i}^{y}\right)}^{-1}\,{\hat{a}}_{i}^{y}\,{\hat{a}}_{i}^{x}$$

where $\hat{a}$ denotes the marginal effect size of the selected IV, $\hat{a}$ denotes the corresponding variance of the estimated effect size, and *k* is the number of the used IVs. Note that $\hat{\theta }={\hat{a}}^{y}/{\hat{a}}^{x}$ when only one IV was employed. We can approximately estimate the asymptotic variance of $\hat{\theta }$ by:

(4) $$\text{var}\,\left(\hat{\theta }\right)=\frac{1}{\sum_{i=1}^{k}\text{var}\,{\left({\hat{\alpha }}_{i}^{y}\right)}^{-1}\,{\left({\hat{\alpha }}_{i}^{x}\right)}^{2}}$$

### Estimation of Causal Effect With Two-Sample Mendelian Randomization

Before the formal MR analysis, we performed several stringent quality control procedures for IVs: (i) excluded SNPs that were not included in the breast/prostate cancer GWASs; (ii) excluded SNPs whose alleles were inconsistent between the exposure and the outcome; (iii) excluded SNPs that were likely related to breast/prostate cancer if the Bonferroni-corrected *P* values < 0.05. Note that this is a conservative way of protecting against the pleiotropic impact of instruments to ensure valid causal inference in MR studies (Zeng and Zhou, 2019a; Zeng et al., 2019). After the quality control, we performed the two-sample IVW MR to estimate the causal effect of each anthropometric trait on breast/prostate cancer with sex-combined or sex-specific IVs. The causal relationship was mainly illustrated in terms of odds ratio (OR) per standard deviation (SD) increase in anthropometric trait because all the anthropometric traits were previously standardized.

For sex-specific IVs, once a significant causal relationship was identified between one of the anthropometric traits and breast/prostate cancer *via* the IVW approach, we further implemented the weighted median method (Bowden et al., 2016b), the maximum likelihood method (Burgess et al., 2013), the leave-one-out (LOO) analysis (Noyce et al., 2017), the MR-PRESSO test (Verbanck et al., 2018), and the MR-Egger regression (Bowden et al., 2016a; Burgess and Thompson, 2017) as part of sensitivity analyses to examine the robustness of our results.

Finally, we formally compared the causal effects generated with sex-combined or sex-specific instruments (*H*_0_: θ_*combined*_ = θ_*specific*_) by a simple *u* test:

(5) $$u=\frac{{\hat{\theta }}_{\text{combined}}-{\hat{\theta }}_{\text{specific}}}{\sqrt{\begin{array}{c} {\left\{s\,e\,\left({\hat{\theta }}_{\text{combined}}\right)\right\}}^{2}+{\left\{s\,e\,\left({\hat{\theta }}_{\text{specific}}\right)\right\}}^{2}\\ -2\,\rho \times s\,e\,\left({\hat{\theta }}_{\text{combined}}\right)\times s\,e\,\left({\hat{\theta }}_{\text{specific}}\right) \end{array}}}$$

where $\hat{\theta }$ is the estimated causal effect with the standard error $s\,e\,\left(\hat{\theta }\right)$, and ρ is the correlation coefficient between the two estimated causal effects that are expected to be highly correlated. However, it is rather challenging to estimate ρ accurately with only summary statistics. In the present study, we calculated ρ with the LOO jackknife method for each anthropometric trait (Efron, 1982; Efron and Tibshirani, 1993). Specifically, for all the used sex-combined and sex-specific IVs (Supplementary Table 3), we removed one at a time and recomputed the causal effect on breast/prostate cancer using the rest of sex-combined and sex-specific instruments, respectively. We then calculated ρ using the sex-combined causal effects and the sex-specific causal effects. The *P* value of the *u* statistic can be easily calculated as it asymptotically follows a standard normal distribution.

## Results

### Sex Heterogeneity of Instrumental Variables

**FIGURE 1:**
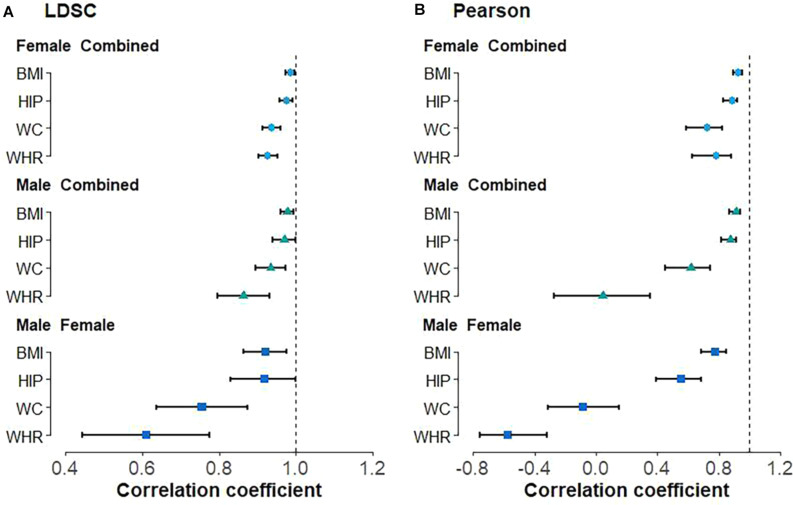
Genetic correlation between sex-combined and sex-specific anthropometric traits as well as between male-specific and female-specific anthropometric traits using **(A)** linkage disequilibrium score regression (LDSC) and **(B)** Pearson’s correlation analyses. BMI, body mass index; WHR, waist-to-hip ratio; WC, waist circumference; HIP, hip circumference.

With LDSC, we observe high positive genetic correlation between male and female anthropometric traits (Figure 1A), with ${\hat{\rho }}_{g}$ = 0.920 (se = 0.028) for BMI, ${\hat{\rho }}_{g}$ = 0.919 (se = 0.046) for HIP, ${\hat{\rho }}_{g}$ = 0.755 (se = 0.060) for WC, and ${\hat{\rho }}_{g}$ = 0.609 (se = 0.085) for WHR. Similar positive genetic correlations were also detected between the sex-combined and sex-specific anthropometric traits, with all ${\hat{\rho }}_{g}$ larger than 0.85 (Figure 1A). Nearly all these estimated genetic correlations are substantially larger than 0 (*H*_01_: ρ*_*g*_* = 0) but less than 1 (*H*_02_: ρ*_*g*_* = 1) (Figure 1A); the only exception is the genetic correlation of HIP between males and females, which is marginally significant (${\hat{\rho }}_{g}$ = 0.919 and *P* = 0.076 for *H*_02_). In addition, the Pearson’s correlation analysis also showed that all the correlations between the sex-combined and sex-specific instruments are less than 1 (*H*_30_: *r*_*g*_ = 1) (Figure 1B, Supplementary Figure 1). It needs to note that two negative Pearson’s correlations even occur, i.e., ${\hat{r}}_{g}$ = −0.087 (*P* = 0.472) for WC and ${\hat{r}}_{g}$ = −0.577 (*P* = 1.69E-5) for WHR between males and females (Supplementary Figures 1C3, D3), indicating that the marginal genetic effect correlation can have a fully opposite direction compared with its global counterpart.

Using the Cochran’s *Q* test, we detect two out of 97 (2.1%) BMI-associated SNPs, four of 95 (4.2%) HIP-associated SNPs, 10 of 76 (13.2%) WC-associated SNPs, and 17 of 48 (35.4%) WHR-associated SNPs exhibit sex heterogeneity among candidate IVs (Supplementary Table 3). In particular, all WC-associated SNPs but one (i.e., rs17472426 with β_*male*_ = 0.052 and β_*female*_ = −0.014, *P*_*het*_ = 9.31E-07) and all WHR-associated SNPs but three (i.e., rs224333 with β_*male*_ = 0.036 and β_*female*_ = 0.009, *P*_*het*_ = 1.34E-4; rs10925060 with β_*male*_ = 0.045 and β_*female*_ = 0.002, *P*_*het*_ = 3.68E-8; rs3791679 with β_*male*_ = 0.053 and β_*female*_ = 0.021, *P*_*het*_ = 4.18E-5) are found to have larger effect sizes on females compared with males (Supplementary Table 3). These findings, together with the estimates of genetic correlation described above, indicate the existence of potential sex genetic effect heterogeneity in the four anthropometric traits, although they indeed share widely common genetic components.

### Influence of Sex Instrumental Heterogeneity in Terms of the Simulation

**FIGURE 2:**
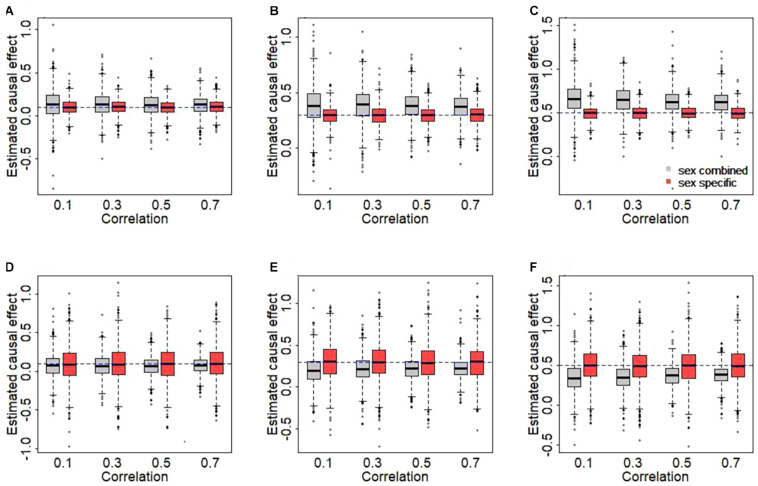
Estimated causal effects with sex-combined or sex-specific instrumental variables in the simulation. **(A,D)** The true causal effect is 0.1. **(B,E)** The true causal effect is 0.3. **(C,F)** The true causal effect is 0.5. In the top panel, the phenotypic variance explained (PVEs) of the male and the female exposures are 1 and 3%, respectively. In the bottom panel, the PVEs of the male and the female exposures are 3 and 1%, respectively.

The results of simulation are displayed in Figure 2. We here clearly find that the use of sex-combined instruments can lead to biased causal effect estimates in our simulated case of *female-specific* two-sample MR. Specifically, the female-specific causal effect is overestimated if the PVE of the male exposure is smaller than that of the female exposure (1% vs. 3%) (Figures 2A–C). The main reason is that under this situation, the average of the sex-combined effect sizes of the IVs tends to be smaller compared to the average of female-specific effects of the IVs [see Eq. 1], which in turn leads to a higher estimate of causal effect when the effect sizes of the IVs of the outcome remain fixed [see Eq. 3]. On the other hand, the female-specific causal effect is underestimated if the PVE of the male exposure is larger than that of the female exposure (3% vs. 1%) (Figures 2D–F).

Moreover, as can be expected, the sex-combined bias generally becomes larger as the sex heterogeneity in IVs increases (i.e., weaker correlation). For example, when true effect size is 0.5 (Figure 2C), the average of the bias is 0.169 for *r*_*g*_ = 0.1, 0.150 for *r*_*g*_ = 0.3, 0.131 for *r*_*g*_ = 0.5, or 0.127 for *r*_*g*_ = 0.7. In contrast, approximate unbiased estimates of causal effects are generated when using sex-specific IVs. Overall, this simulation study explicitly reveals that the use of sex-combined IVs in sex-specific two-sample MR analyses may lead to a biased causal effect estimate in many cases and that the extension of the bias relies on the relative magnitude of the sex-specific effects and the sex-combined effects of the used IVs.

### Causal Effect Estimation With Sex-Combined Instruments

Like most previous studies (Supplementary Tables 1, 2), we first employ sex-combined instruments to evaluate the causal relationship between each anthropometric trait and breast/prostate cancer as an exploratory analysis. With the random-effects IVW method, which can account for instrumental heterogeneity, we identify statistically significant associations between BMI and breast cancer [OR = 0.85, 95% confidence interval (CIs) 0.76∼0.95, *P* = 0.003], between WC and breast cancer (OR = 0.87, 95% CI 0.77∼0.98, *P* = 0.020), as well as between BMI and prostate cancer (OR = 0.87, 95% CI 0.76∼0.98, *P* = 0.022) (Tables 3, 4), indicating that higher BMI can lead to a lower risk for breast/prostate cancer and that higher WC can result in reduced risk for breast cancer. These results are considerably consistent with prior observations (Shu et al., 2018; Kazmi et al., 2019; Qian et al., 2019). After the removal of IVs exhibiting sex heterogeneity in terms of the Cochran’s *Q* test (Supplementary Tables 4, 5), we observe that the association between WC and breast cancer now becomes non-significant (*P* = 0.069), although other causal effects almost remain unchanged because only a few instruments are excluded, indicating that the sex heterogeneity in IVs might have a substantial influence on the statistical inference in sex-specific two-sample MR analyses.

**TABLE 3:** Association of anthropometric trait with the risk of breast cancer using sex-combined and female-specific instruments.

Exposure	Sex-combined IVs				Female-specific IVs				*u* (*P*)
		
	*k*_0_/*k*_1_	PVE (%)	Power (%)	OR (95% CI, *P*)	*k*_0_/*k*_1_	PVE (%)	Power (%)	OR (95% CI, *P*)	
BMI	97/92	1.51	99.9	0.845 (0.756∼0.945, 0.003)	38/36	1.16	100.0	0.763 (0.661∼0.882, 2.52E-04)	4.795 (1.63E-06)
WHR	39/38	0.95	2.8	1.002 (0.841∼1.194, 0.984)	34/34	1.63	6.7	0.985 (0.858∼1.131, 0.833)	0.610 (0.542)
WC	70/66	1.42	98.0	0.866 (0.767∼0.978, 0.020)	25/25	1.04	96.1	0.858 (0.732∼1.004, 0.057)	0.299 (0.765)
HIP	89/86	2.07	6.1	0.988 (0.885∼1.102, 0.823)	41/39	1.57	93.6	0.890 (0.787∼1.006, 0.063)	9.669 (1.25E-20)

*IV, instrumental variable; BMI, body mass index; WHR, waist-to-hip ratio; WC, waist circumference; HIP, hip circumference; MR, Mendelian randomization; PVE, phenotypic variance explained by IVs that were used in our final MR analysis; power, statistical power; OR, odds ratio; CI, confidence internal.*

*Note that *k*_0_ is the number of candidate instruments, while *k*_1_ is the final number of instruments employed in the analysis.*

**TABLE 4:** Association of anthropometric trait with the risk of prostate cancer using sex-combined and male-specific instruments.

Exposure	Sex-combined IVs				Male-specific IVs				*u* (*P*)
		
	*k*_0_/*k*_1_	PVE (%)	Power (%)	OR (95% CI, *P*)	*k*_0_/*k*_1_	PVE (%)	Power (%)	OR (95% CI, *P*)	
BMI	97/60	1.12	81.3	0.865 (0.764∼0.979, 0.022)	30/22	0.77	67.7	0.862 (0.738∼1.007, 0.060)	0.190 (0.849)
WHR	39/21	0.56	59.2	0.854 (0.681∼1.071, 0.172)	3/1	0.04	5.2	1.094 (0.545∼2.198, 0.800)	−0.969 (0.333)
WC	70/50	1.02	14.1	0.954 (0.808∼1.126, 0.577)	29/21	1.15	59.0	0.895 (0.763∼1.050, 0.175)	2.688 (0.007)
HIP	89/64	1.40	5.4	1.016 (0.886∼1.166, 0.817)	31/17	0.83	28.6	1.086 (0.873∼1.350, 0.457)	−1.428 (0.153)

*IV, instrumental variable; BMI, body mass index; WHR, waist-to-hip ratio; WC, waist circumference; HIP, hip circumference; MR, Mendelian randomization; PVE, phenotypic variance explained by IVs that were used in our final MR analysis; power, statistical power; OR, odds ratio; CI, confidence internal.*

*Note that *k*_0_ is the number of candidate instruments, while *k*_1_ is the final number of instruments employed in the analysis.*

### Causal Effect Estimation With Sex-Specific Instruments

We now estimate the causal effect using only sex-specific instruments and show those new results in Tables 3, 4. Several interesting findings are observed. First, it is observed that there exists a significantly positive correlation between the causal effect obtained with sex-specific effects of IVs and that yielded with sex-combined effects (Supplementary Figures 2, 3), with all the correlation coefficients [i.e., ρ in the *u* test in Eq. 5] larger than 0.9. Second, using the sex-specific IVs, we discover distinct discrepancy in the significance of estimated causal effects of anthropometric traits compared with those obtained with the sex-combined IVs. Specifically, although still maintaining similar effect sizes (0.866 vs. 0.858), WC is now only marginally associated with breast cancer (*P* = 0.057) (Table 3). The analogous situation is seen for the association between BMI and prostate cancer, which also becomes marginally significant (*P* = 0.060), although the causal effect is not influenced (0.865 vs. 0.862) (Table 4). Third, when applying sex-specific IVs, significant discrepancies in the magnitude of estimated causal effects are detected for BMI on breast cancer (*P* = 1.63E-6), for HIP on breast cancer (*P* = 1.25E-20), as well as for WC on prostate cancer (*P* = 0.007) compared with those estimated with sex-combined instruments (Tables 3, 4). More specifically, we find that the causal association between BMI and breast cancer is now more pronounced (Table 3). For example, it is shown that per SD increase of BMI can result in about 23.7% (95% CI 11.8%∼33.9%) lower risk of breast cancer when using the female-specific instruments (OR = 0.76) compared with 15.5% (95% CI 5.5%∼24.4%) lower risk of breast cancer if using those sex-combined instruments (OR = 0.85). Here, it needs to be highlighted that the female PVE of BMI is much larger than the male PVE of BMI (1.16 vs. 0.77; see Tables 3, 4), partly explaining why a stronger association was identified when the female-specific effects of instruments were employed. Note that this finding is also consistent with the phenomenon discovered in the simulation above.

### Sensitivity Analyses With Sex-Specific Instruments

**FIGURE 3:**
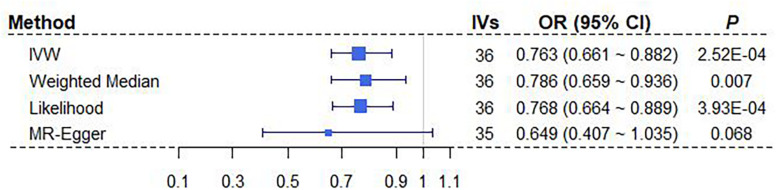
Estimated causal effects of BMI on breast cancer with different MR approaches. MR-Egger regression was performed after removing the instrument of rs17024393. BMI, body mass index; IV, instrumental variable; IVW, inverse-variance weighted; OR, odds ratio; MR, Mendelian randomization.

We here perform a wide series of sensitivity analyses to complement our main MR results obtained above using IVW with sex-specific IVs. Here, only the significant relationship between BMI and breast cancer is considered (Table 3). Both the weighted median method and the maximum likelihood method yield consistent causal effect estimates compared with IVW (Figure 3). We also create a scatter plot to examine whether instrumental outliers are present. Among all these used female-specific instruments, one index SNP (i.e., rs17024393 on gene *GNAT2*) has a large effect size of 0.071 on BMI and may be likely a potential outlier (Supplementary Figure 4A). However, after removing this IV, the OR is estimated to be 0.78 (95% CI 0.67∼0.90, *P* = 5.28E-04) (Supplementary Figure 4C), almost the same as that obtained using all instruments together (OR = 0.76). In addition, the LOO analysis shows that no single instrument can substantially influence the overall IVW estimate (Supplementary Figure 4C). The result of MR-PRESSO displays the absence of instrument outliers at the significance level of 0.05. Finally, the intercept of MR-Egger regression is estimated as 0.006 (95% CI −0.009∼0.022, *P* = 0.418), ruling out the possibility of directional pleiotropic effects of instruments. The funnel plot also presents a symmetric pattern around the overall point estimate (Supplementary Figure 4B), implying that horizontal pleiotropy unlikely biases our result. In conclusion, depending on female-specific IVs, we demonstrate that BMI is robustly negatively associated with breast cancer.

### Power Calculation

Finally, we calculate the statistical power to detect the estimated causal effects of four anthropometric traits on the risk of breast or prostate cancer when applying sex-specific and sex-combined IVs. The power calculation is based on observed causal effect sizes, the number of IVs used, the sample sizes of the exposure, and the outcome. We implement the power calculation via an online software tool available at https://sb452.shinyapps.io/power (Brion et al., 2013).

With regard to the association between WC and breast cancer, the statistical power calculated with female-specific instruments (power = 96.1% when IVs = 25 and PVE = 1.04%) is slightly lower than that computed with sex-combined instruments (power = 98.3% when IVs = 66 and PVE = 1.42%) (Table 3). The analogous situation is seen for the association between BMI and prostate cancer (Table 4). In these analyses, we observe that the number of valid IVs would become smaller after applying sex-specific instruments, which can potentially reduce the statistical power in the MR analysis due to the decrease of PVE.

However, it is also shown that although there is a decrease in the number of valid IVs after using sex-specific instruments, the statistical powers of some associations are higher than those obtained with sex-combined ones (Tables 3, 4). For example, an improvement of statistical power is detected for BMI on breast cancer (power = 100.0% when IVs = 36 vs. power = 99.9% when IVs = 92), for HIP on breast cancer (power = 93.6% when IVs = 39 vs. power = 6.1% when IVs = 86), as well as for WC on prostate cancer (power = 59.9% when IVs = 21 vs. power = 14.1% when IVs = 50) after applying sex-specific instruments. As mentioned before, these analyses show significant discrepancies in the magnitude of estimated causal effects. Therefore, we cannot completely rule out the possibility that the difference in the causal inference is due to the different number of IVs used. However, as shown in our real applications, the impact of sex instrumental heterogeneity on the magnitude of estimated causal effects is substantial, which is not fully driven by the decreased number of IVs.

## Discussion

The main objective of our study was to investigate the influence of sex instrumental heterogeneity on causal effect estimation in sex-specific two-sample MR analyses where in principle sex-specific rather than sex-combined IVs should be employed. One of the common cases is the application of MR to breast cancer or prostate cancer, both of which lead to serious health threat in females and males worldwide (Bray et al., 2018). Therefore, the identification of risk factors for the two types of cancer is important for disease prevention and holds the potential for better therapeutic strategies in the future. More than 80 empirical MR studies have been implemented for the two cancers to date (Supplementary Tables 1, 2); however, only few applied sex-specific IVs for exposures in these analyses (Au Yeung and Schooling, 2019; Jung et al., 2019; Ooi et al., 2019; Li et al., 2020; Mohammadi-Shemirani et al., 2020; Murphy et al., 2020; Ong et al., 2020; Richardson et al., 2020; Ruth et al., 2020; Watts et al., 2020). Particularly, for some exposures (e.g., WHR) that display obvious sex heterogeneity in effect sizes, sex-combined IVs were still employed; even sex-specific summary statistics were publicly available (Gao et al., 2016; Shu et al., 2018). Here, we emphasize again that the issue of sex instrumental heterogeneity specially occurs only in summary statistics-based MR studies, which does not arise when individual-level datasets can be accessible.

In our simulation and empirical analyses, we illustrated that the sex instrumental heterogeneity had a non-ignorable impact on the causal inference and that the causal effects of anthropometric traits on breast/prostate cancer would be greatly influenced if sex-combined IVs were incorrectly applied. To the best of our knowledge, our study is among the first to formally examine the problem of sex-specific instruments in the two-sample MR.

In sex-specific two-sample MR studies, the use of sex-combined instruments makes an implicit hypothesis that no effect differences exist between females and males, which, however, is not always satisfied in terms of previous observations (e.g., Table 1). Nevertheless, in practice, applying sex-combined instruments in MR is not without advantages if such assumption can be well-established. Under this situation, one of the greatest benefits is that more IVs would be exploited because of a larger sample size for the exposure GWAS, which can potentially lead to the improvement of statistical power due to more phenotypic variances explained (e.g., Tables 3, 4).

From a more generalized perspective, the females and the males can be viewed as two diverse populations that have different genetic foundations (Ober et al., 2008) as well as distinct morbidities and mortalities of complex diseases (Wang et al., 2019). Note that to ensure the validity of two-sample MR, one of the important assumptions is that two sample sets should come from the same underlying population. Otherwise, MR may still provide evidence on whether a causal association exists but not necessarily on the precise magnitude of the causal effect (Burgess et al., 2015; Haycock et al., 2016). Our study demonstrated that sex-specific instruments can substantially influence the significance and magnitude of causal effects, confirming the importance of this MR assumption. At the same time, we note that a few sex-specific two-sample MR studies applying sex-specific IVs were published in recent years (Table 1), suggesting the growing attention has been paid on the issue of sex instrumental heterogeneity.

Finally, we offer some suggestions for the issue of sex heterogeneity of instruments when conducting sex-specific two-sample MR studies. First, to guarantee to implement MR in the same population, it is necessary to check the original GWAS of exposures or contact authors to obtain sex-specific summary statistics; a clear statement about whether sex-specific IVs are employed is also highly recommended (Ong et al., 2020; Richardson et al., 2020). Second, when sex-combined instruments are employed, sex heterogeneity in instruments should be carefully examined, followed by extensive sensitivity analyses. When using sex-specific instruments, various sensitivity analyses also should be carried out to ensure the robustness of the results. Third, when only sex-combined summary statistics are available and one has to apply sex-combined IVs for exposures, explaining possible biases in the causal inference due to sex instrumental heterogeneity is strongly advocated (Au Yeung and Schooling, 2019; Jiang et al., 2020; Papadimitriou et al., 2021).

In summary, although the two-sample MR is technically easy to undertake, the principal modeling assumptions should still be validated in sex-specific two-sample MR studies strictly. Especially in the sex-specific two-sample MR analyses, the choice of appropriate IVs for exposures can reduce the bias of causal effect estimation and make MR results more reliable.

## Conclusion

Our study reveals that the sex instrumental heterogeneity may have a non-ignorable impact on sex-specific two-sample MR studies, and the causal effects of anthropometric traits on breast/prostate cancer would be biased if sex-combined IVs are incorrectly employed.

## Data Availability Statement

The datasets presented in this study can be found in online repositories. The names of the repository/repositories and accession number(s) can be found in the article/Supplementary Material.

## Author Contributions

PZ conceived the idea for the study. PZ, FG, and HZ obtained the data. PZ and YG cleared up the datasets, performed the data analyses, and drafted the manuscript. PZ, FG, HZ, YG, and JZ interpreted the results of the data analyses. All authors approved the manuscript and provided relevant suggestions.

## Conflict of Interest

The authors declare that the research was conducted in the absence of any commercial or financial relationships that could be construed as a potential conflict of interest.
