# Supplementary material for: Instrumental Heterogeneity in Sex-Specific Two-Sample Mendelian Randomization: Empirical Results From the Relationship Between Anthropometric Traits and Breast/Prostate Cancer
Source: Front Genet. 2021 Jun 9;12:651332. doi: 10.3389/fgene.2021.651332 (PMC8220153; doi:10.3389/fgene.2021.651332)
Supplement: Supplementary file 1 [file Data_Sheet_1.docx]

**Supplementary Results**

**Supplementary Table 1** Relationship between various exposures and breast cancer in previous MR studies

| Exposure | 1 SD increase | Instruments | Outcome | OR (95%CI, *P*) | | *N* (case/control)  case(events) | Country | Ref of IVs | Ref |
| --- | --- | --- | --- | --- | --- | --- | --- | --- | --- |
| **BMI** | 0.94kg/m^2^ | 94 | ER-positive | 1.11 (1.01~1.22, 0.030) | | 22,567(1,161) | Euro | [[1](#_ENREF_1)] | [[2](#_ENREF_2)] |
|  |  |  | ER-negative | 1.00 (0.89~1.13, 0.950) | | 5,683(679) | Euro |  |  |
| **BMI** | 5kg/m^2^ | 93 | All | 0.93 (0.89~0.97, 7.34E-04) | | 7,516/3,594 | Euro | [[1](#_ENREF_1)] | [[3](#_ENREF_3)] |
|  |  |  | By Mutation status |  | |  |  |  |  |
|  |  |  | BRCA1 carrier | 0.96 (0.91~1.01, 0.110) | | 4,401/2,114 | Euro |  |  |
|  |  |  | BRCA2 carrier | 0.90 (0.84~0.97, 0.003) | | 3,115/1,480 | Euro |  |  |
|  |  |  | By Menopausal Status |  | |  |  |  |  |
|  |  |  | Premenopausal | 0.92 (0.87~0.97, 0.001) | | 7,516/2,153 | Euro |  |  |
|  |  |  | Postmenopausal | 0.97 (0.91~1.04, 0.400) | | 3,029/1,389 | Euro |  |  |
| **BMI** | 1kg/m^2^ | **77 (female)** | All | 0.81 (0.74~0.89, 9.44E-06) | | 122,977/105,974 | Euro | [[1](#_ENREF_1)] | [[4](#_ENREF_4)] |
|  |  |  | ER-positive | 0.79 (0.72~0.88, 4.84E-06) | | 69,501/105,974 | Euro |  |  |
|  |  |  | ER-negative | 0.78 (0.67~0.91, 1.78E-03) | | 21,468/105,974 | Euro |  |  |
| **BMI** | NA | 162 | breast cancer | 0.77 (0.73~0.82, 5.05E-19) | | 98,842/ 83,464 | Euro | [[1](#_ENREF_1), [5](#_ENREF_5)] | [[6](#_ENREF_6)] |
| **childhood BMI** | NA | 15 | All | 0.71 (0.60~0.80, 6.50E-05) | | 15,748/18,084 | Euro | [[7](#_ENREF_7)] | [[8](#_ENREF_8)] |
|  |  |  | ER-negative | 0.69 (0.53~0.98, 5.80E-03) | | 4,939/13,128 | Euro |  |  |
| **adult BMI** | NA | 77 | All | 0.66 (0.57~0.77, 2.50E-07) | | 15,748/18,084 | Euro | [[1](#_ENREF_1)] | [[8](#_ENREF_8)] |
|  |  |  | ER-negative | 0.59 (0.46~0.75, 2.00E-05) | | 4,939/13,128 | Euro |  |  |
| **BMI** | 5kg/m^2^ | 84 | All | 0.65 (0.56~0.75, 3.32E-10) | | 46,325/42,482 | Euro | [[1](#_ENREF_1), [9](#_ENREF_9)] | [[10](#_ENREF_10)] |
|  |  |  | By Menopausal Status | |  |  |  |  |  |
|  |  |  | Premenopausal | 0.44 (0.31~0.62, 9.91E-08) | | 19,262 | Euro |  |  |
|  |  |  | Postmenopausal | 0.57 (0.46~0.71, 1.88E-08) | | 42,424 | Euro |  |  |
|  |  |  | Never HRT use | 0.60 (0.38~0.90, 9.70E-03) | | 11,433 | Euro |  |  |
|  |  |  | Ever HRT use | 0.47 (0.29~0.73, 2.00E-04) | | 10,967 | Euro |  |  |
|  |  |  | By ER Status |  | |  |  |  |  |
|  |  |  | ER-positive | 0.68 (0.57~0.81, 2.74E-06) | | 69,556 | Euro |  |  |
|  |  |  | ER-negative | 0.45 (0.33~0.59, 3.41E-10) | | 49,770 | Euro |  |  |
|  |  |  | By PR status |  | |  |  |  |  |
|  |  |  | PR-positive | 0.65 (0.53~0.78, 9.52E-07) | | 62,231 | Euro |  |  |
|  |  |  | PR-negative | 0.47 (0.36~0.60, 2.84E-11) | | 52,208 | Euro |  |  |
|  |  |  | By ER/PR status |  | |  |  |  |  |
|  |  |  | ER/PR-positive | 0.66 (0.55~0.80, 5.46E-06) | | 61,430 | Euro |  |  |
|  |  |  | ER/PR-negative | 0.42 (0.30~0.58, 7.19E-10) | | 28,855 | Euro |  |  |
| **BMI** | 10kg/m^2^ | 5 | Premenopausal | 0.47 (0.14~1.51, 0.200) | | 20,482 | Danish | [[11](#_ENREF_11)] | [[12](#_ENREF_12)] |
|  |  |  | Postmenopausal | 1.11 (0.34~3.59, 0.860) | | 33,016 | Danish |  |  |
| **adult height** | 10cm | 168 | All | 1.22 (1.13~1.32, 5.29E-07) | | 46,325/42,482 | Euro | [[13-18](#_ENREF_13)] | [[19](#_ENREF_19)] |
|  |  |  | By Menopausal status |  | |  |  |  |  |
|  |  |  | Premenopausal | 1.29 (1.07~1.56, 0.007) | | 10,209/9,053 | Euro |  |  |
|  |  |  | Postmenopausal | 1.32 (1.17~1.49, 6.75E-06) | | 23,069/19,355 | Euro |  |  |
|  |  |  | By ER status | |  |  |  |  |  |
|  |  |  | ER-positive | 1.26 (1.14~1.38, 2.12E-06) | | 27,074/42,482 | Euro |  |  |
|  |  |  | ER-negative | 1.02 (0.87~1.18, 0.840) | | 7,288/42,482 | Euro |  |  |
|  |  |  | By PR status | |  |  |  |  |  |
|  |  |  | PR-positive | 1.23 (1.11~1.37, 1.15E-04) | | 19,749/42,482 | Euro |  |  |
|  |  |  | PR-negative | 1.07 (0.94~1.23, 0.310) | | 9,726/42,482 | Euro |  |  |
|  |  |  | By ER/PR status |  | |  |  |  |  |
|  |  |  | ER/PR-positive | 1.25 (1.12~1.39, 5.12E-05) | | 18,948/42,482 | Euro |  |  |
|  |  |  | ER/PR-negative | 1.03 (0.87~1.23, 0.700) | | 5,848/42,482 | Euro |  |  |
| **height** | 10cm | 423 | breast cancer | 1.19 (1.07~1.33, 0.001) | | 16,003/46,525 | Euro | [[20](#_ENREF_20)] | [[21](#_ENREF_21)] |
| **height** | 10cm | 586 | All | 1.15 (1.07~1.23, 1.60E-04) | | 7,657/3,653 | Euro | [[18](#_ENREF_18), [20](#_ENREF_20)] | [[3](#_ENREF_3)] |
|  |  |  | By Mutation status |  | |  |  |  |  |
|  |  |  | BRCA1 carrier | 1.06 (0.97~1.16, 0.190) | | 4,502/2,154 | Euro |  |  |
|  |  |  | BRCA2 carrier | 1.17 (1.04~1.31, 0.007) | | 3,155/1,499 | Euro |  |  |
|  |  |  | By Menopausal Status |  | |  |  |  |  |
|  |  |  | Premenopausal | 1.10 (1.01~1.20, 0.030) | | 7,657/2,197 | Euro |  |  |
|  |  |  | Postmenopausal | 1.07 (0.95~1.19, 0.260) | | 3,076/1,402 | Euro |  |  |
| **height** | 6.2cm | 691 | breast cancer | 1.07 (1.03~1.11, 7.00E-04) | | 13,396/211,882 | Euro | [[20](#_ENREF_20)] | [[22](#_ENREF_22)] |
| **lipid** |  |  |  |  | |  |  |  |  |
| HDL | NA | 41 | breast cancer | 1.25 (0.99~1.59, 0.065) | | N/cases: 16,022/1,187 | Sweden | [[23](#_ENREF_23)] | [[24](#_ENREF_24)] |
| LDL | NA | 32 | breast cancer | 1.05 (0.84~1.31, 0.667) | | N/cases: 16,022/1,187 | Sweden | [[23](#_ENREF_23)] |  |
| TG | NA | 26 | breast cancer | 0.78 (0.59~1.02, 0.075) | | N/cases: 16,022/1,187 | Sweden | [[23](#_ENREF_23)] |  |
| **lipid** |  |  |  |  | |  |  |  |  |
| HDL-C | 15mg/dL | 74 | All | 1.12 (1.08~1.16, 1.70E-09) | | 101,424/80,253 | Euro | [[23](#_ENREF_23), [25](#_ENREF_25)] | [[26](#_ENREF_26)] |
|  |  |  | By Menopausal Status |  | |  |  |  |  |
|  |  |  | Premenopausal | 1.14 (0.96~1.35, 0.130) | | 20,782/17,902 | Euro |  |  |
|  |  |  | Postmenopausal | 1.11 (1.05~1.17, 3.20E-04) | | 43,787/38,847 | Euro |  |  |
|  |  |  | By ER Status |  | |  |  |  |  |
|  |  |  | ER-negative | 1.10 (1.03~1.18, 0.007) | | 43,039/80,253 | Euro |  |  |
|  |  |  | ER-positive | 1.11 (1.07~1.16, 8.60E-08) | | 61,140/80,253 | Euro |  |  |
| TC | 42mg/dL | 74 | All | 1.05 (0.99~1.11, 0.110) | | 101,424/80,253 | Euro | [[23](#_ENREF_23), [25](#_ENREF_25)] |  |
|  |  |  | By Menopausal Status |  | |  |  |  |  |
|  |  |  | Premenopausal | 1.08 (1.00~1.17, 0.040) | | 20,782/17,902 | Euro |  |  |
|  |  |  | Postmenopausal | 1.00 (0.95~1.05, 0.960) | | 43,787/38,847 | Euro |  |  |
|  |  |  | By ER Status |  | |  |  |  |  |
|  |  |  | ER-negative | 1.06 (0.93~1.21, 0.360) | | 43,039/80,253 | Euro |  |  |
|  |  |  | ER-positive | 1.03 (0.99~1.07, 0.170) | | 61,140/80,253 | Euro |  |  |
| LDL-C | 37mg/dL | 57 | All | 1.00 (0.96~1.04, 0.880) | | 101,424/80,253 | Euro | [[23](#_ENREF_23), [25](#_ENREF_25)] |  |
|  |  |  | By Menopausal Status |  | |  |  |  |  |
|  |  |  | Premenopausal | 1.00 (0.92~1.08, 0.890) | | 20,782/17,902 | Euro |  |  |
|  |  |  | Postmenopausal | 0.99 (0.94~1.05, 0.800) | | 43,787/38,847 | Euro |  |  |
|  |  |  | By ER Status |  | |  |  |  |  |
|  |  |  | ER-negative | 0.99 (0.93~1.05, 0.730) | | 43,039/80,253 | Euro |  |  |
|  |  |  | ER-positive | 0.99 (0.95~1.03, 0.630) | | 61,140/80,253 | Euro |  |  |
| TG | 43mg/dL | 43 | All | 0.93 (0.85~1.01, 0.075) | | 101,424/80,253 | Euro | [[23](#_ENREF_23), [25](#_ENREF_25)] |  |
|  |  |  | By Menopausal Status |  | |  |  |  |  |
|  |  |  | Premenopausal | 0.90 (0.75~1.08, 0.240) | | 20,782/17,902 | Euro |  |  |
|  |  |  | Postmenopausal | 0.93 (0.88~0.99, 0.030) | | 43,787/38,847 | Euro |  |  |
|  |  |  | By ER Status |  | |  |  |  |  |
|  |  |  | ER-negative | 0.94 (0.87~1.01, 0.083) | | 43,039/80,253 | Euro |  |  |
|  |  |  | ER-positive | 0.91 (0.85~0.98, 0.014) | | 61,140/80,253 | Euro |  |  |
| **lipid** |  |  |  |  | |  |  |  |  |
| HDL | NA | 85 | All | 1.10 (1.03~1.17, 0.003) | | 122,977/105,974 | Euro | [[25](#_ENREF_25)] | [[27](#_ENREF_27)] |
|  |  |  | ER-positive | 1.11 (1.04~1.19, 0.002) | | 122,977/105,974 | Euro |  |  |
|  |  |  | ER-negative | 1.09 (1.00~1.19, 0.062) | | 122,977/105,974 | Euro |  |  |
| LDL | NA | 76 | All | 1.05 (1.00~1.10, 0.055) | | 122,977/105,974 | Euro | [[25](#_ENREF_25)] |  |
|  |  |  | ER-positive | 1.04 (0.99~1.10, 0.144) | | 122,977/105,974 | Euro |  |  |
|  |  |  | ER-negative | 1.04 (0.97~1.12, 0.321) | | 122,977/105,974 | Euro |  |  |
| TG | NA | 51 | All | 0.91 (0.83~0.99, 0.026) | | 122,977/105,974 | Euro | [[25](#_ENREF_25)] |  |
|  |  |  | ER-positive | 0.91 (0.83~0.99, 0.036) | | 122,977/105,974 | Euro |  |  |
|  |  |  | ER-negative | 0.90 (0.81~1.00, 0.052) | | 122,977/105,974 | Euro |  |  |
| **lipid** |  |  |  |  | |  |  |  |  |
| HDL | NA | 113 | breast cancer | 1.08 (1.04~1.13, 7.42E-05) | | 122,977/105,974 | Euro | [[28](#_ENREF_28)] | [[29](#_ENREF_29)] |
| LDL | NA | 72 | breast cancer | 1.04 (1.00~1.09, 0.070) | | 122,977/105,974 | Euro | [[28](#_ENREF_28)] |  |
| TG | NA | 97 | breast cancer | 0.94 (0.90~0.98, 2.60E-03) | | 122,977/105,974 | Euro | [[28](#_ENREF_28)] |  |
| **birthweight** | NA | 7 | All | 1.22 (0.93~1.60, 0.150) | | 15,748/18,084 | Euro | [[30](#_ENREF_30)] | [[8](#_ENREF_8)] |
|  |  |  | ER-negative | 1.01 (0.66~1.53, 0.980) | | 4,939/13,128 | Euro |  |  |
| **birth weight** | 500g | 41 | All | 0.86 (0.73~1.01, 0.060) | | 122,977/105,974 | Euro | [[31](#_ENREF_31)] | [[32](#_ENREF_32)] |
|  |  |  | ER-positive | 0.86 (0.72~1.02, 0.080) | | 69,501/105,974 | Euro |  |  |
|  |  |  | ER-negative | 0.92 (0.74~1.14, 0.420) | | 21,468/105,974 | Euro |  |  |
| **waist-hip ratio (WHR)** | NA | 50 | breast cancer | 0.85 (0.79~0.91, 9.22E-06) | | 98,842/ 83,464 | Euro | [[1](#_ENREF_1), [5](#_ENREF_5)] | [[6](#_ENREF_6)] |
| **waist-hip ratio (WHR)** | NA | 14 | All | 0.73 (0.53~1.00, 0.051) | | 15,748/18,084 | Euro | [[33](#_ENREF_33)] | [[8](#_ENREF_8)] |
|  |  |  | ER-negative | 0.74 (0.45~1.21, 0.230) | | 4,939/13,128 | Euro |  |  |
| **body size** |  |  |  |  | |  |  |  |  |
| early life body size | NA | **124 (female)** | All | 0.63 (0.55~0.72, 3.84E-11) | | 122,977/105,974 | Euro | [[34](#_ENREF_34)] | [[35](#_ENREF_35)] |
|  |  |  | By ER Status |  | |  |  |  |  |
|  |  |  | ER-positive | 0.67 (0.58~0.77, 1.43E-07) | | 69,501/105,974 | Euro |  |  |
|  |  |  | ER-negative | 0.57 (0.48~0.68, 6.28E-10) | | 21,468/105,974 | Euro |  |  |
| adulthood body size | NA | **191 (female)** | All | 0.82 (0.73~0.92, 8.04E-04) | | 122,977/105,974 | Euro | [[34](#_ENREF_34)] |  |
|  |  |  | By ER Status |  | |  |  |  |  |
|  |  |  | ER-positive | 0.84 (0.73~0.96, 5.23E-03) | | 69,501/105,974 | Euro |  |  |
|  |  |  | ER-negative | 0.81 (0.68~0.97, 0.016) | | 21,468/105,974 | Euro |  |  |
| **age at menarche (AAM)** | 1 year | 375 (female only) | breast cancer | 1.00 (0.96~1.05, >0.05) | | 47,000/43,000 | Euro | [[36](#_ENREF_36)] | [[37](#_ENREF_37)] |
| **age at menarche (AAM)** | NA | 375 (female only) | All | 0.94 (0.89~0.98, 2.64E-03) | | 47,800/40,302 | Euro | [[38](#_ENREF_38), [39](#_ENREF_39)] | [[36](#_ENREF_36)] |
|  |  |  | ER-positive | 0.93 (0.89~0.98, 6.05E-03) | | 47,800/40,302 | Euro |  |  |
|  |  |  | ER-negative | 0.94 (0.86~1.02, 0.148) | | 47,800/40,302 | Euro |  |  |
| **Vitamin D** | NA | 3 | breast cancer | 1.09 (0.55~2.15, 0.800) | | 1,145/1,142 | Euro | [[40](#_ENREF_40)] | [[41](#_ENREF_41)] |
| **25(OH)D** | 25nmol/L | 4 | All | 1.05 (0.89~1.24, 0.590) | | 15,748/18,084 | Euro | [[42](#_ENREF_42), [43](#_ENREF_43)] | [[44](#_ENREF_44)] |
|  |  |  | ER-negative | 1.15 (0.88~1.50, 0.300) | | 4,939/13,128 | Euro |  |  |
| **25(OH)D** | 25nmol/L | 6 | All | 1.02 (0.97~1.08, 0.470) | | 122,977/105,974 | Euro | [[45](#_ENREF_45)] | [[46](#_ENREF_46)] |
|  |  |  | ER-positive | 1.00 (0.94~1.07, 0.990) | | 69,501/105,974 | Euro |  |  |
|  |  |  | ER-negative | 1.02 (0.90~1.16, 0.750) | | 21,468/105,974 | Euro |  |  |
| **25(OH)D** | 20nmol/L | 5 | breast cancer | 1.02 (0.99~1.05, 0.187) | | 1,560/23,294 | Euro | [[47](#_ENREF_47)] | [[48](#_ENREF_48)] |
| **25(OH)D** | NA | 14 | breast cancer | 0.96 (0.90~1.03, 0.230) | | 1,657/2,029 | African | [[43](#_ENREF_43), [49](#_ENREF_49)] | [[50](#_ENREF_50)] |
| **25(OH)D** | 20nmol/L | 5 | breast cancer | 0.94 (0.85~1.03, 0.190) | | 11,703 | Euro | [[44](#_ENREF_44), [51-53](#_ENREF_51)] | [[54](#_ENREF_54)] |
| **telomere length** | NA | 16 | ER-negative | 1.14 (0.91~1.43, 0.250) | | 7,465/42,175 | Euro | [[55-60](#_ENREF_55)] | [[61](#_ENREF_61)] |
|  |  |  | ER-positive | 1.06 (0.95~1.17, 0.290) | | 27,074/41,749 | Euro |  |  |
| **telomere length** | NA | 9 | All | 1.02 (0.86~1.21, 0.820) | | 15,748/18,084 | Euro | [[55](#_ENREF_55), [56](#_ENREF_56), [58](#_ENREF_58)] | [[62](#_ENREF_62)] |
|  |  |  | ER-negative | 1.05 (0.81~1.38, 0.700) | | 4,939/13,128 | Euro |  |  |
| **telomere length** | NA | 15 | breast cancer | 1.02 (0.99~1.04, 0.164) | | 7,330/307,395 | Euro | [[63](#_ENREF_63)] | [[64](#_ENREF_64)] |
| **schizophrenia (SCZ)** | NA | 170 | All | 1.04 (1.02~1.06, 5.60E-05) | | 122,977/105,974 | Euro | [[65](#_ENREF_65), [66](#_ENREF_66)] | [[67](#_ENREF_67)] |
|  |  |  | ER-positive | 1.04 (1.02~1.07, 2.20E-04) | | 69,501/95,042 | Euro |  |  |
|  |  |  | ER-negative | 1.04 (1.01~1.07, 7.20E-03) | | 21,468/100,594 | Euro |  |  |
| **schizophrenia (SCZ)** | NA | 142 | breast cancer | 1.03 (1.01~1.05, 2.30E-04) | | 122,977/105,974 | Euro | [[66](#_ENREF_66)] | [[68](#_ENREF_68)] |
| **2-h glucose** | 2mmol/L | 4 | breast cancer | 1.80 (1.30~2.49, 4.02E-04) | | 98,842/ 83,464 | Euro | [[69](#_ENREF_69), [70](#_ENREF_70)] | [[6](#_ENREF_6)] |
| **fasting insulin** | 0.6ln[pmol/L] | 10 | breast cancer | 1.71 (1.26~2.31, 5.09E-04) | | 98,842/ 83,464 | Euro | [[69](#_ENREF_69), [70](#_ENREF_70)] |  |
| **fasting glucose** | 0.65mmol/L | 31 | breast cancer | 1.02 (0.91~1.14, 0.749) | | 98,842/ 83,464 | Euro | [[69](#_ENREF_69), [70](#_ENREF_70)] |  |
| **fasting glucose** | 1mmol/L | **21 (female)** | All | 1.03 (0.85~1.25, 0.764) | | 122,977/105,974 | Euro | [[71](#_ENREF_71)] | [[72](#_ENREF_72)] |
|  |  | **21 (female)** | All | 1.05 (0.82~1.33, 0.693) | | 12,671/388,549 | Euro |  |  |
|  |  |  | By ER Status |  | |  |  |  |  |
|  |  | **21 (female)** | ER-positive | 1.03 (0.85~1.24, 0.759) | | 69,501/95,042 | Euro |  |  |
|  |  | **21 (female)** | ER-negative | 1.06 (0.82~1.38, 0.661) | | 21,468/100,594 | Euro |  |  |
| **HbA1c** | 1% | 32 | All | 1.02 (0.73~1.45, 0.910) | | 122,977/105,974 | Euro | [[71](#_ENREF_71)] |  |
|  |  | 34 | All | 1.08 (0.64~1.81, 0.772) | | 12,671/388,549 | Euro |  |  |
|  |  |  | By ER Status |  | |  |  |  |  |
|  |  | 32 | ER-positive | 1.06 (0.75~1.50, 0.742) | | 69,501/95,042 | Euro |  |  |
|  |  | 32 | ER-negative | 1.12 (0.68~1.84, 0.655) | | 21,468/100,594 | Euro |  |  |
| **insulin resistance (IR)** | NA | **12 (female)** | breast cancer | 0.93 (0.76~1.14, 0.483) | | 11,109 | non-Hispanic white postmenopausal women | [[73](#_ENREF_73)] | [[74](#_ENREF_74)] |
| **IGF-1** | 5nmol/L | **265 (female)** | All | 1.05 (1.01~1.10, 0.020) | | 122,977/105,974 | Euro | 2019 UK Biobank GWAS results | [[75](#_ENREF_75)] |
|  |  |  | By ER Status |  | |  |  |  |  |
|  |  |  | ER-positive | 1.06 (1.01~1.11, 0.030) | | 69,501/105,974 | Euro |  |  |
|  |  |  | ER-negative | 1.02 (0.96~1.08, 0.580) | | 21,468/105,974 | Euro |  |  |
| **IGF-1** | NA | 416 | breast cancer | 1.08 (1.02~1.13, 4.40E-03) | | 122,977/105,974 | Euro | [[76](#_ENREF_76)] | [[77](#_ENREF_77)] |
|  |  |  | breast cancer | 0.99 (0.92~1.07, 0.850) | | 13,666/185,172 | Euro | [[76](#_ENREF_76)] |  |
| **IGFBP-3** | 863ng/mL | 4 | All | 1.00 (0.97~1.04, 0.955) | | 122,977/105,974 | Euro | [[78](#_ENREF_78)] |  |
|  |  |  | By ER Status |  | |  |  |  |  |
|  |  |  | ER-positive | 1.00 (0.96~1.05, 0.965) | | 69,501/105,974 | Euro |  |  |
|  |  |  | ER-negative | 0.98 (0.92~1.04, 0.518) | | 21,468/105,974 | Euro |  |  |
| **iron status** |  |  |  |  | |  |  |  |  |
| Transferritin | NA | 8 | All | 1.01 (0.96~1.06, 0.750) | | 122,977/105,974 | Euro | [[79](#_ENREF_79)] | [[80](#_ENREF_80)] |
|  |  |  | All | 0.94 (0.81~1.10, 0.470) | | 13,666 | Euro |  |  |
|  |  |  | By ER Status |  | |  |  |  |  |
|  |  |  | ER-positive | 0.98 (0.93~1.05, 0.600) | | 69,501/105,974 | Euro |  |  |
|  |  |  | ER-negative | 1.07 (0.98~1.18, 0.140) | | 21,468/105,974 | Euro |  |  |
| Serum iron | NA | 5 | All | 0.99 (0.94~1.03, 0.620) | | 122,977/105,974 | Euro | [[79](#_ENREF_79)] |  |
|  |  |  | All | 1.10 (1.00~1.22, 0.050) | | 13,666 | Euro |  |  |
|  |  |  | By ER Status |  | |  |  |  |  |
|  |  |  | ER-positive | 1.01 (0.96~1.07, 0.730) | | 69,501/105,974 | Euro |  |  |
|  |  |  | ER-negative | 0.93 (0.85~1.01, 0.070) | | 21,468/105,974 | Euro |  |  |
| Transferrin saturation | NA | 5 | All | 0.99 (0.96~1.03, 0.650) | | 122,977/105,974 | Euro | [[79](#_ENREF_79)] |  |
|  |  |  | All | 1.06 (0.98~1.16, 0.160) | | 13,666 | Euro |  |  |
|  |  |  | By ER Status |  | |  |  |  |  |
|  |  |  | ER-positive | 1.00 (0.97~1.03, 0.850) | | 69,501/105,974 | Euro |  |  |
|  |  |  | ER-negative | 0.95 (0.89~1.01, 0.080) | | 21,468/105,974 | Euro |  |  |
| Ferritin | NA | 6 | All | 0.98 (0.88~1.08, 0.660) | | 122,977/105,974 | Euro | [[79](#_ENREF_79)] |  |
|  |  |  | All | 1.18 (0.89~1.56, 0.250) | | 13,666 | Euro |  |  |
|  |  |  | By ER Status |  | |  |  |  |  |
|  |  |  | ER-positive | 1.03 (0.91~1.17, 0.630) | | 69,501/105,974 | Euro |  |  |
|  |  |  | ER-negative | 0.85 (0.70~1.02, 0.090) | | 21,468/105,974 | Euro |  |  |
| **magnesium** | 0.08mmol/L | 6 | breast cancer | 1.17 (1.10~1.25, 9.10E-07) | | 122,977/105,974 | Euro | [[81](#_ENREF_81)] | [[82](#_ENREF_82)] |
| **circulating adipokines** |  |  |  |  | |  |  |  |  |
| Interleukin-6 | NA | 3 | All | 1.09 (0.96~1.25, 0.180) | | 122,977/105,974 | Euro | [[83](#_ENREF_83)] | [[84](#_ENREF_84)] |
|  |  |  | By ER Status |  | |  |  |  |  |
|  |  |  | ER-positive | 1.12 (0.96~1.31, 0.140) | | 69,501/105,974 | Euro |  |  |
|  |  |  | ER-negative | 1.00 (0.79~1.27, 0.990) | | 21,468/105,974 | Euro |  |  |
| Adiponectin | NA | 8 | All | 1.06 (0.81~1.40, 0.660) | | 122,977/105,974 | Euro | [[85](#_ENREF_85)] |  |
|  |  |  | By ER Status |  | |  |  |  |  |
|  |  |  | ER-positive | 0.98 (0.71~1.35, 0.910) | | 69,501/105,974 | Euro |  |  |
|  |  |  | ER-negative | 1.19 (0.92~1.54, 0.180) | | 21,468/105,974 | Euro |  |  |
| PAI-1 | NA | 3 | All | 1.03 (0.80~1.33, 0.830) | | 122,977/105,974 | Euro | [[86](#_ENREF_86)] |  |
|  |  |  | By ER Status |  | |  |  |  |  |
|  |  |  | ER-positive | 0.98 (0.78~1.24, 0.870) | | 69,501/105,974 | Euro |  |  |
|  |  |  | ER-negative | 1.05 (0.65~1.68, 0.850) | | 21,468/105,974 | Euro |  |  |
| HGF | NA | 1 | All | 1.01 (0.93~1.10, 0.770) | | 122,977/105,974 | Euro | [[87](#_ENREF_87)] |  |
|  |  |  | By ER Status |  | |  |  |  |  |
|  |  |  | ER-positive | 1.01 (0.92~1.11, 0.860) | | 69,501/105,974 | Euro |  |  |
|  |  |  | ER-negative | 1.17 (1.01~1.35, 0.035) | | 21,468/105,974 | Euro |  |  |
| Leptin receptor | NA | 1 | All | 1.00 (0.99~1.01, 0.630) | | 122,977/105,974 | Euro | [[87](#_ENREF_87)] |  |
|  |  |  | By ER Status |  | |  |  |  |  |
|  |  |  | ER-positive | 1.00 (0.99~1.01, 0.810) | | 69,501/105,974 | Euro |  |  |
|  |  |  | ER-negative | 1.00 (0.98~1.02, 0.780) | | 21,468/105,974 | Euro |  |  |
| Resistin | NA | 1 | All | 0.98 (0.91~1.04, 0.480) | | 122,977/105,974 | Euro | [[87](#_ENREF_87)] |  |
|  |  |  | By ER Status |  | |  |  |  |  |
|  |  |  | ER-positive | 0.98 (0.91~1.06, 0.610) | | 69,501/105,974 | Euro |  |  |
|  |  |  | ER-negative | 0.99 (0.87~1.11, 0.810) | | 21,468/105,974 | Euro |  |  |
| **C-reactive protein** | NA | 45 | All | 1.03 (0.81~1.40, 0.660) | | 122,977/105,974 | Euro | [[88](#_ENREF_88)] | [[84](#_ENREF_84)] |
|  |  |  | By ER Status |  | |  |  |  |  |
|  |  |  | ER-positive | 0.98 (0.71~1.35, 0.910) | | 69,501/105,974 | Euro |  |  |
|  |  |  | ER-negative | 1.19 (0.92~1.54, 0.180) | | 21,468/105,974 |  |  |  |
| **cytokines** |  |  |  |  | |  |  |  |  |
| MCP1 | NA | **10 (female)** | All | 1.08 (1.03~1.12, 3.55E-04) | | 122,977/105,974 | Euro | [[89](#_ENREF_89)] | [[90](#_ENREF_90)] |
|  |  |  | By ER Status |  | |  |  |  |  |
|  |  |  | ER-positive | 1.08 (1.03~1.13, 0.003) | | 69,501/105,974 | Euro |  |  |
|  |  |  | ER-negative | 1.05 (0.95~1.15, 0.348) | | 21,468/105,974 | Euro |  |  |
| MIP1b | NA | **88 (female)** | All | 1.02 (1.01~1.04, 0.003) | | 122,977/105,974 | Euro | [[89](#_ENREF_89)] |  |
|  |  |  | By ER Status |  | |  |  |  |  |
|  |  |  | ER-positive | 1.02 (1.00~1.04, 0.012) | | 69,501/105,974 | Euro |  |  |
|  |  |  | ER-negative | 1.04 (1.02~1.06, 0.001) | | 21,468/105,974 | Euro |  |  |
| IL13 | NA | **5 (female)** | All | 1.06 (1.03~1.10, 3.33E-04) | | 122,977/105,974 | Euro | [[89](#_ENREF_89)] |  |
|  |  |  | By ER Status |  | |  |  |  |  |
|  |  |  | ER-positive | 1.05 (1.01~1.09, 0.024) | | 69,501/105,974 | Euro |  |  |
|  |  |  | ER-negative | 1.08 (1.02~1.15, 0.008) | | 21,468/105,974 | Euro |  |  |
| **tumour necrosis factor** | NA | 4 | All | 0.51 (0.39~0.67, 1.20E-06) | | 122,977/105,974 | Euro | Inflammatory biomarker genomics: from discovery to causality | [[91](#_ENREF_91)] |
|  |  |  | By ER Status |  | |  |  |  |  |
|  |  |  | ER-positive | 0.48 (0.35~0.66, 8.00E-06) | | 69,501/105,974 | Euro |  |  |
|  |  |  | ER-negative | 0.54 (0.33~0.88, 0.013) | | 21,468/105,974 | Euro |  |  |
| **Arachidonic acid concentrations** | NA | 2 | breast cancer | 1.01 (0.98~1.04, 0.659) | | 122,977/105,974 | Euro | [[92](#_ENREF_92)] | [[93](#_ENREF_93)] |
|  |  |  | breast cancer | 0.98 (0.95~1.01, 0.108) | | 13,666/185,172 | Euro |  |  |
| **Thyroid dysfunction** |  |  |  |  | |  |  | [[94](#_ENREF_94)] | [[95](#_ENREF_95)] |
| TSH | NA | 58 | All | 0.93 (0.86~1.00, 0.045) | | 13,666 | Euro |  |  |
|  |  |  | All | 0.95 (0.90~1.00, 0.064) | | 122,977/105,974 | Euro |  |  |
|  |  |  | By ER Status |  | |  |  |  |  |
|  |  |  | ER-positive | 0.95 (0.90~1.00, 0.066) | | 69,501/105,974 | Euro |  |  |
|  |  |  | ER-negative | 1.02 (0.95~1.09, 0.585) | | 21,468/105,974 | Euro |  |  |
| Hypothyroidism | NA | 7 | All | 0.92 (0.84~1.00, 0.045) | | 13,666 | Euro |  |  |
|  |  |  | All | 0.97 (0.92~1.03, 0.303) | | 122,977/105,974 | Euro |  |  |
|  |  |  | By ER Status |  | |  |  |  |  |
|  |  |  | ER-positive | 0.98 (0.93~1.04, 0.553) | | 69,501/105,974 | Euro |  |  |
|  |  |  | ER-negative | 0.95 (0.91~1.00, 0.067) | | 21,468/105,974 | Euro |  |  |
| Hyperthyroidism | NA | 8 | All | 1.08 (1.03~1.12, 0.001) | | 13,666 | Euro |  |  |
|  |  |  | All | 1.03 (1.00~1.06, 0.053) | | 122,977/105,974 | Euro |  |  |
|  |  |  | By ER Status |  | |  |  |  |  |
|  |  |  | ER-positive | 1.04 (1.00~1.07, 0.026) | | 69,501/105,974 | Euro |  |  |
|  |  |  | ER-negative | 0.99 (0.96~1.03, 0.670) | | 21,468/105,974 | Euro |  |  |
| Free thyroxine | NA | 31 | All | 1.05 (0.96~1.16, 0.269) | | 13,666 | Euro |  |  |
|  |  |  | All | 1.07 (1.02~1.13, 0.006) | | 122,977/105,974 | Euro |  |  |
|  |  |  | By ER Status |  | |  |  |  |  |
|  |  |  | ER-positive | 1.08 (1.02~1.14, 0.005) | | 69,501/105,974 | Euro |  |  |
|  |  |  | ER-negative | 1.04 (0.96~1.13, 0.297) | | 21,468/105,974 | Euro |  |  |
| **IDO1** | NA | 3 | breast cancer | 0.99 (0.97~1.02, 0.540) | | 122,977/105,974 | Euro | [[87](#_ENREF_87)] | [[96](#_ENREF_96)] |
| **KAT3** | NA | 2 | breast cancer | 1.03 (0.98~1.08, 0.190) | | 122,977/105,974 | Euro | [[87](#_ENREF_87)] |  |
| **GDF-15** | NA | 5 | breast cancer | 0.89 (0.82~0.96, 0.004) | | 12,898/388,549 | Euro | [[97](#_ENREF_97)] | [[98](#_ENREF_98)] |
|  |  |  | breast cancer | 0.97 (0.94~1.01, 0.096) | | 122,977/105,974 | Euro |  |  |
| **pigmentation phenotypes** | NA | 17 |  |  | |  |  | [[99-111](#_ENREF_99)] | [[112](#_ENREF_112)] |
| Hair color |  |  |  |  | |  |  |  |  |
| dark hair |  |  | breast cancer |  | | 1,301/1,292 | Spanish |  |  |
| Light brown hair |  |  | breast cancer | 0.93 (0.73~1.17, 0.518) | | 266/301 | Spanish |  |  |
| blonde hair |  |  | breast cancer | 1.11 (0.81~1.53, 0.518) | | 155/149 | Spanish |  |  |
| Skin color |  |  |  |  | |  |  |  |  |
| Dark skin |  |  | breast cancer |  | | 243/277 | Spanish |  |  |
| Light brown skin |  |  | breast cancer | 0.93 (0.77~1.13, 0.461) | | 626/681 | Spanish |  |  |
| Fair skin |  |  | breast cancer | 0.93 (0.70~1.24, 0.627) | | 864/922 | Spanish |  |  |
| Eye color |  |  |  |  | |  |  |  |  |
| Black/dark brown |  |  | breast cancer |  | | 987/967 | Spanish |  |  |
| Light brown/green |  |  | breast cancer | 0.75 (0.61~0.91, 0.005) | | 509/622 | Spanish |  |  |
| Blue/grey |  |  | breast cancer | 1.09 (0.84~1.41, 0.531) | | 239/292 | Spanish |  |  |
| Freckles |  |  |  |  | |  |  |  |  |
| No |  |  | breast cancer |  | | 472/578 | Spanish |  |  |
| Yes |  |  | breast cancer | 1.09 (0.89~1.32, 0.410) | | 1,254/1,303 | Spanish |  |  |
| **sleep traits** |  |  |  |  | |  |  |  |  |
| Chronotype | 1 category ^a^ | 305 | All | 0.88 (0.82~0.93, 6.38E-05) | | 122,977/105,974 | Euro | [[113](#_ENREF_113)] | [[114](#_ENREF_114)] |
|  |  |  | ER-positive | 0.86 (0.80~0.92, 2.32E-05) | | 69,501/105,974 | Euro |  |  |
|  |  |  | ER-negative | 0.88 (0.80~0.97, 0.010) | | 21,468/105,974 | Euro |  |  |
| Sleep duration | 1 hour | 82 | All | 1.19 (1.02~1.39, 0.030) | | 122,977/105,974 | Euro | [[115](#_ENREF_115)] |  |
|  |  |  | ER-positive | 1.21 (1.01~1.45, 0.036) | | 69,501/105,974 | Euro |  |  |
|  |  |  | ER-negative | 1.22 (1.03~1.45, 0.025) | | 21,468/105,974 | Euro |  |  |
| Insomnia | 1 category ^b^ | 50 | All | 0.80 (0.49~1.31, 0.370) | | 122,977/105,974 | Euro | [[116](#_ENREF_116)] |  |
|  |  |  | ER-positive | 0.70 (0.40~1.21, 0.200) | | 69,501/105,974 | Euro |  |  |
|  |  |  | ER-negative | 0.92 (0.55~1.54, 0.760) | | 21,468/105,974 | Euro |  |  |
| **breast size** | 1 unit of bra cup size | 7 (female only) | All | 1.23 (0.78~1.95, 0.370) | | 122,977/105,974 | Euro | [[117](#_ENREF_117)] | [[4](#_ENREF_4)] |
|  |  |  | ER-positive | 1.19 (0.81~1.74, 0.373) | | 69,501/105,974 | Euro |  |  |
|  |  |  | ER-negative | 1.32 (0.69~2.55, 0.405) | | 21,468/105,974 | Euro |  |  |
| **testosterone** |  |  |  |  | |  |  |  |  |
| bioavailable testosterone | 3.7nmol/L | **176 (female)** | All | 1.15 (1.05~1.25, 1.60E-03) | | 122,977/105,974 | Euro | [[118-121](#_ENREF_118)] | [[122](#_ENREF_122)] |
|  |  |  | By ER Status |  | |  |  |  |  |
|  |  |  | ER-positive | 1.23 (1.12~1.34, 6.10E-06) | | 69,501/95,039 | Euro |  |  |
|  |  |  | ER-negative | 0.94 (0.83~1.07, 0.340) | | 21,468/100,594 | Euro |  |  |
| total testosterone | 3.7nmol/L | **245 (female)** | All | 1.14 (1.08~1.20, 1.60E-06) | | 122,977/105,974 | Euro | [[118-121](#_ENREF_118)] |  |
|  |  |  | By ER Status |  | |  |  |  |  |
|  |  |  | ER-positive | 1.19 (1.12~1.26, 1.20E-08) | | 69,501/95,039 | Euro |  |  |
|  |  |  | ER-negative | 0.99 (0.93~1.06, 0.880) | | 21,468/100,594 | Euro |  |  |
| **sex hormone binding globulin (SHBG)** | 25nmol/L | 12 | All | 0.94 (0.90~0.98, 0.010) | | 122,977/105,974 | Euro | [[119](#_ENREF_119)] | [[123](#_ENREF_123)] |
|  |  |  | ER-positive | 0.92 (0.87~0.97, 0.003) | | 69,501/95,042 | Euro |  |  |
|  |  |  | ER-negative | 1.09 (1.00~1.18, 0.041) | | 21,468/100,594 | Euro |  |  |
| **smoking initiation** | NA | 378 | All | 1.05 (1.01~1.09, 0.020) | | 122,977/105,974 | Euro | [[124](#_ENREF_124)] | [[125](#_ENREF_125)] |
|  |  |  | By ER Status |  | |  |  |  |  |
|  |  |  | ER-positive | 1.05 (1.01~1.10, 0.030) | | 69,501/105,974 | Euro |  |  |
|  |  |  | ER-negative | 1.04 (0.98~1.12, 0.210) | | 21,468/105,974 | Euro |  |  |
| **smoking initiation** | NA | 378 | breast cancer | 1.05 (0.99~1.12, 0.120) | | 122,977/105,974 | Euro | [[124](#_ENREF_124)] | [[126](#_ENREF_126)] |
|  |  |  | breast cancer | 0.97 (0.90~1.06, 0.513) | | 13,666/185,172 | Euro |  |  |
| **Drink** |  |  |  |  | |  |  |  |  |
| drinks per week | NA | 99 | All | 1.01 (0.85~1.21, 0.890) | | 122,977/105,974 | Euro | [[124](#_ENREF_124)] | [[127](#_ENREF_127)] |
|  |  |  | By ER Status |  | |  |  |  |  |
|  |  |  | ER-positive | 0.96 (0.78~1.17, 0.660) | | 69,501/95,042 | Euro |  |  |
|  |  |  | ER-negative | 0.96 (0.76~1.21, 0.740) | | 21,468/100,594 | Euro |  |  |
| AUD | NA | 9 (male) | All | 1.04 (0.89~1.21, 0.620) | | 122,977/105,974 | Euro | [[128](#_ENREF_128)] |  |
|  |  |  | By ER Status |  | |  |  |  |  |
|  |  |  | ER-positive | 1.02 (0.88~1.20, 0.760) | | 69,501/95,042 | Euro |  |  |
|  |  |  | ER-negative | 1.01 (0.84~1.23, 0.880) | | 21,468/100,594 | Euro |  |  |
| AUDIT-C | NA | 13 (male) | All | 1.07 (0.90~1.28, 0.440) | | 122,977/105,974 | Euro | [[128](#_ENREF_128)] |  |
|  |  |  | By ER Status |  | |  |  |  |  |
|  |  |  | ER-positive | 1.05 (0.88~1.25, 0.600) | | 69,501/95,042 | Euro |  |  |
|  |  |  | ER-negative | 1.07 (0.85~1.35, 0.560) | | 21,468/100,594 | Euro |  |  |
| **alcohol consumption** | 10g/day | 99 | breast cancer | 1.10 (0.87~1.40, 0.423) | | 13,666/185,172 | Euro | [[124](#_ENREF_124)] | [[126](#_ENREF_126)] |
|  |  |  | breast cancer | 0.99 (0.83~1.18, 0.922) | | 122,977/105,974 | Euro |  |  |
| **alcohol consumption** | 12g/day | **34 (female)** | breast cancer | 1.03 (0.93~1.14, 0.530) | | 122,977/105,974 | Euro | [[129](#_ENREF_129)] | [[130](#_ENREF_130)] |
| **milk intake** | 17.1g/day | 1 | breast cancer | 1.03 (1.00~1.06, 0.080) | | 13,666/185,172 | Euro | [[131](#_ENREF_131), [132](#_ENREF_132)] | [[133](#_ENREF_133)] |
|  |  |  | breast cancer | 1.00 (0.99~1.02, 0.517) | | 122,977/105,974 | Euro |  |  |
| **coffee consumption** | per cup/day coffee intake | 35 | breast cancer | 1.04 (0.95~1.14, 0.399) | | 11,703/141,351 | Euro | [[134](#_ENREF_134)] | [[135](#_ENREF_135)] |
| **obstructive sleep apnea syndrome (OSAS)** | NA | 5 | breast cancer | 1.33 (1.13~1.56, 6.00E-04) | | 6,269/6,624 | Asian | [[136](#_ENREF_136), [137](#_ENREF_137)] | [[138](#_ENREF_138)] |
| **polycystic ovary syndrome** | NA | 13 (female only) | All | 1.08 (1.05~1.12, 7.98E-07) | | 122,977/105,974 | Euro | [[139](#_ENREF_139)] | [[140](#_ENREF_140)] |
|  |  |  | By ER Status |  | |  |  |  |  |
|  |  |  | ER-positive | 1.10 (1.06~1.14, 3.78E-07) | | 69,501/105,974 | Euro |  |  |
|  |  |  | ER-negative | 1.01 (0.95~1.07, 0.675) | | 21,468/105,974 | Euro |  |  |
| **polycystic ovary syndrome** | NA | 11 (female only) | All | 1.07 (1.02~1.12, 0.005) | | 122,977/105,974 | Euro | [[139](#_ENREF_139)] | [[141](#_ENREF_141)] |
|  |  |  | By ER Status |  | |  |  |  |  |
|  |  |  | ER-positive | 1.09 (1.03~1.15, 0.002) | | 69,501/105,974 | Euro |  |  |
|  |  |  | ER-negative | 1.02 (0.96~1.09, 0.463) | | 21,468/105,974 | Euro |  |  |
| **allergic disease** | NA | 132 | All | 1.00 (0.96~1.04, 0.950) | | 122,977/105,974 | Euro | [[142](#_ENREF_142), [143](#_ENREF_143)] | [[144](#_ENREF_144)] |
|  |  |  | By ER Status |  | |  |  |  |  |
|  |  |  | ER-positive | 0.99 (0.95~1.04, 0.710) | | 69,501/95,042 | Euro |  |  |
|  |  |  | ER-negative | 1.05 (0.99~1.10, 0.110) | | 21,468/100,594 | Euro |  |  |
| **type 2 diabetes** | NA | 95 | All | 1.10 (1.04~1.18, 0.010) | | 86,627(7,054) | Euro | [[145](#_ENREF_145)] | [[146](#_ENREF_146)] |
|  |  |  | By ER Status |  | |  |  |  |  |
|  |  | 95 | ER-positive | 1.09 (1.01~1.18, 0.040) | | 59,378(4,246) | Euro |  |  |
|  |  | 95 | ER-negative | 1.09 (0.97~1.24, 0.150) | | 13,692(1,733) | Euro |  |  |
| **type 2 diabetes** | NA | 399 | breast cancer | 1.00 (0.97~1.03, 0.793) | | 13,666/353,977 | Euro | [[147](#_ENREF_147)] | [[148](#_ENREF_148)] |
|  |  |  | All | 1.00 (0.98~1.02, 0.880) | | 122,977/105,974 | Euro |  |  |
|  |  |  | By ER Status |  | |  |  |  |  |
|  |  |  | ER-positive | 1.00 (0.97~1.03, 0.986) | | 69,501/105,974 | Euro |  |  |
|  |  |  | ER-negative | 0.99 (0.96~1.01, 0.310) | | 21,468/105,974 | Euro |  |  |
| **type 2 diabetes** | NA | 147 | All | 0.98 (0.95~1.01, 0.196) | | 122,977/105,974 | Euro | [[147](#_ENREF_147)] | [[72](#_ENREF_72)] |
|  |  | 159 | All | 0.98 (0.95~1.03, 0.327) | | 12,671/388,549 | Euro |  |  |
|  |  |  | By ER Status |  | |  |  |  |  |
|  |  | 147 | ER-positive | 0.98 (0.95~1.02, 0.265) | | 69,501/95,042 | Euro |  |  |
|  |  | 147 | ER-negative | 0.97 (0.93~1.01, 0.148) | | 21,468/100,594 | Euro |  |  |
| **rheumatoid arthritis** | NA | 25 | breast cancer | 1.01 (1.00~1.03, 0.187) | | 122,977/105,974 | Euro | [[149-154](#_ENREF_149)] | [[155](#_ENREF_155)] |
|  |  | 24 | breast cancer | 0.95 (0.91~0.99, 0.017) | | 5,552/89,731 | Asian |  |  |
| **vitiligo** | NA | 37 | All | 0.98 (0.97~0.99, 0.047) | | 122,977/105,974 | Euro | [[156](#_ENREF_156), [157](#_ENREF_157)] | [[158](#_ENREF_158)] |
|  |  |  | By ER Status |  | |  |  |  |  |
|  |  |  | ER-positive | 0.99 (0.97~1.01, 0.249) | | 69,501/105,974 | Euro |  |  |
|  |  |  | ER-negative | 0.97 (0.95~0.99, 0.002) | | 21,468/105,974 | Euro |  |  |
| **physical activity** | 8.14milli-gravities | 5 | All | 0.51 (0.27~0.98, 0.040) | | 122,977/105,974 | Euro | [[159](#_ENREF_159)] | [[160](#_ENREF_160)] |
|  |  |  | By ER Status |  | |  |  |  |  |
|  |  |  | ER-positive | 0.45 (0.20~1.01, 0.054) | | 69,501/105,974 | Euro |  |  |
|  |  |  | ER-negative | 0.95 (0.44~2.04, 0.890) | | 21,468/105,974 | Euro |  |  |
|  |  | 10 | All | 0.59 (0.42~0.84, 0.003) | | 122,977/105,974 | Euro |  |  |
|  |  |  | By ER Status |  | |  |  |  |  |
|  |  |  | ER-positive | 0.53 (0.35~0.82, 0.004) | | 69,501/105,974 | Euro |  |  |
|  |  |  | ER-negative | 0.78 (0.51~1.22, 0.270) | | 21,468/105,974 | Euro |  |  |

^a^ from definite evening, intermediate evening, neither, intermediate morning, definite morning; ^b^ from none, some, frequent.

MR, Mendelian randomization; BMI, body mass index; HDL, high density lipoprotein; LDL, low density lipoprotein; TG, triglycerides; ER, estrogen receptor; PR, progesterone receptor; HRT, hormone replacement therapy; IGF-1, insulin-like growth factor-1; IGFBP-3, insulin-like growth factor-binding protein-3; PAI-1, plasminogen activator inhibitor-1; HGF, hepatocyte growth factor; TSH, thyroid-stimulating hormone level; AUD, alcohol use disorder; AUDIT-C, age-adjusted alcohol use disorder identification test; SD, standard deviation; OR, odds ratio; CI, confidence internal; *P*, *P* value; *N*, sample size; Euro, European; IV, instrumental variable; Ref, reference.

**Supplementary Table 2** Relationship between various exposures and prostate cancer in previous MR stues

| Exposure | 1 SD increase | Instruments | Outcome | OR (95%CI, *P*) | *N* (case/control)  case(events) | Country | Ref of IVs | Ref |
| --- | --- | --- | --- | --- | --- | --- | --- | --- |
| **BMI** | 10kg/m^2^ | 5 | prostate cancer | 2.46 (0.34~17.80, 0.370) | 43,958 | Danish | [[11](#_ENREF_11)] | [[12](#_ENREF_12)] |
| **childhood BMI** | NA | 15 | All | 1.01 (0.83~1.22, 0.910) | 14,160/12,724 | Euro | [[7](#_ENREF_7)] | [[8](#_ENREF_8)] |
|  |  |  | Aggressive | 1.10 (0.83~1.45, 0.490) | 4,450/12,724 | Euro |  |  |
| **adult BMI** | NA | 77 | All | 1.01 (0.84~1.21, 0.970) | 14,160/12,724 | Euro | [[1](#_ENREF_1)] | [[8](#_ENREF_8)] |
|  |  |  | Aggressive | 1.11 (0.85~1.44, 0.440) | 4,450/12,724 | Euro |  |  |
| **BMI** | 1kg/m^2^ | 32 | All | 0.98 (0.96~1.00, 0.070) | 20,848/20,214 | Euro | [[11](#_ENREF_11)] | [[161](#_ENREF_161)] |
|  |  |  | Localized PCA | 0.98 (0.96~1.00, 0.052) | 12,975/20,214 | Euro |  |  |
|  |  |  | Advanced PCA | 1.01 (0.97~1.05, 0.620) | 4,325/20,214 | Euro |  |  |
|  |  |  | Low-grade PCA | 0.97 (0.94~1.00, 0.090) | 8,784/20,214 | Euro |  |  |
|  |  |  | High-grade PCA | 1.00 (0.98~1.01, 0.650) | 8,230/20,214 | Euro |  |  |
| **BMI** | 1kg/m^2^ | 535 | prostate cancer | 0.90 (0.84~0.97, 0.003) | 79,148/61,106 | Euro | [[162](#_ENREF_162)] | [[163](#_ENREF_163)] |
| **height** | 1m | 426 | Aggressive PCA | 1.07 (1.01~1.15, 0.030) | 15,167/58,308 | Euro | [[20](#_ENREF_20)] | [[163](#_ENREF_163)] |
| **height** | 10cm | 419 | All | 1.03 (0.92~1.15, 0.642) | 14,160/12,724 | Euro | [[20](#_ENREF_20)] | [[21](#_ENREF_21)] |
|  |  |  | Aggressive | 0.98 (0.84~1.15, 0.822) | 4,446/12,724 | Euro |  |  |
| **height** | NA | 179 | All | 0.99 (0.97~1.01, 0.230) | 20,848/20,214 | Euro | [[18](#_ENREF_18)] | [[161](#_ENREF_161)] |
|  |  |  | Localized PCA | 1.00 (0.98~1.02, 0.720) | 12,975/20,214 | Euro |  |  |
|  |  |  | Advanced PCA | 0.96 (0.93~0.99, 0.010) | 4,325/20,214 | Euro |  |  |
|  |  |  | Low-grade PCA | 0.99 (0.96~1.01, 0.300) | 8,784/20,214 | Euro |  |  |
|  |  |  | High-grade PCA | 1.00 (0.98~1.02, 0.850) | 8,230/20,214 | Euro |  |  |
| **body size** |  |  |  |  |  |  |  |  |
| adulthood body size | NA | **151 (male)** | prostate cancer | 0.89 (0.77~1.02, 0.080) | 79,148/61,106 | Euro | [[34](#_ENREF_34)] | [[35](#_ENREF_35)] |
| early life body size | NA | **66 (male)** | prostate cancer | 0.87 (0.71~1.06, 0.160) | 79,148/61,106 | Euro | [[34](#_ENREF_34)] |  |
| **puberty timing** | NA | 375 (female only ^a^) | prostate cancer | 0.93 (0.88~0.98, 0.004) | 20,219/20,440 | Euro | [[38](#_ENREF_38), [39](#_ENREF_39)] | [[36](#_ENREF_36)] |
| **tanner genital stage in adolescent boys** | NA | 13 (male only) | prostate cancer | 0.95 (0.87~1.04, 0.300) | 1,136/1,791 | Euro | [[38](#_ENREF_38), [164](#_ENREF_164)] | [[165](#_ENREF_165)] |
| **lipid** |  |  |  |  |  |  |  |  |
| HDL | NA | 35 | prostate cancer | 0.99 (0.84~1.17, 0.900) | 22,249/22,133 | Euro | [[23](#_ENREF_23), [25](#_ENREF_25), [166](#_ENREF_166), [167](#_ENREF_167)] | [[168](#_ENREF_168)] |
| LDL | NA | 10 | prostate cancer | 1.24 (0.90~1.69, 0.180) | 22,249/22,133 | Euro |  |  |
| TG | NA | 14 | prostate cancer | 1.09 (0.80~1.50, 0.570) | 22,249/22,133 | Euro |  |  |
| **lipid** |  |  |  |  |  |  |  |  |
| HDL | NA | 41 | prostate cancer | 0.93 (0.74~1.18, 0.542) | N/cases: 10,882/1,322 | Sweden | [[23](#_ENREF_23)] | [[24](#_ENREF_24)] |
| LDL | NA | 32 | prostate cancer | 0.96 (0.78~1.19, 0.705) | N/cases: 10,882/1,322 | Sweden | [[23](#_ENREF_23)] |  |
| TG | NA | 26 | prostate cancer | 0.87 (0.67~1.14, 0.304) | N/cases: 10,882/1,322 | Sweden | [[23](#_ENREF_23)] |  |
| **25(OH)D** | 25nmol/L | 6 | All | 1.00 (0.93~1.07, 0.990) | 79,148/61,106 | Euro | [[45](#_ENREF_45)] | [[46](#_ENREF_46)] |
|  |  |  | Advanced PCA | 1.02 (0.90~1.16, 0.720) | 15,167/58,308 | Euro |  |  |
| **25(OH)D** | 20nmol/L | 5 | prostate cancer | 0.91 (0.80~1.05, 0.190) | 7,532 | Euro | [[44](#_ENREF_44), [51-53](#_ENREF_51)] | [[54](#_ENREF_54)] |
| **25(OH)D** | 25nmol/L | 4 | All | 0.89 (0.77~1.02, 0.080) | 22,898/23,054 | Euro | [[42](#_ENREF_42), [43](#_ENREF_43)] | [[44](#_ENREF_44)] |
|  |  |  | All | 1.08 (0.88~1.33, 0.470) | 14,159/12,712 | Euro |  |  |
|  |  |  | Aggressive | 1.14 (0.85~1.54, 0.380) | 4,445/12,724 | Euro |  |  |
| **smoking initiation** | NA | 378 | prostate cancer | 0.90 (0.80~1.02, 0.104) | 7,872/160,876 | Euro | [[124](#_ENREF_124)] | [[126](#_ENREF_126)] |
|  |  |  | prostate cancer | 0.90 (0.83~0.98, 0.011) | 79,148/61,106 | Euro |  |  |
| **alcohol consumption** | 10g/day | 99 | prostate cancer | 0.91 (0.62~1.34, 0.630) | 7,872/160,876 | Euro | [[124](#_ENREF_124)] | [[126](#_ENREF_126)] |
|  |  |  | prostate cancer | 0.96 (0.74~1.24, 0.753) | 79,148/61,106 | Euro |  |  |
| **milk intake** | 17.1g/day | 1 | prostate cancer | 1.01 (0.97~1.05, 0.718) | 7,872/160,876 | Euro | [[131](#_ENREF_131), [132](#_ENREF_132)] | [[133](#_ENREF_133)] |
|  |  |  | prostate cancer | 1.00 (0.98~1.02, 0.923) | 79,148/61,106 | Euro |  |  |
| **coffee consumption** | per coffee consumption increasing allele | 2 | prostate cancer | 1.01 (0.98~1.03, 0.580) | 22,721/23,034 | Euro | [[134](#_ENREF_134), [169](#_ENREF_169), [170](#_ENREF_170)] | [[171](#_ENREF_171)] |
|  |  |  | High-grade PCA | 1.01 (0.97~1.04, 0.680) | 9,293/9,622 | Euro |  |  |
| **coffee consumption** | per cup/day coffee intake | 35 | prostate cancer | 0.86 (0.75~0.98, 0.027) | 7,532/131,834 | Euro | [[134](#_ENREF_134)] | [[135](#_ENREF_135)] |
| **telomere length** | NA | 9 | All | 1.21 (0.99~1.46, 0.060) | 14,160/12,724 | Euro | [[55](#_ENREF_55), [56](#_ENREF_56), [58](#_ENREF_58)] | [[62](#_ENREF_62)] |
|  |  |  | Aggressive | 1.10 (0.83~1.45, 0.520) | 4,450/12,724 | Euro |  |  |
| **telomere length** | NA | 16 | prostate cancer | 1.12 (0.96~1.30, 0.150) | 22,297/22,323 | Euro | [[55-60](#_ENREF_55)] | [[61](#_ENREF_61)] |
| **telomere length** | NA | 15 | prostate cancer | 1.06 (1.02~1.10, 0.005) | 2,410/307,395 | Euro | [[63](#_ENREF_63)] | [[64](#_ENREF_64)] |
| **telomere length** | NA | 10 | Aggressive PCA | 0.58 (0.36~0.93, 0.021) | 1,889 | Euro Americans | [[55](#_ENREF_55), [58](#_ENREF_58)] | [[172](#_ENREF_172)] |
| **glycemic traits** |  |  |  |  |  |  |  |  |
| fasting glucose | 1mmol/L | **11 (male)** | prostate cancer | 0.93 (0.73~1.17, 0.546) | 79,148/61,106 | Euro | [[71](#_ENREF_71)] | [[72](#_ENREF_72)] |
| HbA1c | 1% | 19 | prostate cancer | 0.90 (0.58~1.40, 0.639) | 79,148/61,106 | Euro |  |  |
| **IGF-1** | NA | 416 | prostate cancer | 1.10 (1.01~1.21, 0.040) | 7,872/160,876 | Euro | [[76](#_ENREF_76)] | [[77](#_ENREF_77)] |
|  |  |  | prostate cancer | 1.03 (0.97~1.09, 0.410) | 79,148/61,106 | Euro |  |  |
| **IGF-1** | 5nmol/L | **122 (male)** | prostate cancer | 1.06 (1.00~1.13, 0.060) | 79,148/61,106 | Euro | NealeLab UK Biobank GWAS Results | [[173](#_ENREF_173)] |
| **C-reactive protein** | NA | 58 | prostate cancer | 1.06 (0.96~1.16, 0.240) | 79,148/61,106 | Euro | [[88](#_ENREF_88)] | [[174](#_ENREF_174)] |
| **tumour necrosis factor** | NA | 4 | prostate cancer | 0.75 (0.51~1.10, 0.138) | 79,194/61,112 | Euro | Inflammatory biomarker genomics: from discovery to causality | [[91](#_ENREF_91)] |
| **arachidonic acid concentrations** | NA | 2 | prostate cancer | 1.02 (0.98~1.06, 0.268) | 7,872/160,876 | Euro | [[92](#_ENREF_92)] | [[93](#_ENREF_93)] |
|  |  |  | prostate cancer | 1.01 (0.97~1.05, 0.793) | 79,148/61,106 | Euro |  |  |
| **metabolite** |  |  |  |  |  |  | [[175](#_ENREF_175)] | [[176](#_ENREF_176)] |
| **Lipoprotein subclass particles** |  |  |  |  |  |  |  |  |
| Concentration of small HDL | NA | 3 | prostate cancer | 1.02 (0.91~1.13, 0.764) | 44,825/27,904 | Euro |  |  |
| **Lipoprotein particle size (mean diameter)** | | |  |  |  |  |  |  |
| VLDL | NA | 9 | prostate cancer | 1.08 (0.99~1.17, 0.061) | 44,825/27,904 | Euro |  |  |
| **Cholesterol** |  |  |  |  |  |  |  |  |
| Cholesterol esters in medium LDL | NA | 14 | prostate cancer | 0.98 (0.93~1.03, 0.363) | 44,825/27,904 | Euro |  |  |
| Free cholesterol in IDL | NA | 12 | prostate cancer | 1.03 (0.97~1.08, 0.339) | 44,825/27,904 | Euro |  |  |
| Free cholesterol in medium HDL | NA | 5 | prostate cancer | 1.00 (0.90~1.13, 0.947) | 44,825/27,904 | Euro |  |  |
| Total cholesterol in medium LDL | NA | 13 | prostate cancer | 0.98 (0.93~1.03, 0.422) | 44,825/27,904 | Euro |  |  |
| Free cholesterol in large LDL | NA | 11 | prostate cancer | 1.00 (0.95~1.06, 0.860) | 44,825/27,904 | Euro |  |  |
| Total cholesterol in small LDL | NA | 10 | prostate cancer | 0.97 (0.91~1.03, 0.256) | 44,825/27,904 | Euro |  |  |
| Total lipids in small HDL | NA | 5 | prostate cancer | 1.02 (0.93~1.12, 0.690) | 44,825/27,904 | Euro |  |  |
| Phospholipids in IDL | NA | 12 | prostate cancer | 1.02 (0.96~1.07, 0.572) | 44,825/27,904 | Euro |  |  |
| Phospholipids in very small VLDL | NA | 12 | prostate cancer | 1.02 (0.96~1.09, 0.501) | 44,825/27,904 | Euro |  |  |
| **Amino Acids** |  |  |  |  |  |  |  |  |
| Leucine | NA | 3 | prostate cancer | 1.12 (0.96~1.31, 0.148) | 44,825/27,904 | Euro |  |  |
| Tyrosine | NA | 3 | prostate cancer | 0.92 (0.79~1.07, 0.278) | 44,825/27,904 | Euro |  |  |
| Valine | NA | 4 | prostate cancer | 1.03 (0.90~1.18, 0.650) | 44,825/27,904 | Euro |  |  |
| **metabolite** |  |  |  |  |  |  |  |  |
| Acetate | NA | 1 | prostate cancer | 0.89 (0.63~1.25, 0.501) | 44,825/27,904 | Euro | [[175](#_ENREF_175)] | [[177](#_ENREF_177)] |
| Pyruvate | NA | 2 | prostate cancer | 1.29 (1.03~1.62, 0.027) | 44,825/27,904 | Euro |  |  |
| Valine | NA | 4 | prostate cancer | 1.03 (0.90~1.18, 0.647) | 44,825/27,904 | Euro |  |  |
| DHA | NA | 4 | prostate cancer | 0.97 (0.85~1.01, 0.647) | 44,825/27,904 | Euro |  |  |
| Glycine | NA | 6 | prostate cancer | 0.99 (0.92~1.06, 0.787) | 44,825/27,904 | Euro |  |  |
| **skin color score** | NA | 6 | prostate cancer | 1.06 (0.99~1.12, 0.080) | 1,136/1,791 | Euro | [[178](#_ENREF_178)] | [[179](#_ENREF_179)] |
| **tanning score** | NA | 13 | prostate cancer | 1.05 (1.02~1.09, 0.004) | 1,136/1,791 | Euro |  |  |
| **freckling score** | NA | 8 | prostate cancer | 1.03 (0.99~1.08, 0.170) | 1,136/1,791 | Euro |  |  |
| **pigmentation phenotypes** | NA | 17 |  |  |  |  | [[99-111](#_ENREF_99)] | [[112](#_ENREF_112)] |
| **Hair color** |  |  |  |  |  |  |  |  |
| dark hair |  |  | prostate cancer | - | 919/1,067 | Spanish |  |  |
| Light brown hair |  |  | prostate cancer | 1.29 (0.90~1.85, 0.158) | 83/99 | Spanish |  |  |
| blonde hair |  |  | prostate cancer | 1.12 (0.79~1.57, 0.530) | 104/113 | Spanish |  |  |
| **Skin color** |  |  |  |  |  |  |  |  |
| Dark skin |  |  | prostate cancer | - | 263/306 | Spanish |  |  |
| Light brown skin |  |  | prostate cancer | 0.89 (0.72~1.12, 0.332) | 386/542 | Spanish |  |  |
| Fair skin |  |  | prostate cancer | 1.13 (0.88~1.46, 0.341) | 458/604 | Spanish |  |  |
| **Eye color** |  |  |  |  |  |  |  |  |
| Black/dark brown |  |  | prostate cancer | - | 623/742 | Spanish |  |  |
| Light brown/green |  |  | prostate cancer | 0.79 (0.64~0.99, 0.040) | 325/473 | Spanish |  |  |
| Blue/grey |  |  | prostate cancer | 0.91 (0.69~1.21, 0.533) | 161/238 | Spanish |  |  |
| **Freckles** |  |  |  |  |  |  |  |  |
| No |  |  | prostate cancer | - | 185/297 | Spanish |  |  |
| Yes |  |  | prostate cancer | 1.27 (0.98~1.64, 0.070) | 919/1,153 | Spanish |  |  |
| **birthweight** | NA | 7 | All | 1.33 (0.96~1.82, 0.082) | 14,160/12,724 | Euro | [[30](#_ENREF_30)] | [[8](#_ENREF_8)] |
|  |  |  | Aggressive | 1.63 (1.03~2.57, 0.037) | 4,450/12,724 | Euro |  |  |
| **waist-hip ratio (WHR)** | NA | 14 | All | 1.02 (0.72~1.46, 0.900) | 14,160/12,724 | Euro | [[33](#_ENREF_33)] | [[8](#_ENREF_8)] |
|  |  |  | Aggressive | 1.19 (0.71~1.98, 0.510) | 4,450/12,724 | Euro |  |  |
| **testosterone** |  |  |  |  |  |  |  |  |
| **bioavailable testosterone** | 3.7nmol/L | **123 (male)** | prostate cancer | 1.23 (1.13~1.33, 3.70E-07) | 67,158/48,350 | Euro | [[118-121](#_ENREF_118)] | [[122](#_ENREF_122)] |
| **total testosterone** | 3.7nmol/L | **225 (male)** | prostate cancer | 1.02 (0.95~1.09, 0.660) | 67,158/48,350 | Euro | [[118-121](#_ENREF_118)] |  |
| **calculated free testosterone** | 0.1nmol/L | **93 (male)** | prostate cancer | 1.51 (1.21~1.88, 2.10E-04) | 7,586/149,624 | Euro | [[180](#_ENREF_180)] | [[181](#_ENREF_181)] |
| **selenium** | 114μg/L | 11 | All | 1.01 (0.89~1.13, 0.930) | 44,825/27,904 | Euro | [[182](#_ENREF_182), [183](#_ENREF_183)] | [[184](#_ENREF_184)] |
|  |  |  | High-grade/Advanced PCA | 1.21 (0.98~1.49, 0.070) | 44,825/27,904 | Euro |  |  |
| **Microseminoprotein-beta (MSP)** | 1ng/ml | 1 | prostate cancer | 0.96 (0.95~0.97, 1.58E-14) | 1,871/1,871 | Euro | [[185](#_ENREF_185)] | [[186](#_ENREF_186)] |
| **serum iron levels** | 1μmol/L | 5 | prostate cancer | 0.92 (0.86~0.98, 0.007) | 79,148/61,106 | Euro | [[79](#_ENREF_79)] | [[163](#_ENREF_163)] |
| **serum calcium** | 0.5mg/dL | 5 | All | 0.83 (0.63~1.08, 0.120) | 44,825/27,904 | Euro | [[187](#_ENREF_187)] | [[188](#_ENREF_188)] |
|  |  |  | Aggressive | 0.98 (0.57~1.70, 0.930) | 6,263/27,235 | Euro |  |  |
| **Thyroid dysfunction** | NA | 58 | prostate cancer | 0.91 (0.84~0.99, 0.026) | 7,872 | Euro | [[94](#_ENREF_94)] | [[95](#_ENREF_95)] |
| TSH | NA | 7 | prostate cancer | 0.96 (0.89~1.04, 0.305) | 7,872 | Euro |  |  |
| Hypothyroidism | NA | 8 | prostate cancer | 0.99 (0.93~1.06, 0.820) | 7,872 | Euro |  |  |
| Hyperthyroidism | NA | 31 | prostate cancer | 1.06 (0.93~1.20, 0.400) | 7,872 | Euro |  |  |
| Free thyroxine | NA | 58 | prostate cancer | 0.91 (0.84~0.99, 0.026) | 7,872 | Euro |  |  |
| **IDO1** | NA | 3 | prostate cancer | 0.96 (0.93~0.99, 0.010) | 79,148/61,106 | Euro | [[87](#_ENREF_87)] | [[96](#_ENREF_96)] |
| **KAT3** | NA | 2 | prostate cancer | 0.98 (0.92~1.03, 0.420) | 79,148/61,106 | Euro | [[87](#_ENREF_87)] |  |
| **IGFBP-3** | NA | 4 | All | 1.12 (0.91~1.36, 0.180) | 44,825/27,904 | Euro | [[78](#_ENREF_78)] | [[189](#_ENREF_189)] |
|  |  |  | Advanced PCA | 1.16 (0.87~1.55, 0.190) | 6,263/27,235 | Euro |  |  |
| **Vitamin D** | NA | 8 | prostate cancer | 1.00 (0.99~1.00, 0.370) | 337,159(total) | Euro | [[40](#_ENREF_40)] | [[41](#_ENREF_41)] |
| **vitamin B (12)** | NA | 2 | prostate cancer | 0.60 (0.16~2.15, 0.400) | 913/895 | Euro | [[190](#_ENREF_190), [191](#_ENREF_191)] | [[192](#_ENREF_192)] |
| **total transcobalamin** | NA | 1 | prostate cancer | 0.41 (0.13~1.32, 0.100) | 913/895 | Euro | [[193](#_ENREF_193)] |  |
| **circulating monounsaturated fat** | 1mmol/L | 5 | prostate cancer | 1.11 (1.02~1.20, 0.020) | 79,148/61,106 | Euro | [[175](#_ENREF_175)] | [[163](#_ENREF_163)] |
| **polyunsaturated fatty acid (PUFA)** |  |  |  |  |  |  |  |  |
| **ω-6 PUFAs** |  |  |  |  |  |  |  |  |
| Linoleic acid (LA) | NA | 4 | All | 1.00 (0.98~1.02, 0.650) | 22,721/23,034 | Euro | [[92](#_ENREF_92)] | [[194](#_ENREF_194)] |
|  |  |  | Smoking status |  |  |  |  |  |
|  |  |  | Ever smokers | 0.99 (0.95~1.04, 0.810) | 4,789/4,914 | Euro |  |  |
|  |  |  | Never smokers | 1.00 (0.95~1.06, 0.980) | 3,091/2,954 | Euro |  |  |
|  |  |  | Age |  |  |  |  |  |
|  |  |  | <62 years of age | 0.95 (0.92~0.98, 0.026) | 8,259/13,684 | Euro |  |  |
|  |  |  | ≥62 years of age | 1.04 (1.01~1.07, 0.006) | 14,462/9,350 | Euro |  |  |
|  |  |  | Disease severity |  |  |  |  |  |
|  |  |  | Advanced PCA | 0.99 (0.97~1.01, 0.240) | 4,802/23,034 | Euro |  |  |
|  |  |  | Non-advanced PCA | 1.02 (0.99~1.05, 0.310) | 17,919/23,034 | Euro |  |  |
|  |  |  | Method of detection |  |  |  |  |  |
|  |  |  | Screen-detected | 0.98 (0.94~1.02, 0.250) | 4,414/23,034 | Euro |  |  |
|  |  |  | Clinically detected | 1.00 (0.97~1.03, 0.860) | 8,597/23,034 | Euro |  |  |
| Arachidonic acid (AA) | NA | 2 | All | 1.01 (0.99~1.03, 0.360) | 22,721/23,034 | Euro | [[92](#_ENREF_92)] |  |
|  |  |  | Smoking status |  |  |  |  |  |
|  |  |  | Ever smokers | 1.01 (0.97~1.05, 0.690) | 4,789/4,914 | Euro |  |  |
|  |  |  | Never smokers | 1.01 (0.95~1.07, 0.760) | 3,091/2,954 | Euro |  |  |
|  |  |  | Age |  |  |  |  |  |
|  |  |  | <62 years of age | 1.05 (1.02~1.08, 0.002) | 8,259/13,684 | Euro |  |  |
|  |  |  | ≥62 years of age | 0.98 (0.95~1.01, 0.110) | 14,462/9,350 | Euro |  |  |
|  |  |  | Disease severity |  |  |  |  |  |
|  |  |  | Advanced PCA | 1.01 (0.99~1.04, 0.580) | 4,802/23,034 | Euro |  |  |
|  |  |  | Non-advanced PCA | 0.99 (0.96~1.02, 0.190) | 17,919/23,034 | Euro |  |  |
|  |  |  | Method of detection |  |  |  |  |  |
|  |  |  | Screen-detected | 1.04 (0.99~1.08, 0.060) | 4,414/23,034 | Euro |  |  |
|  |  |  | Clinically detected | 1.00 (0.97~1.03, 0.920) | 8,597/23,034 | Euro |  |  |
| **ω-3 PUFAs** |  |  |  |  |  |  |  |  |
| α-Linolenic acid (ALA) | NA | 1 | All | 0.99 (0.97~1.01, 0.380) | 22,721/23,034 | Euro | [[195](#_ENREF_195)] |  |
|  |  |  | Smoking status |  |  |  |  |  |
|  |  |  | Ever smokers | 0.99 (0.95~1.03, 0.660) | 4,789/4,914 | Euro |  |  |
|  |  |  | Never smokers | 0.99 (0.94~1.05, 0.810) | 3,091/2,954 | Euro |  |  |
|  |  |  | Age |  |  |  |  |  |
|  |  |  | <62 years of age | 0.96 (0.93~0.98, 0.002) | 8,259/13,684 | Euro |  |  |
|  |  |  | ≥62 years of age | 1.02 (0.99~1.05, 0.100) | 14,462/9,350 | Euro |  |  |
|  |  |  | Disease severity |  |  |  |  |  |
|  |  |  | Advanced PCA | 0.99 (0.97~1.01, 0.590) | 4,802/23,034 | Euro |  |  |
|  |  |  | Non-advanced PCA | 1.01 (0.98~1.04, 0.200) | 17,919/23,034 | Euro |  |  |
|  |  |  | Method of detection |  |  |  |  |  |
|  |  |  | Screen-detected | 0.97 (0.93~1.00, 0.070) | 4,414/23,034 | Euro |  |  |
|  |  |  | Clinically detected | 1.00 (0.97~1.03, 0.980) | 8,597/23,034 | Euro |  |  |
| Eicosapentaenoic acid (EPA) | NA | 2 | All | 1.01 (0.99~1.03, 0.310) | 22,721/23,034 | Euro | [[195](#_ENREF_195)] |  |
|  |  |  | Smoking status |  |  |  |  |  |
|  |  |  | Ever smokers | 1.01 (0.97~1.06, 0.650) | 4,789/4,914 | Euro |  |  |
|  |  |  | Never smokers | 0.99 (0.93~1.04, 0.620) | 3,091/2,954 | Euro |  |  |
|  |  |  | Age |  |  |  |  |  |
|  |  |  | <62 years of age | 1.04 (1.01~1.06, 0.017) | 8,259/13,684 | Euro |  |  |
|  |  |  | ≥62 years of age | 0.99 (0.96~1.02, 0.430) | 14,462/9,350 | Euro |  |  |
|  |  |  | Disease severity |  |  |  |  |  |
|  |  |  | Advanced PCA | 1.01 (0.99~1.03, 0.960) | 4,802/23,034 | Euro |  |  |
|  |  |  | Non-advanced PCA | 1.00 (0.97~1.03, 0.230) | 17,919/23,034 | Euro |  |  |
|  |  |  | Method of detection |  |  |  |  |  |
|  |  |  | Screen-detected | 1.03 (0.99~1.07, 0.080) | 4,414/23,034 | Euro |  |  |
|  |  |  | Clinically detected | 0.99 (0.96~1.02, 0.580) | 8,597/23,034 | Euro |  |  |
| Docosapentaenoic acid (DPA) | NA | 3 | All | 1.01 (0.99~1.03, 0.160) | 22,721/23,034 | Euro | [[195](#_ENREF_195)] |  |
|  |  |  | Smoking status |  |  |  |  |  |
|  |  |  | Ever smokers | 1.01 (0.97~1.05, 0.660) | 4,789/4,914 | Euro |  |  |
|  |  |  | Never smokers | 1.00 (0.94~1.05, 0.940) | 3,091/2,954 | Euro |  |  |
|  |  |  | Age |  |  |  |  |  |
|  |  |  | <62 years of age | 1.05 (1.02~1.08, 0.002) | 8,259/13,684 | Euro |  |  |
|  |  |  | ≥62 years of age | 0.99 (0.96~1.01, 0.280) | 14,462/9,350 | Euro |  |  |
|  |  |  | Disease severity |  |  |  |  |  |
|  |  |  | Advanced PCA | 1.02 (0.99~1.03, 0.690) | 4,802/23,034 | Euro |  |  |
|  |  |  | Non-advanced PCA | 1.01 (0.97~1.04, 0.130) | 17,919/23,034 | Euro |  |  |
|  |  |  | Method of detection |  |  |  |  |  |
|  |  |  | Screen-detected | 1.03 (0.99~1.07, 0.080) | 4,414/23,034 | Euro |  |  |
|  |  |  | Clinically detected | 1.00 (0.97~1.03, 0.970) | 8,597/23,034 | Euro |  |  |
| Docosahexaenoic acid (DHA) | NA | 1 | All | 1.00 (0.98~1.02, 0.810) | 22,721/23,034 | Euro | [[195](#_ENREF_195)] |  |
|  |  |  | Smoking status |  |  |  |  |  |
|  |  |  | Ever smokers | 1.00 (0.96~1.04, 0.990) | 4,789/4,914 | Euro |  |  |
|  |  |  | Never smokers | 0.98 (0.93~1.04, 0.560) | 3,091/2,954 | Euro |  |  |
|  |  |  | Age |  |  |  |  |  |
|  |  |  | <62 years of age | 1.01 (0.98~1.04, 0.430) | 8,259/13,684 | Euro |  |  |
|  |  |  | ≥62 years of age | 0.99 (0.96~1.02, 0.480) | 14,462/9,350 | Euro |  |  |
|  |  |  | Disease severity |  |  |  |  |  |
|  |  |  | Advanced PCA | 1.00 (0.98~1.02, 0.430) | 4,802/23,034 | Euro |  |  |
|  |  |  | Non-advanced PCA | 1.01 (0.98~1.05, 0.990) | 17,919/23,034 | Euro |  |  |
|  |  |  | Method of detection |  |  |  |  |  |
|  |  |  | Screen-detected | 1.00 (0.96~1.04, 0.930) | 4,414/23,034 | Euro |  |  |
|  |  |  | Clinically detected | 0.99 (0.96~1.02, 0.520) | 8,597/23,034 | Euro |  |  |
| **type 2 diabetes** | NA | 112 | prostate cancer | 1.02 (0.97~1.07, 0.429) | 79,148/61,106 | Euro | [[147](#_ENREF_147)] | [[72](#_ENREF_72)] |
| **type 2 diabetes** | NA | 399 | prostate cancer | 0.97 (0.93~1.01, 0.108) | 7,872/359,771 | Euro | [[147](#_ENREF_147)] | [[148](#_ENREF_148)] |
| **major depressive disorder** | NA | 43 | prostate cancer | 1.12 (0.97~1.30, 0.135) | 79,148/61,106 | Euro | [[196](#_ENREF_196)] | [[197](#_ENREF_197)] |
| **vitiligo** | NA | 37 | prostate cancer | 1.01 (0.99~1.03, 0.313) | 79,148/61,106 | Euro | [[156](#_ENREF_156), [157](#_ENREF_157)] | [[158](#_ENREF_158)] |
| **allergic disease** | NA | 132 | All | 1.00 (0.94~1.05, 0.930) | 79,148/61,106 | Euro | [[142](#_ENREF_142), [143](#_ENREF_143)] | [[144](#_ENREF_144)] |
|  |  |  | Advanced PCA | 0.97 (0.90~1.05, 0.460) | 15,167/58,308 | Euro |  |  |
| **physical activity** | 8.14milli-gravities | 2 | prostate cancer | 0.49 (0.33~0.72, 3.00E-04) | 79,148/61,106 | Euro | [[198](#_ENREF_198)] | [[163](#_ENREF_163)] |

^a^ Strong sharing exist between the genetic architectures of age at menarche in women and age at voice breaking in men (genetic correlation=0.75, *P*=1.20E-79) [[36](#_ENREF_36)].

MR, Mendelian randomization; BMI, body mass index; HDL, high density lipoprotein; LDL, low density lipoprotein; TG, triglycerides; IGF-1, insulin-like growth factor-1; VLDL, very low-density lipoprotein; TSH, thyroid-stimulating hormone level; PCA, prostate cancer; SD, standard deviation; OR, odds ratio; CI, confidence internal; *P*, *P* value; *N*, sample size; Euro, European; IV, instrumental variable; Ref, reference.

**Supplementary Table 3** Summary information of instruments for anthropometric traits obtained from the GIANT consortium [[1](#_ENREF_1), [199](#_ENREF_199)] and the heterogeneity test between females and males

| Trait | SNP | CHR | POS | *GENE* | A1/A2 | sex-combined | | | |  | female-specific | | | |  | male-specific | | | | Q | *P*_het_ | I^2^ |
| --- | --- | --- | --- | --- | --- | --- | --- | --- | --- | --- | --- | --- | --- | --- | --- | --- | --- | --- | --- | --- | --- | --- |
|  |  |  |  |  |  | BETA | SE | *P* | *N* |  | BETA | SE | *P* | *N* |  | BETA | SE | *P* | *N* |  |  |  |
| BMI | rs11165643 | 1 | 96,924,097 | *PTBP2* | T/C | 0.022 | 0.003 | **2.07E-12** | 320,730 |  | 0.023 | 0.004 | **5.76E-09** | 171,316 |  | 0.021 | 0.004 | 4.30E-07 | 152,125 | 0.161 | 0.688 | 0.0% |
| BMI | rs11583200 | 1 | 50,559,820 | *ELAVL4* | C/T | 0.018 | 0.003 | **1.48E-08** | 322,095 |  | 0.018 | 0.004 | 1.70E-05 | 171,938 |  | 0.017 | 0.004 | 2.89E-05 | 152,865 | 0.000 | 0.986 | 0.0% |
| BMI | rs12401738 | 1 | 78,446,761 | *FUBP1* | A/G | 0.021 | 0.003 | **1.15E-10** | 322,070 |  | 0.026 | 0.004 | **6.09E-10** | 171,933 |  | 0.016 | 0.004 | 1.80E-04 | 152,848 | 2.611 | 0.106 | 61.7% |
| BMI | rs12566985 | 1 | 75,002,193 | *FPGT-TNNI3K* | G/A | 0.024 | 0.003 | **3.28E-15** | 319,282 |  | 0.027 | 0.004 | **1.05E-11** | 170,990 |  | 0.022 | 0.004 | 1.38E-07 | 151,004 | 0.889 | 0.346 | 0.0% |
| BMI | rs17024393 | 1 | 110,154,688 | *GNAT2* | C/T | 0.066 | 0.009 | **7.03E-14** | 297,874 |  | 0.071 | 0.011 | **3.53E-10** | 158,336 |  | 0.061 | 0.012 | 1.61E-07 | 141,994 | 0.441 | 0.507 | 0.0% |
| BMI | rs2820292 | 1 | 201,784,287 | *NAV1* | C/A | 0.020 | 0.003 | **1.83E-10** | 321,707 |  | 0.018 | 0.004 | 4.89E-06 | 171,758 |  | 0.023 | 0.004 | **2.26E-08** | 152,659 | 0.649 | 0.421 | 0.0% |
| BMI | rs3101336 | 1 | 72,751,185 | *NEGR1* | C/T | 0.033 | 0.003 | **2.66E-26** | 316,872 |  | 0.033 | 0.004 | **8.91E-17** | 168,920 |  | 0.035 | 0.004 | **1.73E-16** | 150,657 | 0.043 | 0.836 | 0.0% |
| BMI | rs543874 | 1 | 177,889,480 | *SEC16B* | G/A | 0.048 | 0.004 | **2.62E-35** | 322,008 |  | 0.060 | 0.005 | **9.61E-34** | 171,901 |  | 0.034 | 0.005 | **2.73E-11** | 152,818 | 13.457 | **2.44E-04** | 92.6% |
| BMI | rs657452 | 1 | 49,589,847 | *AGBL4* | A/G | 0.023 | 0.003 | **5.48E-13** | 313,651 |  | 0.023 | 0.004 | **1.70E-08** | 167,600 |  | 0.022 | 0.004 | 2.53E-07 | 148,754 | 0.084 | 0.772 | 0.0% |
| BMI | rs977747 | 1 | 47,684,677 | *TAL1* | T/G | 0.017 | 0.003 | **2.18E-08** | 322,086 |  | 0.017 | 0.004 | 1.11E-05 | 171,949 |  | 0.016 | 0.004 | 9.85E-05 | 152,847 | 0.060 | 0.807 | 0.0% |
| BMI | rs1016287 | 2 | 59,305,625 | *FLJ30838* | T/C | 0.023 | 0.003 | **2.25E-11** | 321,969 |  | 0.025 | 0.004 | **5.87E-09** | 171,885 |  | 0.020 | 0.004 | 4.04E-06 | 152,795 | 0.646 | 0.422 | 0.0% |
| BMI | rs10182181 | 2 | 25,150,296 | *ADCY3* | G/A | 0.031 | 0.003 | **8.78E-24** | 321,759 |  | 0.037 | 0.004 | **3.91E-21** | 171,763 |  | 0.023 | 0.004 | **6.03E-09** | 152,701 | 5.583 | 0.018 | 82.1% |
| BMI | rs11126666 | 2 | 26,928,811 | *KCNK3* | A/G | 0.021 | 0.003 | **1.33E-09** | 321,979 |  | 0.016 | 0.004 | 2.90E-04 | 171,890 |  | 0.026 | 0.005 | **1.29E-08** | 152,799 | 2.425 | 0.119 | 58.8% |
| BMI | rs11688816 | 2 | 63,053,048 | *EHBP1* | G/A | 0.017 | 0.003 | **1.89E-08** | 322,051 |  | 0.019 | 0.004 | 9.60E-07 | 171,916 |  | 0.015 | 0.004 | 3.84E-04 | 152,841 | 0.632 | 0.426 | 0.0% |
| BMI | rs13021737 | 2 | 632,348 | *TMEM18* | G/A | 0.060 | 0.004 | **1.11E-50** | 318,287 |  | 0.069 | 0.005 | **6.99E-40** | 168,674 |  | 0.052 | 0.005 | **3.86E-22** | 152,324 | 4.903 | 0.027 | 79.6% |
| BMI | rs1460676 | 2 | 164,567,689 | *FIGN* | C/T | 0.021 | 0.004 | **4.98E-08** | 322,089 |  | 0.020 | 0.005 | 1.20E-04 | 171,949 |  | 0.019 | 0.005 | 3.85E-04 | 152,850 | 0.014 | 0.904 | 0.0% |
| BMI | rs1528435 | 2 | 181,550,962 | *UBE2E3* | T/C | 0.018 | 0.003 | **1.20E-08** | 321,924 |  | 0.014 | 0.004 | 8.40E-04 | 171,860 |  | 0.023 | 0.004 | **4.32E-08** | 152,775 | 2.457 | 0.117 | 59.3% |
| BMI | rs17203016 | 2 | 208,255,518 | *CREB1* | G/A | 0.021 | 0.004 | **3.41E-08** | 316,466 |  | 0.019 | 0.005 | 1.49E-04 | 168,734 |  | 0.023 | 0.005 | 5.87E-06 | 150,443 | 0.355 | 0.551 | 0.0% |
| BMI | rs2121279 | 2 | 143,043,285 | *LRP1B* | T/C | 0.025 | 0.004 | **2.31E-08** | 322,065 |  | 0.021 | 0.006 | 1.74E-04 | 171,938 |  | 0.029 | 0.006 | 4.30E-07 | 152,837 | 1.002 | 0.317 | 0.2% |
| BMI | rs2176040 | 2 | 227,092,802 | *LOC646736* | A/G | 0.024 | 0.004 | **9.99E-09** | 321,972 |  | 0.006 | 0.004 | 0.119 | 171,865 |  | 0.024 | 0.004 | **9.99E-09** | 152,818 | 8.890 | 0.003 | 88.8% |
| BMI | rs492400 | 2 | 219,349,752 | *USP37* | C/T | 0.024 | 0.004 | **6.78E-09** | 321,090 |  | 0.008 | 0.004 | 0.038 | 171,648 |  | 0.024 | 0.004 | **6.78E-09** | 152,153 | 7.417 | 0.006 | 86.5% |
| BMI | rs7599312 | 2 | 213,413,231 | *ERBB4* | G/A | 0.022 | 0.003 | **1.17E-10** | 322,024 |  | 0.018 | 0.004 | 2.69E-05 | 171,909 |  | 0.026 | 0.005 | **8.68E-09** | 152,819 | 1.345 | 0.246 | 25.7% |
| BMI | rs13078960 | 3 | 85,807,590 | *CADM2* | G/T | 0.030 | 0.004 | **1.74E-14** | 322,135 |  | 0.034 | 0.005 | **1.41E-11** | 171,963 |  | 0.026 | 0.005 | 4.28E-07 | 152,882 | 1.193 | 0.275 | 16.2% |
| BMI | rs1516725 | 3 | 185,824,004 | *ETV5* | C/T | 0.045 | 0.005 | **1.89E-22** | 320,644 |  | 0.047 | 0.006 | **1.91E-15** | 171,049 |  | 0.044 | 0.006 | **1.05E-12** | 152,306 | 0.100 | 0.752 | 0.0% |
| BMI | rs16851483 | 3 | 141,275,436 | *RASA2* | T/G | 0.048 | 0.008 | **3.55E-10** | 233,929 |  | 0.052 | 0.010 | **4.81E-08** | 132,027 |  | 0.043 | 0.010 | 2.62E-05 | 104,613 | 0.418 | 0.518 | 0.0% |
| BMI | rs2365389 | 3 | 61,236,462 | *FHIT* | C/T | 0.020 | 0.003 | **1.63E-10** | 316,768 |  | 0.020 | 0.004 | 2.98E-07 | 167,969 |  | 0.019 | 0.004 | 2.84E-06 | 151,511 | 0.030 | 0.861 | 0.0% |
| BMI | rs3849570 | 3 | 81,792,112 | *GBE1* | A/C | 0.019 | 0.003 | **2.60E-08** | 284,339 |  | 0.015 | 0.004 | 9.47E-04 | 153,514 |  | 0.023 | 0.005 | 2.49E-07 | 133,536 | 1.867 | 0.172 | 46.4% |
| BMI | rs6804842 | 3 | 25,106,437 | *RARB* | G/A | 0.019 | 0.003 | **2.48E-09** | 321,463 |  | 0.017 | 0.004 | 1.63E-05 | 171,628 |  | 0.020 | 0.004 | 7.69E-07 | 152,546 | 0.312 | 0.576 | 0.0% |
| BMI | rs10938397 | 4 | 45,182,527 | *GNPDA2* | G/A | 0.040 | 0.003 | **3.21E-38** | 320,955 |  | 0.040 | 0.004 | **2.98E-23** | 171,386 |  | 0.040 | 0.004 | **2.08E-22** | 152,279 | 0.001 | 0.972 | 0.0% |
| BMI | rs11727676 | 4 | 145,659,064 | *HHIP* | T/C | 0.036 | 0.006 | **2.55E-08** | 296,401 |  | 0.031 | 0.008 | 1.82E-04 | 159,416 |  | 0.040 | 0.008 | 1.49E-06 | 139,695 | 0.594 | 0.441 | 0.0% |
| BMI | rs13107325 | 4 | 103,188,709 | *SLC39A8* | T/C | 0.048 | 0.007 | **1.83E-12** | 321,461 |  | 0.045 | 0.009 | 2.90E-07 | 171,446 |  | 0.053 | 0.009 | **2.72E-09** | 152,725 | 0.408 | 0.523 | 0.0% |
| BMI | rs17001654 | 4 | 77,129,568 | *SCARB2* | G/C | 0.031 | 0.005 | **7.76E-09** | 233,722 |  | 0.032 | 0.007 | 1.54E-06 | 131,962 |  | 0.028 | 0.007 | 1.13E-04 | 104,471 | 0.200 | 0.655 | 0.0% |
| BMI | rs2112347 | 5 | 75,015,242 | *POC5* | T/G | 0.026 | 0.003 | **6.19E-17** | 322,019 |  | 0.030 | 0.004 | **3.15E-13** | 171,899 |  | 0.021 | 0.004 | 5.47E-07 | 152,831 | 2.299 | 0.129 | 56.5% |
| BMI | rs7715256 | 5 | 153,537,893 | *GALNT10* | G/T | 0.017 | 0.003 | **8.85E-09** | 322,084 |  | 0.018 | 0.004 | 5.82E-06 | 171,943 |  | 0.015 | 0.004 | 2.40E-04 | 152,852 | 0.239 | 0.625 | 0.0% |
| BMI | rs13191362 | 6 | 163,033,350 | *PARK2* | A/G | 0.028 | 0.005 | **7.34E-09** | 321,902 |  | 0.030 | 0.006 | 1.15E-06 | 171,843 |  | 0.025 | 0.006 | 1.12E-04 | 152,770 | 0.387 | 0.534 | 0.0% |
| BMI | rs13201877 | 6 | 137,675,541 | *IFNGR1* | G/A | 0.024 | 0.004 | **4.29E-08** | 322,095 |  | 0.019 | 0.006 | 8.08E-04 | 171,945 |  | 0.028 | 0.006 | 1.56E-06 | 152,861 | 1.204 | 0.273 | 16.9% |
| BMI | rs2033529 | 6 | 40,348,653 | *TDRG1* | G/A | 0.019 | 0.003 | **1.39E-08** | 321,917 |  | 0.020 | 0.004 | 2.83E-06 | 171,816 |  | 0.018 | 0.004 | 4.64E-05 | 152,800 | 0.140 | 0.709 | 0.0% |
| BMI | rs205262 | 6 | 34,563,164 | *C6orf106* | G/A | 0.022 | 0.004 | **1.75E-10** | 315,542 |  | 0.027 | 0.005 | **2.04E-09** | 168,103 |  | 0.017 | 0.005 | 2.89E-04 | 150,148 | 2.512 | 0.113 | 60.2% |
| BMI | rs2207139 | 6 | 50,845,490 | *TFAP2B* | G/A | 0.045 | 0.004 | **4.13E-29** | 322,019 |  | 0.047 | 0.005 | **2.40E-19** | 171,904 |  | 0.044 | 0.005 | **1.62E-16** | 152,826 | 0.113 | 0.736 | 0.0% |
| BMI | rs9374842 | 6 | 120,185,665 | *LOC285762* | T/C | 0.023 | 0.004 | **2.67E-08** | 322,008 |  | 0.018 | 0.005 | 8.54E-05 | 171,903 |  | 0.019 | 0.005 | 3.03E-05 | 152,815 | 0.068 | 0.794 | 0.0% |
| BMI | rs9400239 | 6 | 108,977,663 | *FOXO3* | C/T | 0.019 | 0.003 | **1.61E-08** | 321,988 |  | 0.023 | 0.004 | 1.29E-07 | 171,900 |  | 0.015 | 0.004 | 5.36E-04 | 152,798 | 1.521 | 0.217 | 34.3% |
| BMI | rs1167827 | 7 | 75,163,169 | *HIP1* | G/A | 0.020 | 0.003 | **6.33E-10** | 306,238 |  | 0.017 | 0.004 | 3.69E-05 | 166,441 |  | 0.023 | 0.004 | 8.80E-08 | 142,506 | 1.039 | 0.308 | 3.7% |
| BMI | rs2245368 | 7 | 76,608,143 | *PMS2L11* | C/T | 0.032 | 0.006 | **3.19E-08** | 205,675 |  | 0.028 | 0.008 | 3.11E-04 | 97,727 |  | 0.034 | 0.007 | 1.48E-06 | 107,956 | 0.357 | 0.550 | 0.0% |
| BMI | rs6465468 | 7 | 95,169,514 | *ASB4* | T/G | 0.025 | 0.005 | **4.98E-08** | 307,937 |  | 0.025 | 0.005 | **4.98E-08** | 166,136 |  | 0.007 | 0.005 | 0.126 | 144,512 | 6.834 | 0.009 | 85.4% |
| BMI | rs9641123 | 7 | 93,197,732 | *CALCR* | C/G | 0.029 | 0.005 | **2.08E-10** | 233,515 |  | 0.025 | 0.005 | 2.43E-07 | 131,826 |  | 0.011 | 0.005 | 0.028 | 104,401 | 3.785 | 0.052 | 73.6% |
| BMI | rs16907751 | 8 | 81,375,457 | *ZBTB10* | C/T | 0.047 | 0.009 | **3.89E-08** | 307,752 |  | 0.022 | 0.009 | 0.011 | 164,621 |  | 0.047 | 0.009 | **3.89E-08** | 145,842 | 4.277 | 0.039 | 76.6% |
| BMI | rs17405819 | 8 | 76,806,584 | *HNF4G* | T/C | 0.022 | 0.003 | **2.07E-11** | 322,085 |  | 0.024 | 0.004 | **1.45E-08** | 171,944 |  | 0.021 | 0.004 | 1.93E-06 | 152,852 | 0.324 | 0.569 | 0.0% |
| BMI | rs2033732 | 8 | 85,079,709 | *RALYL* | C/T | 0.019 | 0.004 | **4.89E-08** | 321,406 |  | 0.019 | 0.005 | 2.08E-05 | 171,606 |  | 0.019 | 0.005 | 7.13E-05 | 152,511 | 0.009 | 0.927 | 0.0% |
| BMI | rs10733682 | 9 | 129,460,914 | *LMX1B* | A/G | 0.017 | 0.003 | **1.83E-08** | 320,727 |  | 0.023 | 0.004 | **1.67E-08** | 171,261 |  | 0.011 | 0.004 | 0.006 | 152,175 | 3.934 | 0.047 | 74.6% |
| BMI | rs10968576 | 9 | 28,414,339 | *LINGO2* | G/A | 0.025 | 0.003 | **6.61E-14** | 322,061 |  | 0.029 | 0.004 | **1.04E-11** | 171,929 |  | 0.020 | 0.004 | 5.11E-06 | 152,842 | 2.289 | 0.130 | 56.3% |
| BMI | rs1928295 | 9 | 120,378,483 | *TLR4* | T/C | 0.019 | 0.003 | **7.91E-10** | 321,979 |  | 0.026 | 0.004 | **3.42E-11** | 171,877 |  | 0.012 | 0.004 | 0.003 | 152,808 | 6.191 | 0.013 | 83.8% |
| BMI | rs4740619 | 9 | 15,634,326 | *C9orf93* | T/C | 0.018 | 0.003 | **4.56E-09** | 321,887 |  | 0.016 | 0.004 | 5.22E-05 | 171,857 |  | 0.020 | 0.004 | 8.84E-07 | 152,742 | 0.539 | 0.463 | 0.0% |
| BMI | rs6477694 | 9 | 111,932,342 | *EPB41L4B* | C/T | 0.017 | 0.003 | **2.67E-08** | 322,048 |  | 0.021 | 0.004 | 2.77E-07 | 171,926 |  | 0.014 | 0.004 | 0.001 | 152,831 | 1.633 | 0.201 | 38.8% |
| BMI | rs11191560 | 10 | 104,869,038 | *NT5C2* | C/T | 0.031 | 0.005 | **8.45E-09** | 321,893 |  | 0.031 | 0.007 | 9.73E-06 | 171,798 |  | 0.031 | 0.007 | 1.01E-05 | 152,806 | 0.004 | 0.952 | 0.0% |
| BMI | rs17094222 | 10 | 102,395,440 | *HIF1AN* | C/T | 0.025 | 0.004 | **5.94E-11** | 321,770 |  | 0.020 | 0.005 | 3.22E-05 | 171,786 |  | 0.031 | 0.005 | **8.02E-10** | 152,695 | 2.165 | 0.141 | 53.8% |
| BMI | rs7899106 | 10 | 87,410,904 | *GRID1* | G/A | 0.040 | 0.007 | **2.96E-08** | 321,770 |  | 0.044 | 0.009 | 1.60E-06 | 171,711 |  | 0.036 | 0.009 | 1.17E-04 | 152,769 | 0.365 | 0.546 | 0.0% |
| BMI | rs7903146 | 10 | 114,758,349 | *TCF7L2* | C/T | 0.023 | 0.003 | **1.11E-11** | 322,130 |  | 0.018 | 0.004 | 3.97E-05 | 171,963 |  | 0.029 | 0.005 | **3.89E-11** | 152,877 | 3.281 | 0.070 | 69.5% |
| BMI | rs11030104 | 11 | 27,684,517 | *BDNF* | A/G | 0.041 | 0.004 | **5.56E-28** | 322,103 |  | 0.038 | 0.005 | **2.32E-15** | 171,949 |  | 0.045 | 0.005 | **4.70E-19** | 152,864 | 0.775 | 0.379 | 0.0% |
| BMI | rs12286929 | 11 | 115,022,404 | *CADM1* | G/A | 0.022 | 0.003 | **1.31E-12** | 321,903 |  | 0.021 | 0.004 | 8.95E-08 | 171,867 |  | 0.022 | 0.004 | **3.55E-08** | 152,747 | 0.072 | 0.788 | 0.0% |
| BMI | rs2176598 | 11 | 43,864,278 | *HSD17B12* | T/C | 0.020 | 0.004 | **2.97E-08** | 316,848 |  | 0.017 | 0.005 | 2.10E-04 | 168,915 |  | 0.024 | 0.005 | 3.78E-07 | 150,643 | 1.157 | 0.282 | 13.6% |
| BMI | rs3817334 | 11 | 47,650,993 | *MTCH2* | T/C | 0.026 | 0.003 | **5.15E-17** | 321,959 |  | 0.027 | 0.004 | **2.47E-11** | 171,889 |  | 0.026 | 0.004 | **1.89E-10** | 152,782 | 0.001 | 0.972 | 0.0% |
| BMI | rs4256980 | 11 | 8,673,939 | *TRIM66* | G/C | 0.021 | 0.003 | **2.90E-11** | 320,028 |  | 0.023 | 0.004 | **1.65E-08** | 171,168 |  | 0.019 | 0.004 | 9.37E-06 | 151,571 | 0.614 | 0.433 | 0.0% |
| BMI | rs11057405 | 12 | 122,781,897 | *CLIP1* | G/A | 0.031 | 0.006 | **2.02E-08** | 314,111 |  | 0.033 | 0.007 | 1.66E-06 | 167,373 |  | 0.027 | 0.007 | 1.90E-04 | 149,387 | 0.406 | 0.524 | 0.0% |
| BMI | rs7138803 | 12 | 50,247,468 | *BCDIN3D* | A/G | 0.032 | 0.003 | **8.15E-24** | 322,092 |  | 0.035 | 0.004 | **1.80E-17** | 171,945 |  | 0.027 | 0.004 | **5.90E-11** | 152,855 | 1.633 | 0.201 | 38.8% |
| BMI | rs12016871 | 13 | 26,915,782 | *MTIF3* | T/C | 0.030 | 0.005 | **2.29E-10** | 233,803 |  | 0.031 | 0.006 | 1.63E-07 | 132,007 |  | 0.027 | 0.006 | 1.07E-05 | 104,506 | 0.177 | 0.674 | 0.0% |
| BMI | rs12429545 | 13 | 54,102,206 | *OLFM4* | A/G | 0.033 | 0.005 | **1.09E-12** | 312,934 |  | 0.032 | 0.006 | 9.47E-08 | 166,760 |  | 0.035 | 0.006 | **1.51E-08** | 148,875 | 0.091 | 0.763 | 0.0% |
| BMI | rs1441264 | 13 | 79,580,919 | *MIR548A2* | A/G | 0.017 | 0.003 | **2.96E-08** | 310,286 |  | 0.017 | 0.004 | 4.37E-05 | 165,518 |  | 0.018 | 0.004 | 2.99E-05 | 147,478 | 0.014 | 0.905 | 0.0% |
| BMI | rs9540493 | 13 | 66,205,704 | *MIR548X2* | A/G | 0.021 | 0.004 | **4.97E-08** | 318,961 |  | 0.017 | 0.004 | 3.01E-05 | 170,383 |  | 0.017 | 0.004 | 6.92E-05 | 151,288 | 0.010 | 0.920 | 0.0% |
| BMI | rs10132280 | 14 | 25,928,179 | *STXBP6* | C/A | 0.023 | 0.003 | **1.14E-11** | 321,797 |  | 0.017 | 0.004 | 8.10E-05 | 171,796 |  | 0.029 | 0.004 | **4.97E-11** | 152,712 | 3.679 | 0.055 | 72.8% |
| BMI | rs11847697 | 14 | 30,515,112 | *PRKD1* | T/C | 0.049 | 0.008 | **3.99E-09** | 306,243 |  | 0.053 | 0.011 | 5.65E-07 | 162,904 |  | 0.045 | 0.011 | 3.97E-05 | 146,050 | 0.274 | 0.600 | 0.0% |
| BMI | rs12885454 | 14 | 29,736,838 | *PRKD1* | C/A | 0.021 | 0.003 | **1.94E-10** | 320,823 |  | 0.023 | 0.004 | **2.89E-08** | 171,309 |  | 0.019 | 0.004 | 9.82E-06 | 152,224 | 0.464 | 0.496 | 0.0% |
| BMI | rs7141420 | 14 | 79,899,454 | *NRXN3* | T/C | 0.024 | 0.003 | **1.23E-14** | 321,970 |  | 0.026 | 0.004 | **1.45E-11** | 171,881 |  | 0.022 | 0.004 | 6.24E-08 | 152,800 | 0.620 | 0.431 | 0.0% |
| BMI | rs16951275 | 15 | 68,077,168 | *MAP2K5* | T/C | 0.031 | 0.004 | **1.91E-17** | 322,098 |  | 0.030 | 0.005 | **2.96E-10** | 171,956 |  | 0.032 | 0.005 | **8.53E-11** | 152,853 | 0.069 | 0.793 | 0.0% |
| BMI | rs3736485 | 15 | 51,748,610 | *DMXL2* | A/G | 0.018 | 0.003 | **7.41E-09** | 321,398 |  | 0.019 | 0.004 | 2.68E-06 | 171,600 |  | 0.017 | 0.004 | 4.17E-05 | 152,509 | 0.101 | 0.750 | 0.0% |
| BMI | rs7164727 | 15 | 73,093,991 | *LOC100287559* | T/C | 0.019 | 0.003 | **3.92E-09** | 321,312 |  | 0.019 | 0.004 | 9.18E-06 | 171,483 |  | 0.017 | 0.004 | 8.42E-05 | 152,540 | 0.088 | 0.767 | 0.0% |
| BMI | rs12446632 | 16 | 19,935,389 | *GPRC5B* | G/A | 0.040 | 0.005 | **1.48E-18** | 316,758 |  | 0.043 | 0.006 | **1.45E-13** | 168,883 |  | 0.039 | 0.006 | **1.82E-10** | 150,586 | 0.317 | 0.573 | 0.0% |
| BMI | rs1558902 | 16 | 53,803,574 | *FTO* | A/T | 0.082 | 0.003 | **7.51E-153** | 320,073 |  | 0.079 | 0.004 | **4.03E-84** | 171,047 |  | 0.084 | 0.004 | **1.04E-93** | 151,737 | 0.774 | 0.379 | 0.0% |
| BMI | rs2080454 | 16 | 49,062,590 | *CBLN1* | C/A | 0.017 | 0.003 | **8.60E-09** | 322,099 |  | 0.017 | 0.004 | 5.24E-05 | 171,950 |  | 0.017 | 0.004 | 5.69E-05 | 152,860 | 0.001 | 0.973 | 0.0% |
| BMI | rs2650492 | 16 | 28,333,411 | *SBK1* | A/G | 0.021 | 0.004 | **1.92E-09** | 319,464 |  | 0.020 | 0.004 | 5.04E-06 | 171,405 |  | 0.021 | 0.005 | 4.72E-06 | 150,759 | 0.041 | 0.840 | 0.0% |
| BMI | rs3888190 | 16 | 28,889,486 | *ATP2A1* | A/C | 0.031 | 0.003 | **3.14E-23** | 321,930 |  | 0.028 | 0.004 | **3.51E-12** | 171,857 |  | 0.035 | 0.004 | **1.32E-17** | 152,784 | 1.714 | 0.190 | 41.7% |
| BMI | rs4787491 | 16 | 30,015,337 | *INO80E* | G/A | 0.022 | 0.004 | **2.70E-08** | 267,491 |  | 0.018 | 0.004 | 2.23E-05 | 146,058 |  | 0.014 | 0.005 | 0.002 | 124,144 | 0.381 | 0.537 | 0.0% |
| BMI | rs758747 | 16 | 3,627,358 | *NLRC3* | T/C | 0.023 | 0.004 | **7.47E-10** | 308,688 |  | 0.019 | 0.005 | 3.57E-05 | 164,745 |  | 0.026 | 0.005 | 8.04E-08 | 146,607 | 0.965 | 0.326 | 0.0% |
| BMI | rs9925964 | 16 | 31,129,895 | *KAT8* | A/G | 0.019 | 0.003 | **8.11E-10** | 318,385 |  | 0.020 | 0.004 | 1.83E-06 | 168,712 |  | 0.018 | 0.004 | 1.10E-05 | 152,384 | 0.042 | 0.838 | 0.0% |
| BMI | rs1000940 | 17 | 5,283,252 | *RABEP1* | G/A | 0.019 | 0.003 | **1.28E-08** | 321,836 |  | 0.020 | 0.004 | 2.20E-06 | 171,776 |  | 0.018 | 0.005 | 4.02E-05 | 152,768 | 0.093 | 0.760 | 0.0% |
| BMI | rs12940622 | 17 | 78,615,571 | *RPTOR* | G/A | 0.018 | 0.003 | **2.49E-09** | 322,032 |  | 0.016 | 0.004 | 4.76E-05 | 171,920 |  | 0.021 | 0.004 | 4.97E-07 | 152,823 | 0.617 | 0.432 | 0.0% |
| BMI | rs9914578 | 17 | 2,005,136 | *SMG6* | G/C | 0.020 | 0.004 | **2.07E-08** | 321,126 |  | 0.014 | 0.005 | 0.005 | 171,838 |  | 0.026 | 0.005 | 7.60E-08 | 151,999 | 3.482 | 0.062 | 71.3% |
| BMI | rs1808579 | 18 | 21,104,888 | *C18orf8* | C/T | 0.017 | 0.003 | **4.17E-08** | 322,032 |  | 0.022 | 0.004 | **1.23E-08** | 171,894 |  | 0.010 | 0.004 | 0.011 | 152,848 | 4.497 | 0.034 | 77.8% |
| BMI | rs6567160 | 18 | 57,829,135 | *MC4R* | C/T | 0.056 | 0.004 | **3.93E-53** | 321,958 |  | 0.056 | 0.005 | **5.08E-34** | 171,875 |  | 0.055 | 0.005 | **4.55E-30** | 152,793 | 0.065 | 0.798 | 0.0% |
| BMI | rs7239883 | 18 | 40,147,671 | *LOC284260* | G/A | 0.023 | 0.004 | **1.51E-08** | 321,909 |  | 0.023 | 0.004 | **1.51E-08** | 171,837 |  | 0.011 | 0.004 | 0.010 | 152,781 | 4.463 | 0.035 | 77.6% |
| BMI | rs7243357 | 18 | 56,883,319 | *GRP* | T/G | 0.022 | 0.004 | **3.86E-08** | 322,107 |  | 0.018 | 0.005 | 5.55E-04 | 171,948 |  | 0.025 | 0.005 | 1.40E-06 | 152,870 | 1.125 | 0.289 | 11.1% |
| BMI | rs17724992 | 19 | 18,454,825 | *PGPEP1* | A/G | 0.019 | 0.004 | **3.42E-08** | 319,588 |  | 0.016 | 0.005 | 4.82E-04 | 170,729 |  | 0.023 | 0.005 | 1.24E-06 | 151,569 | 1.124 | 0.289 | 11.1% |
| BMI | rs2075650 | 19 | 45,395,619 | *TOMM40* | A/G | 0.026 | 0.005 | **1.25E-08** | 308,408 |  | 0.031 | 0.006 | 1.06E-07 | 165,938 |  | 0.021 | 0.006 | 5.80E-04 | 145,181 | 1.553 | 0.213 | 35.6% |
| BMI | rs2287019 | 19 | 46,202,172 | *QPCTL* | C/T | 0.036 | 0.004 | **4.59E-18** | 300,921 |  | 0.032 | 0.005 | **8.60E-10** | 163,797 |  | 0.039 | 0.006 | **2.69E-12** | 139,830 | 0.659 | 0.417 | 0.0% |
| BMI | rs29941 | 19 | 34,309,532 | *KCTD15* | G/A | 0.018 | 0.003 | **2.41E-08** | 321,970 |  | 0.019 | 0.004 | 7.75E-06 | 171,887 |  | 0.018 | 0.004 | 3.27E-05 | 152,791 | 0.034 | 0.853 | 0.0% |
| BMI | rs3810291 | 19 | 47,569,003 | *ZC3H4* | A/G | 0.028 | 0.004 | **4.81E-15** | 296,261 |  | 0.030 | 0.005 | **2.60E-10** | 159,353 |  | 0.028 | 0.005 | **7.40E-09** | 139,619 | 0.072 | 0.789 | 0.0% |
| BMI | rs6091540 | 20 | 51,087,862 | *ZFP64* | C/T | 0.030 | 0.004 | **2.15E-11** | 321,975 |  | 0.030 | 0.004 | **2.15E-11** | 171,875 |  | 0.007 | 0.005 | 0.126 | 152,811 | 12.605 | **3.85E-04** | 92.1% |
| BMI | rs2836754 | 21 | 40,291,740 | *ETS2* | C/T | 0.017 | 0.003 | **1.61E-08** | 320,231 |  | 0.019 | 0.004 | 6.83E-06 | 171,028 |  | 0.014 | 0.004 | 5.96E-04 | 151,914 | 0.512 | 0.474 | 0.0% |
| WHR | rs2645294 | 1 | 119,376,110 | *TBX15-WARS2* | T/C | 0.031 | 0.004 | **1.69E-19** | 209,808 |  | 0.035 | 0.005 | **1.53E-14** | 116,596 |  | 0.027 | 0.005 | 1.46E-07 | 93,346 | 1.280 | 0.258 | 21.9% |
| WHR | rs905938 | 1 | 153,258,013 | *DCST2* | T/C | 0.025 | 0.004 | **7.34E-10** | 207,867 |  | 0.034 | 0.005 | **4.93E-10** | 115,536 |  | 0.015 | 0.006 | 0.011 | 92,461 | 5.918 | 0.015 | 83.1% |
| WHR | rs10919388 | 1 | 168,639,127 | *GORAB* | C/A | 0.024 | 0.004 | **3.18E-09** | 181,049 |  | 0.033 | 0.005 | **4.81E-10** | 102,446 |  | 0.013 | 0.006 | 0.030 | 78,738 | 6.557 | 0.010 | 84.8% |
| WHR | rs714515 | 1 | 170,619,613 | *DNM3-PIGC* | G/A | 0.027 | 0.003 | **4.38E-15** | 203,401 |  | 0.029 | 0.005 | **1.80E-10** | 113,939 |  | 0.025 | 0.005 | 8.54E-07 | 89,596 | 0.320 | 0.572 | 0.0% |
| WHR | rs2820443 | 1 | 217,820,132 | *LYPLAL1* | T/C | 0.035 | 0.004 | **5.26E-21** | 209,975 |  | 0.062 | 0.005 | **5.70E-35** | 116,672 |  | 0.002 | 0.005 | 0.691 | 93,437 | 72.000 | **2.15E-17** | 98.6% |
| WHR | rs1385167 | 2 | 66,054,152 | *MEIS1* | G/A | 0.029 | 0.005 | **1.85E-09** | 206,619 |  | 0.023 | 0.007 | 3.97E-04 | 114,668 |  | 0.036 | 0.007 | 2.32E-07 | 92,085 | 1.724 | 0.189 | 42.0% |
| WHR | rs10195252 | 2 | 165,221,337 | *GRB14-COBLL1* | T/C | 0.027 | 0.004 | **5.87E-15** | 209,395 |  | 0.052 | 0.005 | **4.68E-30** | 116,329 |  | -0.003 | 0.005 | 0.533 | 93,199 | 60.500 | **7.36E-15** | 98.3% |
| WHR | rs1569135 | 2 | 187,823,643 | *CALCRL* | A/G | 0.021 | 0.003 | **5.61E-10** | 209,906 |  | 0.023 | 0.005 | 6.86E-07 | 116,642 |  | 0.019 | 0.005 | 1.48E-04 | 93,398 | 0.320 | 0.572 | 0.0% |
| WHR | rs17819328 | 3 | 12,464,342 | *PPARG* | G/T | 0.021 | 0.004 | **2.43E-09** | 208,809 |  | 0.035 | 0.005 | **4.63E-14** | 116,072 |  | 0.005 | 0.005 | 0.326 | 92,871 | 18.000 | **2.21E-05** | 94.4% |
| WHR | rs2276824 | 3 | 52,612,526 | *PBRM1* | C/G | 0.024 | 0.004 | **3.17E-11** | 208,901 |  | 0.028 | 0.005 | **3.66E-09** | 116,128 |  | 0.020 | 0.005 | 1.35E-04 | 92,907 | 1.280 | 0.258 | 21.9% |
| WHR | rs2371767 | 3 | 64,693,298 | *ADAMTS9* | G/C | 0.036 | 0.004 | **1.59E-20** | 194,506 |  | 0.056 | 0.005 | **1.24E-26** | 108,624 |  | 0.012 | 0.006 | 0.035 | 86,016 | 31.738 | **1.76E-08** | 96.8% |
| WHR | rs10804591 | 3 | 130,816,923 | *PLXND1* | A/C | 0.025 | 0.004 | **6.57E-09** | 209,921 |  | 0.040 | 0.006 | **6.09E-13** | 116,667 |  | 0.004 | 0.006 | 0.528 | 93,387 | 18.000 | **2.21E-05** | 94.4% |
| WHR | rs17451107 | 3 | 158,280,303 | *LEKR1* | T/C | 0.026 | 0.004 | **1.14E-12** | 207,795 |  | 0.023 | 0.005 | 1.01E-06 | 115,735 |  | 0.030 | 0.005 | **1.42E-08** | 92,194 | 0.980 | 0.322 | 0.0% |
| WHR | rs3805389 | 4 | 56,177,507 | *NMU* | A/G | 0.012 | 0.004 | 0.001 | 209,218 |  | 0.027 | 0.005 | **4.64E-08** | 116,226 |  | -0.007 | 0.006 | 0.209 | 93,125 | 18.951 | **1.34E-05** | 94.7% |
| WHR | rs9991328 | 4 | 89,932,144 | *FAM13A* | T/C | 0.019 | 0.003 | **4.45E-08** | 209,925 |  | 0.028 | 0.005 | **3.43E-10** | 116,652 |  | 0.007 | 0.005 | 0.169 | 93,407 | 8.820 | 0.003 | 88.7% |
| WHR | rs303084 | 4 | 124,286,398 | *SPATA5-FGF2* | A/G | 0.023 | 0.004 | **3.88E-08** | 209,941 |  | 0.029 | 0.006 | 3.43E-07 | 116,662 |  | 0.016 | 0.006 | 0.010 | 93,412 | 2.347 | 0.126 | 57.4% |
| WHR | rs9687846 | 5 | 55,897,651 | *MAP3K1* | A/G | 0.024 | 0.005 | 7.11E-08 | 208,181 |  | 0.041 | 0.006 | **3.75E-12** | 115,897 |  | 0.000 | 0.007 | 0.969 | 92,417 | 19.776 | **8.70E-06** | 94.9% |
| WHR | rs1045241 | 5 | 118,757,185 | *TNFAIP8-HSD17B4* | C/T | 0.019 | 0.004 | 4.41E-07 | 209,710 |  | 0.035 | 0.005 | **6.63E-12** | 116,560 |  | -0.001 | 0.006 | 0.929 | 93,284 | 21.246 | **4.04E-06** | 95.3% |
| WHR | rs7705502 | 5 | 173,253,421 | *CPEB4* | A/G | 0.027 | 0.004 | **4.66E-14** | 209,827 |  | 0.027 | 0.005 | **1.93E-08** | 116,609 |  | 0.027 | 0.005 | 2.30E-07 | 93,352 | 0.000 | 1.000 | 0.0% |
| WHR | rs6556301 | 5 | 176,460,183 | *FGFR4* | T/G | 0.022 | 0.004 | **2.58E-08** | 178,874 |  | 0.018 | 0.005 | 7.05E-04 | 101,638 |  | 0.029 | 0.006 | 1.00E-06 | 77,370 | 1.984 | 0.159 | 49.6% |
| WHR | rs1294410 | 6 | 6,683,751 | *LY86* | C/T | 0.031 | 0.004 | **2.03E-18** | 209,830 |  | 0.037 | 0.005 | **1.59E-15** | 116,624 |  | 0.025 | 0.005 | 1.37E-06 | 93,340 | 2.880 | 0.090 | 65.3% |
| WHR | rs7759742 | 6 | 32,489,714 | *BTNL2* | A/T | 0.023 | 0.003 | **4.41E-11** | 208,263 |  | 0.024 | 0.005 | 1.73E-07 | 115,648 |  | 0.023 | 0.005 | 5.49E-06 | 92,749 | 0.020 | 0.888 | 0.0% |
| WHR | rs1776897 | 6 | 34,302,989 | *HMGA1* | G/T | 0.030 | 0.007 | 1.12E-05 | 177,879 |  | 0.052 | 0.009 | **6.84E-09** | 100,516 |  | 0.003 | 0.010 | 0.742 | 77,497 | 13.265 | **2.70E-04** | 92.5% |
| WHR | rs1358980 | 6 | 43,872,529 | *VEGFA* | T/C | 0.039 | 0.004 | **3.11E-27** | 206,862 |  | 0.060 | 0.005 | **3.74E-34** | 115,047 |  | 0.015 | 0.005 | 0.004 | 91,949 | 40.500 | **1.97E-10** | 97.5% |
| WHR | rs1936805 | 6 | 127,493,809 | *RSPO3* | T/C | 0.043 | 0.003 | **3.56E-35** | 209,859 |  | 0.052 | 0.005 | **3.67E-30** | 116,602 |  | 0.031 | 0.005 | **3.08E-10** | 93,392 | 8.820 | 0.003 | 88.7% |
| WHR | rs10245353 | 7 | 25,825,139 | *NFE2L3* | A/C | 0.035 | 0.004 | **8.41E-16** | 210,008 |  | 0.041 | 0.006 | **7.91E-13** | 116,704 |  | 0.027 | 0.006 | 1.43E-05 | 93,438 | 2.722 | 0.099 | 63.3% |
| WHR | rs7801581 | 7 | 27,190,296 | *HOXA11* | T/C | 0.027 | 0.004 | **3.68E-10** | 195,215 |  | 0.025 | 0.006 | 7.66E-06 | 108,866 |  | 0.029 | 0.006 | 2.39E-06 | 86,483 | 0.222 | 0.637 | 0.0% |
| WHR | rs7830933 | 8 | 23,659,269 | *NKX2-6* | A/G | 0.022 | 0.004 | 7.45E-08 | 209,766 |  | 0.037 | 0.005 | **1.23E-12** | 116,567 |  | 0.001 | 0.006 | 0.835 | 93,333 | 21.246 | **4.04E-06** | 95.3% |
| WHR | rs12679556 | 8 | 72,676,782 | *MSC* | G/T | 0.027 | 0.004 | **2.14E-11** | 203,826 |  | 0.033 | 0.005 | **2.13E-10** | 114,369 |  | 0.017 | 0.006 | 0.004 | 89,591 | 4.197 | 0.041 | 76.2% |
| WHR | rs10991437 | 9 | 106,775,741 | *ABCA1* | A/C | 0.031 | 0.005 | **1.02E-08** | 209,941 |  | 0.040 | 0.007 | **2.76E-08** | 116,644 |  | 0.022 | 0.008 | 0.006 | 93,430 | 2.867 | 0.090 | 65.1% |
| WHR | rs7917772 | 10 | 104,477,433 | *SFXN2* | A/G | 0.014 | 0.004 | 5.64E-05 | 209,642 |  | 0.027 | 0.005 | **5.50E-09** | 116,514 |  | -0.001 | 0.005 | 0.857 | 93,263 | 15.680 | **7.50E-05** | 93.6% |
| WHR | rs11231693 | 11 | 63,619,188 | *MACROD1-VEGFB* | A/G | 0.041 | 0.008 | **4.48E-08** | 198,072 |  | 0.068 | 0.010 | **2.68E-11** | 110,164 |  | 0.009 | 0.011 | 0.420 | 88,043 | 15.751 | **7.22E-05** | 93.7% |
| WHR | rs10842707 | 12 | 26,362,631 | *ITPR2-SSPN* | T/C | 0.032 | 0.004 | **4.40E-16** | 210,023 |  | 0.041 | 0.005 | **6.06E-15** | 116,704 |  | 0.022 | 0.006 | 1.44E-04 | 93,453 | 5.918 | 0.015 | 83.1% |
| WHR | rs1443512 | 12 | 52,628,951 | *HOXC13* | A/C | 0.028 | 0.004 | **6.94E-13** | 209,980 |  | 0.040 | 0.005 | **1.13E-14** | 116,688 |  | 0.013 | 0.006 | 0.028 | 93,425 | 11.951 | **0.001** | 91.6% |
| WHR | rs4765219 | 12 | 123,006,063 | *CCDC92* | C/A | 0.028 | 0.004 | **1.56E-15** | 209,807 |  | 0.037 | 0.005 | **1.00E-14** | 116,592 |  | 0.018 | 0.005 | 5.32E-04 | 93,350 | 7.220 | 0.007 | 86.1% |
| WHR | rs8042543 | 15 | 29,495,555 | *KLF13* | C/T | 0.026 | 0.004 | **1.16E-09** | 208,255 |  | 0.023 | 0.006 | 6.69E-05 | 115,760 |  | 0.030 | 0.006 | 1.01E-06 | 92,629 | 0.681 | 0.409 | 0.0% |
| WHR | rs8030605 | 15 | 54,291,890 | *RFX7* | A/G | 0.030 | 0.005 | **8.77E-09** | 208,374 |  | 0.031 | 0.007 | 1.00E-05 | 115,864 |  | 0.031 | 0.008 | 5.91E-05 | 92,644 | 0.000 | 1.000 | 0.0% |
| WHR | rs1440372 | 15 | 64,820,205 | *SMAD6* | C/T | 0.024 | 0.004 | **1.13E-10** | 207,447 |  | 0.022 | 0.005 | 1.09E-05 | 115,201 |  | 0.027 | 0.006 | 1.39E-06 | 92,380 | 0.410 | 0.522 | 0.0% |
| WHR | rs2925979 | 16 | 80,092,291 | *CMIP* | T/C | 0.018 | 0.004 | 1.22E-06 | 207,828 |  | 0.032 | 0.005 | **3.40E-11** | 115,431 |  | -0.002 | 0.005 | 0.786 | 92,531 | 23.120 | **1.52E-06** | 95.7% |
| WHR | rs4646404 | 17 | 17,360,924 | *PEMT* | G/A | 0.027 | 0.004 | **1.36E-11** | 198,196 |  | 0.034 | 0.005 | **5.28E-11** | 115,337 |  | 0.017 | 0.006 | 0.002 | 87,857 | 4.738 | 0.030 | 78.9% |
| WHR | rs8066985 | 17 | 65,964,940 | *KCNJ2* | A/G | 0.018 | 0.003 | 1.43E-07 | 209,977 |  | 0.026 | 0.005 | **4.02E-09** | 116,683 |  | 0.007 | 0.005 | 0.189 | 93,428 | 7.220 | 0.007 | 86.1% |
| WHR | rs12454712 | 18 | 58,996,864 | *BCL2* | T/C | 0.016 | 0.004 | 1.02E-04 | 169,793 |  | 0.035 | 0.006 | **1.13E-09** | 96,182 |  | -0.007 | 0.006 | 0.245 | 73,576 | 24.500 | **7.43E-07** | 95.9% |
| WHR | rs12608504 | 19 | 18,250,135 | *JUND* | A/G | 0.022 | 0.004 | **8.79E-10** | 209,990 |  | 0.017 | 0.005 | 2.65E-04 | 116,689 |  | 0.028 | 0.005 | 1.05E-07 | 93,435 | 2.420 | 0.120 | 58.7% |
| WHR | rs4081724 | 19 | 38,516,786 | *CEBPA* | G/A | 0.035 | 0.005 | **7.38E-12** | 207,418 |  | 0.033 | 0.007 | 9.19E-07 | 115,322 |  | 0.039 | 0.007 | 1.41E-07 | 92,230 | 0.367 | 0.544 | 0.0% |
| WHR | rs979012 | 20 | 6,571,374 | *BMP2* | T/C | 0.027 | 0.004 | **3.34E-14** | 209,941 |  | 0.026 | 0.005 | 1.04E-07 | 116,668 |  | 0.028 | 0.005 | 6.59E-08 | 93,407 | 0.080 | 0.777 | 0.0% |
| WHR | rs224333 | 20 | 33,487,376 | *GDF5* | G/A | 0.020 | 0.004 | **2.57E-08** | 208,025 |  | 0.009 | 0.005 | 0.074 | 115,803 |  | 0.036 | 0.005 | **9.00E-12** | 92,356 | 14.580 | **1.34E-04** | 93.1% |
| WHR | rs6090583 | 20 | 44,992,238 | *EYA2* | A/G | 0.022 | 0.003 | **6.17E-11** | 209,435 |  | 0.029 | 0.005 | **2.79E-10** | 116,382 |  | 0.015 | 0.005 | 0.002 | 93,187 | 3.920 | 0.048 | 74.5% |
| WHR | rs2294239 | 22 | 27,779,477 | *ZNRF3-KREMEN1* | A/G | 0.025 | 0.004 | **7.24E-13** | 209,454 |  | 0.028 | 0.005 | **6.94E-10** | 116,414 |  | 0.024 | 0.005 | 2.31E-06 | 93,173 | 0.320 | 0.572 | 0.0% |
| WC | rs9435732 | 1 | 17,180,745 | *MFAP2* | C/T | 0.031 | 0.004 | **4.12E-16** | 228,579 |  | 0.027 | 0.005 | **3.01E-08** | 125,711 |  | 0.037 | 0.005 | **9.35E-12** | 103,061 | 2.000 | 0.157 | 50.0% |
| WC | rs7536458 | 1 | 118,666,125 | *SPAG17* | T/G | 0.031 | 0.004 | **1.24E-15** | 228,790 |  | 0.026 | 0.005 | 2.21E-07 | 126,018 |  | 0.038 | 0.006 | **4.37E-12** | 102,965 | 2.361 | 0.124 | 57.6% |
| WC | rs10923712 | 1 | 119,306,957 | *TBX15-WARS2* | A/G | 0.035 | 0.004 | **1.07E-24** | 230,293 |  | 0.035 | 0.005 | **2.20E-15** | 127,380 |  | 0.035 | 0.005 | **1.10E-12** | 103,106 | 0.000 | 1.000 | 0.0% |
| WC | rs11205277 | 1 | 148,159,496 | *SF3B4* | G/A | 0.027 | 0.004 | **1.34E-13** | 215,898 |  | 0.024 | 0.005 | 2.36E-07 | 119,052 |  | 0.029 | 0.005 | **1.25E-08** | 97,039 | 0.500 | 0.480 | 0.0% |
| WC | rs2274432 | 1 | 182,287,568 | *TSEN15* | A/G | 0.025 | 0.004 | **1.75E-12** | 227,843 |  | 0.027 | 0.005 | **6.80E-09** | 125,694 |  | 0.023 | 0.005 | 4.08E-06 | 102,341 | 0.320 | 0.572 | 0.0% |
| WC | rs12048049 | 1 | 216,663,920 | *TGFB2* | G/C | 0.026 | 0.004 | **2.45E-12** | 230,423 |  | 0.023 | 0.005 | 1.75E-06 | 126,910 |  | 0.028 | 0.005 | 6.20E-08 | 103,705 | 0.500 | 0.480 | 0.0% |
| WC | rs3897379 | 1 | 217,826,356 | *LYPLAL1* | A/G | 0.016 | 0.004 | 2.12E-04 | 231,225 |  | 0.031 | 0.006 | **1.53E-08** | 127,406 |  | -0.002 | 0.006 | 0.766 | 104,012 | 15.125 | **1.01E-04** | 93.4% |
| WC | rs12127195 | 1 | 219,376,040 | *HLX* | A/G | 0.021 | 0.004 | **7.67E-09** | 231,166 |  | 0.015 | 0.005 | 0.002 | 127,373 |  | 0.028 | 0.005 | **3.36E-08** | 103,986 | 3.380 | 0.066 | 70.4% |
| WC | rs10925060 | 1 | 245,717,763 | *OR2W5-NLRP3* | T/C | 0.017 | 0.004 | 2.20E-05 | 140,515 |  | 0.002 | 0.005 | 0.681 | 85,186 |  | 0.045 | 0.006 | **9.14E-13** | 55,522 | 30.311 | **3.68E-08** | 96.7% |
| WC | rs12991495 | 2 | 25,340,274 | *DNMT3A* | T/C | 0.028 | 0.004 | **6.18E-14** | 229,964 |  | 0.026 | 0.005 | 1.52E-07 | 126,730 |  | 0.032 | 0.005 | **2.31E-09** | 103,427 | 0.720 | 0.396 | 0.0% |
| WC | rs6715793 | 2 | 33,232,767 | *LTBP1* | T/C | 0.019 | 0.003 | **1.44E-08** | 231,071 |  | 0.016 | 0.004 | 1.83E-04 | 127,302 |  | 0.024 | 0.005 | 7.10E-07 | 103,962 | 1.561 | 0.212 | 35.9% |
| WC | rs3791679 | 2 | 55,950,396 | *EFEMP1* | A/G | 0.035 | 0.004 | **2.06E-19** | 228,968 |  | 0.021 | 0.005 | 3.76E-05 | 126,097 |  | 0.053 | 0.006 | **3.12E-21** | 103,063 | 16.787 | **4.18E-05** | 94.0% |
| WC | rs2052670 | 2 | 66,071,985 | *KRT18P33* | G/A | 0.020 | 0.004 | **1.53E-08** | 231,210 |  | 0.020 | 0.005 | 1.23E-05 | 127,394 |  | 0.018 | 0.005 | 2.05E-04 | 104,009 | 0.080 | 0.777 | 0.0% |
| WC | rs2124969 | 2 | 160,697,732 | *ITGB6* | C/T | 0.020 | 0.003 | **7.06E-09** | 231,284 |  | 0.016 | 0.004 | 3.48E-04 | 127,437 |  | 0.025 | 0.005 | 2.33E-07 | 104,039 | 1.976 | 0.160 | 49.4% |
| WC | rs10195252 | 2 | 165,221,337 | *GRB14-COBLL1* | T/C | 0.011 | 0.003 | 9.44E-04 | 229,802 |  | 0.035 | 0.004 | **3.74E-15** | 126,613 |  | -0.016 | 0.005 | 6.71E-04 | 103,381 | 63.439 | **1.65E-15** | 98.4% |
| WC | rs6437061 | 2 | 232,893,296 | *DIS3L2* | A/C | 0.017 | 0.004 | 1.77E-06 | 229,808 |  | 0.008 | 0.004 | 0.074 | 126,658 |  | 0.027 | 0.005 | **2.50E-08** | 103,343 | 8.805 | 0.003 | 88.6% |
| WC | rs12489828 | 3 | 52,542,054 | *NT5DC2* | G/T | 0.020 | 0.003 | **1.03E-08** | 225,456 |  | 0.027 | 0.004 | **5.81E-10** | 123,098 |  | 0.012 | 0.005 | 0.014 | 102,550 | 5.488 | 0.019 | 81.8% |
| WC | rs9860730 | 3 | 64,676,186 | *ADAMTS9* | A/G | 0.022 | 0.004 | **2.07E-09** | 231,227 |  | 0.040 | 0.005 | **7.80E-17** | 127,402 |  | 0.001 | 0.005 | 0.861 | 104,018 | 30.420 | **3.48E-08** | 96.7% |
| WC | rs12330322 | 3 | 72,538,045 | *RYBP* | C/T | 0.022 | 0.004 | **3.15E-08** | 229,015 |  | 0.017 | 0.005 | 0.002 | 126,120 |  | 0.030 | 0.006 | 3.64E-07 | 103,088 | 2.770 | 0.096 | 63.9% |
| WC | rs6772896 | 3 | 135,686,037 | *ANAPC13-CEP63* | T/C | 0.024 | 0.004 | **1.79E-11** | 231,246 |  | 0.023 | 0.005 | 8.33E-07 | 127,422 |  | 0.025 | 0.005 | 9.80E-07 | 104,017 | 0.080 | 0.777 | 0.0% |
| WC | rs7621331 | 3 | 137,244,617 | *PPP2R3A* | A/G | 0.021 | 0.004 | **9.41E-09** | 231,264 |  | 0.014 | 0.005 | 0.004 | 127,428 |  | 0.028 | 0.005 | **4.70E-08** | 104,029 | 3.920 | 0.048 | 74.5% |
| WC | rs1344674 | 3 | 142,607,876 | *ZBTB38* | G/A | 0.024 | 0.003 | **4.33E-13** | 231,241 |  | 0.018 | 0.004 | 3.05E-05 | 127,417 |  | 0.032 | 0.005 | **9.50E-12** | 104,018 | 4.780 | 0.029 | 79.1% |
| WC | rs17451107 | 3 | 158,280,303 | *LEKR1* | T/C | 0.027 | 0.004 | **1.25E-13** | 227,636 |  | 0.032 | 0.005 | **3.51E-12** | 125,592 |  | 0.020 | 0.005 | 7.42E-05 | 102,237 | 2.880 | 0.090 | 65.3% |
| WC | rs12493901 | 3 | 173,404,749 | *FNDC3B* | G/A | 0.021 | 0.003 | **8.28E-10** | 230,668 |  | 0.016 | 0.004 | 1.76E-04 | 127,119 |  | 0.028 | 0.005 | **5.47E-09** | 103,742 | 3.512 | 0.061 | 71.5% |
| WC | rs7684221 | 4 | 17,566,452 | *LCORL* | G/A | 0.026 | 0.005 | **4.15E-08** | 229,360 |  | 0.018 | 0.006 | 0.003 | 126,312 |  | 0.034 | 0.007 | 3.23E-07 | 103,241 | 3.012 | 0.083 | 66.8% |
| WC | rs2197271 | 4 | 82,379,372 | *PRKG2* | G/C | 0.029 | 0.004 | **2.17E-13** | 228,929 |  | 0.020 | 0.005 | 1.12E-04 | 125,149 |  | 0.039 | 0.006 | **6.30E-13** | 103,973 | 5.918 | 0.015 | 83.1% |
| WC | rs1812175 | 4 | 145,794,294 | *HHIP* | G/A | 0.033 | 0.005 | **3.95E-13** | 230,433 |  | 0.026 | 0.006 | 1.46E-05 | 126,983 |  | 0.043 | 0.006 | **1.83E-11** | 103,643 | 4.014 | 0.045 | 75.1% |
| WC | rs7733331 | 5 | 32,864,603 | *C5orf23* | T/C | 0.021 | 0.003 | **5.74E-10** | 231,253 |  | 0.012 | 0.004 | 0.008 | 127,412 |  | 0.033 | 0.005 | **9.62E-12** | 104,035 | 10.756 | 0.001 | 90.7% |
| WC | rs13173241 | 5 | 55,897,116 | *MAP3K1* | A/G | 0.026 | 0.004 | **1.65E-09** | 229,628 |  | 0.048 | 0.006 | **2.55E-18** | 126,424 |  | -0.002 | 0.006 | 0.759 | 103,398 | 34.722 | **3.80E-09** | 97.1% |
| WC | rs10041657 | 5 | 108,180,327 | *FER* | A/G | 0.025 | 0.004 | **2.88E-10** | 230,824 |  | 0.026 | 0.005 | 4.05E-07 | 127,446 |  | 0.024 | 0.006 | 3.26E-05 | 103,570 | 0.066 | 0.798 | 0.0% |
| WC | rs272869 | 5 | 131,705,896 | *SLC22A4* | G/A | 0.021 | 0.003 | **6.68E-10** | 229,935 |  | 0.018 | 0.004 | 3.19E-05 | 126,599 |  | 0.025 | 0.005 | 2.02E-07 | 103,528 | 1.195 | 0.274 | 16.3% |
| WC | rs17472426 | 5 | 159,626,935 | *CCNJL* | T/G | 0.014 | 0.007 | 0.031 | 217,564 |  | -0.014 | 0.009 | 0.105 | 119,804 |  | 0.052 | 0.010 | **4.34E-08** | 97,954 | 24.066 | **9.31E-07** | 95.8% |
| WC | rs4868125 | 5 | 171,214,480 | *FBXW11* | G/C | 0.022 | 0.004 | **2.93E-09** | 225,860 |  | 0.022 | 0.005 | 4.15E-06 | 124,757 |  | 0.022 | 0.005 | 3.11E-05 | 101,296 | 0.000 | 1.000 | 0.0% |
| WC | rs10516107 | 5 | 173,280,762 | *CPEB4* | A/G | 0.023 | 0.004 | **8.29E-11** | 231,310 |  | 0.029 | 0.005 | **3.36E-10** | 127,451 |  | 0.016 | 0.005 | 0.001 | 104,051 | 3.380 | 0.066 | 70.4% |
| WC | rs6556301 | 5 | 176,460,183 | *FGFR4* | T/G | 0.028 | 0.004 | **1.82E-12** | 191,245 |  | 0.022 | 0.005 | 2.00E-05 | 108,078 |  | 0.036 | 0.006 | **6.23E-10** | 83,358 | 3.213 | 0.073 | 68.9% |
| WC | rs7773004 | 6 | 26,375,734 | *HIST1H3G-HIST1H2BH* | A/G | 0.026 | 0.003 | **1.93E-15** | 229,812 |  | 0.021 | 0.004 | 1.27E-06 | 126,533 |  | 0.033 | 0.005 | **1.83E-12** | 103,472 | 3.512 | 0.061 | 71.5% |
| WC | rs1776897 | 6 | 34,302,989 | *HMGA1* | G/T | 0.061 | 0.007 | **5.58E-20** | 197,374 |  | 0.072 | 0.009 | **1.50E-16** | 110,346 |  | 0.048 | 0.010 | 7.90E-07 | 87,220 | 3.182 | 0.074 | 68.6% |
| WC | rs13210323 | 6 | 35,113,062 | *ANKS1A* | A/C | 0.022 | 0.004 | **1.45E-08** | 231,199 |  | 0.019 | 0.005 | 1.17E-04 | 127,390 |  | 0.024 | 0.006 | 1.27E-05 | 104,002 | 0.410 | 0.522 | 0.0% |
| WC | rs998584 | 6 | 43,865,874 | *VEGFA* | A/C | 0.029 | 0.004 | **6.45E-15** | 210,814 |  | 0.056 | 0.005 | **4.15E-30** | 117,469 |  | -0.003 | 0.005 | 0.557 | 93,538 | 69.620 | **7.19E-17** | 98.6% |
| WC | rs12207675 | 6 | 76,294,461 | *FILIP1* | C/T | 0.031 | 0.005 | **3.14E-09** | 231,322 |  | 0.030 | 0.007 | 6.64E-06 | 127,458 |  | 0.031 | 0.007 | 2.71E-05 | 104,058 | 0.010 | 0.920 | 0.0% |
| WC | rs395962 | 6 | 105,504,111 | *LIN28B* | T/G | 0.029 | 0.004 | **1.33E-15** | 231,306 |  | 0.022 | 0.005 | 1.97E-06 | 127,447 |  | 0.036 | 0.005 | **2.10E-12** | 104,052 | 3.920 | 0.048 | 74.5% |
| WC | rs2745353 | 6 | 127,494,628 | *RSPO3* | T/C | 0.029 | 0.003 | **7.88E-19** | 231,143 |  | 0.035 | 0.004 | **1.28E-15** | 127,339 |  | 0.022 | 0.005 | 3.14E-06 | 103,997 | 4.122 | 0.042 | 75.7% |
| WC | rs6570507 | 6 | 142,721,265 | *GPR126* | G/A | 0.025 | 0.004 | **5.73E-11** | 228,993 |  | 0.023 | 0.005 | 1.64E-06 | 126,106 |  | 0.027 | 0.005 | 4.96E-07 | 103,080 | 0.320 | 0.572 | 0.0% |
| WC | rs798502 | 7 | 2,756,406 | *GNA12* | A/C | 0.024 | 0.004 | **2.95E-11** | 228,949 |  | 0.018 | 0.005 | 2.04E-04 | 125,153 |  | 0.033 | 0.005 | **1.12E-10** | 103,988 | 4.500 | 0.034 | 77.8% |
| WC | rs2214442 | 7 | 20,359,312 | *ITGB8* | G/A | 0.027 | 0.005 | **3.89E-09** | 152,053 |  | 0.025 | 0.006 | 6.80E-06 | 91,400 |  | 0.028 | 0.007 | 3.37E-05 | 60,846 | 0.106 | 0.745 | 0.0% |
| WC | rs4141278 | 7 | 25,824,050 | *NFE2L3* | C/T | 0.034 | 0.004 | **3.39E-15** | 231,233 |  | 0.036 | 0.006 | **4.78E-11** | 127,415 |  | 0.031 | 0.006 | 2.06E-07 | 104,011 | 0.347 | 0.556 | 0.0% |
| WC | rs7801581 | 7 | 27,190,296 | *HOXA11* | T/C | 0.027 | 0.004 | **8.01E-11** | 216,463 |  | 0.026 | 0.005 | 2.21E-06 | 119,583 |  | 0.030 | 0.006 | 2.07E-07 | 97,073 | 0.262 | 0.609 | 0.0% |
| WC | rs849140 | 7 | 28,150,227 | *JAZF1* | T/C | 0.029 | 0.003 | **4.74E-17** | 228,910 |  | 0.032 | 0.004 | **5.41E-13** | 126,079 |  | 0.025 | 0.005 | 3.72E-07 | 103,023 | 1.195 | 0.274 | 16.3% |
| WC | rs822531 | 7 | 148,260,692 | *EZH2* | T/C | 0.024 | 0.004 | **3.71E-08** | 226,664 |  | 0.020 | 0.006 | 8.98E-04 | 124,836 |  | 0.030 | 0.006 | 1.57E-06 | 102,021 | 1.389 | 0.239 | 28.0% |
| WC | rs7830933 | 8 | 23,659,269 | *NKX2-6* | A/G | 0.017 | 0.004 | 9.01E-06 | 231,029 |  | 0.033 | 0.005 | **1.93E-10** | 127,293 |  | -0.002 | 0.006 | 0.755 | 103,929 | 20.082 | **7.42E-06** | 95.0% |
| WC | rs12679556 | 8 | 72,676,782 | *MSC* | G/T | 0.026 | 0.004 | **1.26E-11** | 225,056 |  | 0.035 | 0.005 | **1.05E-11** | 125,085 |  | 0.016 | 0.006 | 0.005 | 100,164 | 5.918 | 0.015 | 83.1% |
| WC | rs11144688 | 9 | 77,732,106 | *PCSK5* | G/A | 0.034 | 0.006 | **1.87E-08** | 214,513 |  | 0.033 | 0.008 | 4.44E-05 | 118,374 |  | 0.036 | 0.008 | 1.18E-05 | 96,332 | 0.070 | 0.791 | 0.0% |
| WC | rs473902 | 9 | 97,296,056 | *PTCH1* | T/G | 0.049 | 0.007 | **4.35E-12** | 204,544 |  | 0.050 | 0.009 | 5.35E-08 | 112,714 |  | 0.049 | 0.010 | 8.87E-07 | 92,023 | 0.006 | 0.941 | 0.0% |
| WC | rs3862030 | 10 | 104,317,574 | *SUFU* | A/G | 0.021 | 0.003 | **5.83E-10** | 231,146 |  | 0.028 | 0.004 | **1.77E-10** | 127,315 |  | 0.013 | 0.005 | 0.008 | 104,024 | 5.488 | 0.019 | 81.8% |
| WC | rs1784203 | 11 | 93,089,782 | *KIAA1731* | A/G | 0.031 | 0.005 | **1.31E-08** | 63,892 |  | 0.000 | 0.049 | 0.993 | 35,539 |  | 0.075 | 0.008 | **1.03E-19** | 28,353 | 2.282 | 0.131 | 56.2% |
| WC | rs2638953 | 12 | 28,425,682 | *CCDC91* | C/G | 0.024 | 0.004 | **6.53E-11** | 228,074 |  | 0.021 | 0.005 | 9.51E-06 | 125,951 |  | 0.027 | 0.005 | 1.30E-07 | 102,316 | 0.720 | 0.396 | 0.0% |
| WC | rs2071449 | 12 | 52,714,278 | *HOXC5* | A/C | 0.032 | 0.004 | **2.47E-18** | 226,567 |  | 0.029 | 0.005 | **4.53E-10** | 123,896 |  | 0.034 | 0.005 | **1.65E-11** | 102,864 | 0.500 | 0.480 | 0.0% |
| WC | rs7970350 | 12 | 64,646,431 | *HMGA2* | C/T | 0.019 | 0.003 | **3.76E-08** | 229,815 |  | 0.014 | 0.004 | 0.001 | 126,536 |  | 0.024 | 0.005 | 5.10E-07 | 103,472 | 2.439 | 0.118 | 59.0% |
| WC | rs12317176 | 12 | 122,970,671 | *DNAH10* | T/C | 0.021 | 0.004 | **5.88E-09** | 230,924 |  | 0.034 | 0.005 | **1.29E-13** | 127,243 |  | 0.003 | 0.005 | 0.547 | 103,874 | 19.220 | **1.16E-05** | 94.8% |
| WC | rs2160077 | 14 | 91,498,163 | *TRIP11* | G/A | 0.018 | 0.003 | **4.46E-08** | 231,255 |  | 0.012 | 0.004 | 0.006 | 127,415 |  | 0.026 | 0.005 | **2.62E-08** | 104,033 | 4.780 | 0.029 | 79.1% |
| WC | rs7166081 | 15 | 65,279,355 | *SMAD3* | A/G | 0.024 | 0.004 | **2.12E-09** | 230,255 |  | 0.017 | 0.005 | 9.35E-04 | 126,884 |  | 0.032 | 0.006 | **1.14E-08** | 103,561 | 3.689 | 0.055 | 72.9% |
| WC | rs4886782 | 15 | 72,015,863 | *LOXL1* | G/A | 0.025 | 0.004 | **5.98E-12** | 228,446 |  | 0.018 | 0.005 | 1.42E-04 | 125,776 |  | 0.032 | 0.005 | **3.22E-10** | 102,863 | 3.920 | 0.048 | 74.5% |
| WC | rs7162542 | 15 | 82,305,294 | *ADAMTSL3* | G/C | 0.038 | 0.003 | **9.75E-29** | 229,892 |  | 0.037 | 0.004 | **1.17E-17** | 126,671 |  | 0.039 | 0.005 | **2.86E-16** | 103,414 | 0.098 | 0.755 | 0.0% |
| WC | rs1879529 | 15 | 87,215,299 | *ACAN* | G/T | 0.024 | 0.004 | **2.86E-10** | 224,276 |  | 0.022 | 0.005 | 7.55E-06 | 124,928 |  | 0.024 | 0.006 | 1.72E-05 | 99,541 | 0.066 | 0.798 | 0.0% |
| WC | rs4246302 | 15 | 98,505,490 | *ADAMTS17* | G/A | 0.022 | 0.004 | **5.73E-09** | 227,205 |  | 0.025 | 0.005 | 2.88E-07 | 125,027 |  | 0.018 | 0.005 | 6.74E-04 | 102,371 | 0.980 | 0.322 | 0.0% |
| WC | rs2047937 | 16 | 48,422,292 | *ZNF423* | C/T | 0.019 | 0.003 | **4.67E-08** | 231,009 |  | 0.022 | 0.004 | 5.49E-07 | 127,288 |  | 0.014 | 0.005 | 0.004 | 103,914 | 1.561 | 0.212 | 35.9% |
| WC | rs16957304 | 16 | 65,892,470 | *KCTD19* | A/G | 0.059 | 0.011 | **2.47E-08** | 151,917 |  | 0.060 | 0.013 | 5.37E-06 | 91,174 |  | 0.058 | 0.016 | 3.18E-04 | 60,936 | 0.009 | 0.923 | 0.0% |
| WC | rs3760318 | 17 | 26,271,841 | *CENTA2* | G/A | 0.021 | 0.004 | **9.05E-10** | 228,998 |  | 0.018 | 0.005 | 4.78E-05 | 126,117 |  | 0.026 | 0.005 | 7.51E-08 | 103,074 | 1.280 | 0.258 | 21.9% |
| WC | rs882367 | 17 | 56,849,356 | *C17orf82* | C/T | 0.027 | 0.004 | **1.09E-13** | 229,876 |  | 0.026 | 0.005 | **3.54E-08** | 126,581 |  | 0.028 | 0.005 | **3.52E-08** | 103,489 | 0.080 | 0.777 | 0.0% |
| WC | rs4239436 | 18 | 18,985,928 | *CABLES1* | G/A | 0.041 | 0.004 | **1.01E-22** | 229,607 |  | 0.036 | 0.005 | **1.14E-11** | 126,423 |  | 0.045 | 0.006 | **1.46E-14** | 103,377 | 1.328 | 0.249 | 24.7% |
| WC | rs4542783 | 19 | 8,548,160 | *ADAMTS10* | T/C | 0.023 | 0.004 | **1.69E-08** | 170,860 |  | 0.019 | 0.005 | 2.05E-04 | 96,281 |  | 0.027 | 0.006 | 2.93E-06 | 74,773 | 1.049 | 0.306 | 4.7% |
| WC | rs12608504 | 19 | 18,250,135 | *JUND* | A/G | 0.020 | 0.004 | **1.48E-08** | 228,998 |  | 0.018 | 0.005 | 7.39E-05 | 126,113 |  | 0.022 | 0.005 | 6.65E-06 | 103,077 | 0.320 | 0.572 | 0.0% |
| WC | rs3786897 | 19 | 38,584,848 | *PEPD* | G/A | 0.020 | 0.004 | **8.77E-09** | 228,567 |  | 0.023 | 0.004 | 1.69E-07 | 125,873 |  | 0.015 | 0.005 | 0.002 | 102,887 | 1.561 | 0.212 | 35.9% |
| WC | rs1884897 | 20 | 6,560,832 | *BMP2* | A/G | 0.032 | 0.004 | **6.56E-20** | 229,025 |  | 0.035 | 0.005 | **5.92E-15** | 126,122 |  | 0.028 | 0.005 | **1.51E-08** | 103,096 | 0.980 | 0.322 | 0.0% |
| WC | rs9977276 | 21 | 46,260,755 | *COL6A1* | G/T | 0.022 | 0.004 | **4.37E-08** | 229,565 |  | 0.028 | 0.005 | 1.31E-07 | 126,398 |  | 0.015 | 0.006 | 0.009 | 103,360 | 2.770 | 0.096 | 63.9% |
| WC | rs2294239 | 22 | 27,779,477 | *ZNRF3* | A/G | 0.019 | 0.004 | **3.49E-08** | 228,463 |  | 0.026 | 0.004 | **4.19E-09** | 125,841 |  | 0.012 | 0.005 | 0.011 | 102,814 | 4.780 | 0.029 | 79.1% |
| HIP | rs6657613 | 1 | 17,200,787 | *ATP13A2* | T/A | 0.031 | 0.004 | **3.89E-18** | 210,917 |  | 0.027 | 0.005 | **3.75E-09** | 117,223 |  | 0.036 | 0.005 | **7.21E-13** | 93,880 | 1.620 | 0.203 | 38.3% |
| HIP | rs7521902 | 1 | 22,363,311 | *WNT4* | A/C | 0.024 | 0.004 | **3.38E-08** | 210,318 |  | 0.020 | 0.006 | 3.95E-04 | 116,952 |  | 0.029 | 0.006 | 2.80E-06 | 93,552 | 1.125 | 0.289 | 11.1% |
| HIP | rs12086130 | 1 | 50,946,191 | *FAF1* | T/C | 0.038 | 0.006 | **2.84E-09** | 206,610 |  | 0.024 | 0.008 | 0.002 | 115,998 |  | 0.055 | 0.009 | **2.23E-09** | 90,798 | 6.628 | 0.010 | 84.9% |
| HIP | rs3748656 | 1 | 113,038,204 | *MOV10* | C/T | 0.024 | 0.004 | **6.07E-09** | 210,890 |  | 0.027 | 0.005 | 9.24E-07 | 117,200 |  | 0.021 | 0.006 | 5.23E-04 | 93,876 | 0.590 | 0.442 | 0.0% |
| HIP | rs11205303 | 1 | 148,173,037 | *MTMR11* | C/T | 0.041 | 0.004 | **5.78E-25** | 196,314 |  | 0.042 | 0.005 | **1.59E-16** | 109,455 |  | 0.042 | 0.006 | **2.07E-13** | 87,045 | 0.000 | 1.000 | 0.0% |
| HIP | rs12744534 | 1 | 168,924,979 | *PRRX1* | G/A | 0.035 | 0.005 | **2.02E-10** | 210,115 |  | 0.033 | 0.007 | 1.67E-06 | 116,775 |  | 0.037 | 0.008 | 1.61E-06 | 93,525 | 0.142 | 0.707 | 0.0% |
| HIP | rs17346473 | 1 | 170,349,716 | *DNM3* | G/A | 0.030 | 0.004 | **2.86E-14** | 210,431 |  | 0.032 | 0.005 | **1.77E-10** | 116,885 |  | 0.026 | 0.006 | 4.33E-06 | 93,732 | 0.590 | 0.442 | 0.0% |
| HIP | rs1046934 | 1 | 182,290,152 | *TSEN15* | C/A | 0.023 | 0.004 | **5.51E-10** | 210,450 |  | 0.024 | 0.005 | 2.69E-07 | 116,969 |  | 0.022 | 0.005 | 2.66E-05 | 93,667 | 0.080 | 0.777 | 0.0% |
| HIP | rs2993535 | 1 | 210,221,815 | *INTS7* | G/A | 0.051 | 0.010 | 4.87E-07 | 132,078 |  | 0.031 | 0.012 | 0.012 | 80,570 |  | 0.083 | 0.015 | **4.16E-08** | 51,695 | 7.328 | 0.007 | 86.4% |
| HIP | rs991967 | 1 | 216,682,074 | *TGFB2* | C/A | 0.022 | 0.004 | **6.50E-09** | 210,314 |  | 0.025 | 0.005 | 4.13E-07 | 116,687 |  | 0.020 | 0.006 | 4.30E-04 | 93,813 | 0.410 | 0.522 | 0.0% |
| HIP | rs2820443 | 1 | 217,820,132 | *ZC3H11B* | C/T | 0.048 | 0.004 | **1.49E-35** | 211,030 |  | 0.062 | 0.005 | **4.36E-35** | 117,270 |  | 0.031 | 0.006 | **2.89E-08** | 93,946 | 15.754 | **7.21E-05** | 93.7% |
| HIP | rs6672530 | 1 | 225,837,839 | *ZNF678* | A/C | 0.028 | 0.005 | **8.43E-10** | 208,172 |  | 0.031 | 0.006 | 1.18E-07 | 115,486 |  | 0.026 | 0.007 | 9.61E-05 | 92,872 | 0.294 | 0.588 | 0.0% |
| HIP | rs1545552 | 2 | 33,213,842 | *LTBP1* | G/A | 0.029 | 0.004 | **6.06E-13** | 208,132 |  | 0.030 | 0.005 | **8.07E-09** | 115,791 |  | 0.030 | 0.006 | 3.50E-07 | 92,525 | 0.000 | 1.000 | 0.0% |
| HIP | rs3791679 | 2 | 55,950,396 | *EFEMP1* | A/G | 0.039 | 0.004 | **1.22E-20** | 210,979 |  | 0.033 | 0.005 | **6.47E-10** | 117,265 |  | 0.047 | 0.006 | **4.74E-15** | 93,900 | 3.213 | 0.073 | 68.9% |
| HIP | rs10195252 | 2 | 165,221,337 | *GRB14-COBLL1* | C/T | 0.023 | 0.004 | **1.06E-10** | 210,403 |  | 0.031 | 0.005 | **1.46E-11** | 116,927 |  | 0.012 | 0.005 | 0.017 | 93,661 | 7.220 | 0.007 | 86.1% |
| HIP | rs4973517 | 2 | 232,870,062 | *DIS3L2* | T/C | 0.029 | 0.005 | **1.93E-10** | 175,930 |  | 0.025 | 0.006 | 1.44E-05 | 99,828 |  | 0.035 | 0.007 | 1.92E-07 | 76,288 | 1.176 | 0.278 | 15.0% |
| HIP | rs6739772 | 2 | 241,489,384 | *C2orf54* | G/A | 0.022 | 0.004 | **1.24E-08** | 210,702 |  | 0.030 | 0.005 | **1.54E-09** | 117,108 |  | 0.014 | 0.006 | 0.015 | 93,778 | 4.197 | 0.041 | 76.2% |
| HIP | rs17819328 | 3 | 12,464,342 | *PPARG* | T/G | 0.023 | 0.004 | **8.56E-10** | 209,843 |  | 0.030 | 0.005 | **1.66E-10** | 116,670 |  | 0.015 | 0.005 | 0.003 | 93,359 | 4.500 | 0.034 | 77.8% |
| HIP | rs2597513 | 3 | 13,530,836 | *HDAC11* | C/T | 0.033 | 0.006 | **2.02E-08** | 210,951 |  | 0.036 | 0.008 | 1.50E-06 | 117,240 |  | 0.030 | 0.009 | 4.59E-04 | 93,897 | 0.248 | 0.618 | 0.0% |
| HIP | rs11242 | 3 | 53,100,962 | *RFT1* | T/C | 0.027 | 0.004 | **6.31E-14** | 204,637 |  | 0.021 | 0.005 | 3.61E-06 | 114,659 |  | 0.035 | 0.005 | **1.08E-11** | 90,164 | 3.920 | 0.048 | 74.5% |
| HIP | rs1388251 | 3 | 56,534,284 | *CCDC66* | A/G | 0.023 | 0.004 | **2.05E-08** | 211,029 |  | 0.017 | 0.005 | 0.001 | 117,297 |  | 0.029 | 0.006 | 4.39E-07 | 93,918 | 2.361 | 0.124 | 57.6% |
| HIP | rs10804591 | 3 | 130,816,923 | *PLXND1* | C/A | 0.038 | 0.004 | **7.42E-18** | 210,953 |  | 0.044 | 0.006 | **5.54E-15** | 117,265 |  | 0.032 | 0.006 | 6.41E-07 | 93,873 | 2.000 | 0.157 | 50.0% |
| HIP | rs724016 | 3 | 142,588,260 | *ZBTB38* | G/A | 0.048 | 0.004 | **7.53E-43** | 211,032 |  | 0.044 | 0.005 | **1.98E-22** | 117,305 |  | 0.055 | 0.005 | **1.11E-26** | 93,913 | 2.420 | 0.120 | 58.7% |
| HIP | rs4243400 | 3 | 173,453,553 | *FNDC3B* | G/A | 0.025 | 0.004 | **2.70E-12** | 210,478 |  | 0.028 | 0.005 | **1.43E-09** | 116,993 |  | 0.022 | 0.005 | 1.55E-05 | 93,671 | 0.720 | 0.396 | 0.0% |
| HIP | rs2098771 | 3 | 187,045,968 | *IGF2BP2* | G/A | 0.022 | 0.004 | **3.58E-08** | 196,732 |  | 0.021 | 0.005 | 9.22E-05 | 110,388 |  | 0.023 | 0.006 | 4.10E-05 | 86,530 | 0.066 | 0.798 | 0.0% |
| HIP | rs2247341 | 4 | 1,671,115 | *SLBP* | A/G | 0.023 | 0.004 | **6.49E-10** | 211,076 |  | 0.024 | 0.005 | 7.57E-07 | 117,317 |  | 0.022 | 0.005 | 2.75E-05 | 93,945 | 0.080 | 0.777 | 0.0% |
| HIP | rs6845078 | 4 | 17,561,306 | *LCORL* | C/T | 0.035 | 0.005 | **8.97E-12** | 207,534 |  | 0.027 | 0.007 | 2.80E-05 | 115,535 |  | 0.047 | 0.007 | **1.25E-10** | 92,185 | 4.082 | 0.043 | 75.5% |
| HIP | rs9993613 | 4 | 73,694,878 | *ADAMTS3* | T/G | 0.028 | 0.005 | **6.83E-10** | 143,494 |  | 0.029 | 0.005 | 1.37E-07 | 86,780 |  | 0.027 | 0.007 | 4.60E-05 | 56,901 | 0.054 | 0.816 | 0.0% |
| HIP | rs1662837 | 4 | 82,387,913 | *PRKG2* | C/T | 0.028 | 0.004 | **1.36E-13** | 210,825 |  | 0.031 | 0.005 | **2.36E-10** | 117,172 |  | 0.026 | 0.005 | 2.58E-06 | 93,840 | 0.500 | 0.480 | 0.0% |
| HIP | rs1812175 | 4 | 145,794,294 | *HHIP* | G/A | 0.059 | 0.005 | **1.57E-34** | 211,054 |  | 0.051 | 0.006 | **1.00E-16** | 117,296 |  | 0.067 | 0.007 | **3.94E-22** | 93,944 | 3.012 | 0.083 | 66.8% |
| HIP | rs11730399 | 4 | 146,394,371 | *OTUD4* | A/C | 0.060 | 0.008 | **5.00E-13** | 173,372 |  | 0.066 | 0.011 | **8.31E-10** | 98,345 |  | 0.054 | 0.012 | 4.41E-06 | 75,213 | 0.543 | 0.461 | 0.0% |
| HIP | rs1173771 | 5 | 32,850,785 | *NPR3* | A/G | 0.026 | 0.004 | **6.13E-13** | 210,986 |  | 0.024 | 0.005 | 1.43E-07 | 117,276 |  | 0.028 | 0.005 | 6.19E-08 | 93,896 | 0.320 | 0.572 | 0.0% |
| HIP | rs6556079 | 5 | 172,929,684 | *FAM44B* | G/A | 0.028 | 0.005 | **4.45E-10** | 143,741 |  | 0.024 | 0.005 | 1.05E-05 | 86,924 |  | 0.033 | 0.007 | 1.05E-06 | 57,005 | 1.095 | 0.295 | 8.6% |
| HIP | rs1294410 | 6 | 6,683,751 | *LY86* | T/C | 0.029 | 0.004 | **1.66E-15** | 210,861 |  | 0.030 | 0.005 | **2.23E-10** | 117,222 |  | 0.029 | 0.005 | **3.21E-08** | 93,825 | 0.020 | 0.888 | 0.0% |
| HIP | rs13216391 | 6 | 7,734,942 | *BMP6* | G/A | 0.028 | 0.005 | **2.65E-09** | 199,240 |  | 0.026 | 0.006 | 2.16E-05 | 110,870 |  | 0.031 | 0.007 | 3.77E-06 | 88,556 | 0.294 | 0.588 | 0.0% |
| HIP | rs806794 | 6 | 26,308,656 | *HIST1H2BF* | A/G | 0.046 | 0.004 | **2.01E-31** | 204,203 |  | 0.045 | 0.005 | **3.81E-19** | 114,444 |  | 0.048 | 0.006 | **2.55E-17** | 89,945 | 0.148 | 0.701 | 0.0% |
| HIP | rs12210905 | 6 | 27,259,288 | *HIST1H2AH* | A/G | 0.033 | 0.006 | **9.54E-09** | 210,929 |  | 0.033 | 0.008 | 8.08E-06 | 117,211 |  | 0.034 | 0.008 | 2.67E-05 | 93,904 | 0.008 | 0.930 | 0.0% |
| HIP | rs7741091 | 6 | 31,460,610 | *MICA* | A/G | 0.020 | 0.004 | 8.25E-08 | 210,219 |  | 0.012 | 0.005 | 0.015 | 116,466 |  | 0.031 | 0.005 | **1.69E-08** | 93,939 | 7.220 | 0.007 | 86.1% |
| HIP | rs1759645 | 6 | 34,302,844 | *HMGA1* | C/T | 0.029 | 0.005 | **9.20E-09** | 209,671 |  | 0.025 | 0.007 | 1.26E-04 | 116,169 |  | 0.035 | 0.007 | 2.18E-06 | 93,688 | 1.020 | 0.312 | 2.0% |
| HIP | rs16894959 | 6 | 34,933,640 | *UHRF1BP1* | C/T | 0.036 | 0.005 | **3.20E-13** | 210,242 |  | 0.030 | 0.006 | 3.11E-06 | 116,473 |  | 0.043 | 0.007 | **9.02E-10** | 93,955 | 1.988 | 0.159 | 49.7% |
| HIP | rs7739232 | 6 | 53,648,294 | *KLHL31* | A/G | 0.037 | 0.009 | 5.44E-05 | 131,877 |  | 0.063 | 0.011 | **1.03E-08** | 80,475 |  | -0.004 | 0.014 | 0.751 | 51,589 | 14.161 | **1.68E-04** | 92.9% |
| HIP | rs12207675 | 6 | 76,294,461 | *FILIP1* | C/T | 0.041 | 0.006 | **1.13E-13** | 211,077 |  | 0.037 | 0.007 | 1.22E-07 | 117,329 |  | 0.044 | 0.008 | **2.50E-08** | 93,934 | 0.434 | 0.510 | 0.0% |
| HIP | rs7759938 | 6 | 105,485,647 | *LIN28B* | C/T | 0.028 | 0.004 | **1.90E-13** | 211,029 |  | 0.023 | 0.005 | 1.98E-06 | 117,291 |  | 0.033 | 0.005 | **1.16E-09** | 93,924 | 2.000 | 0.157 | 50.0% |
| HIP | rs1538170 | 6 | 126,794,567 | *CENPW* | T/C | 0.026 | 0.004 | **2.48E-12** | 201,926 |  | 0.021 | 0.005 | 1.73E-05 | 112,080 |  | 0.034 | 0.005 | **2.19E-10** | 90,032 | 3.380 | 0.066 | 70.4% |
| HIP | rs9491696 | 6 | 127,494,332 | *RSPO3* | C/G | 0.023 | 0.004 | **1.45E-10** | 210,813 |  | 0.029 | 0.005 | **1.44E-10** | 117,182 |  | 0.015 | 0.005 | 0.003 | 93,817 | 3.920 | 0.048 | 74.5% |
| HIP | rs9388766 | 6 | 130,396,548 | *L3MBTL3* | T/C | 0.028 | 0.004 | **7.47E-13** | 211,072 |  | 0.024 | 0.005 | 1.88E-06 | 117,322 |  | 0.033 | 0.006 | **4.56E-09** | 93,936 | 1.328 | 0.249 | 24.7% |
| HIP | rs6570509 | 6 | 142,757,979 | *GPR126* | G/T | 0.045 | 0.004 | **1.23E-29** | 197,803 |  | 0.042 | 0.005 | **3.15E-16** | 110,322 |  | 0.048 | 0.006 | **2.47E-16** | 87,667 | 0.590 | 0.442 | 0.0% |
| HIP | rs798497 | 7 | 2,762,483 | *GNA12* | A/G | 0.035 | 0.004 | **3.58E-20** | 210,942 |  | 0.032 | 0.005 | **1.40E-10** | 117,217 |  | 0.040 | 0.006 | **7.01E-13** | 93,911 | 1.049 | 0.306 | 4.7% |
| HIP | rs10950949 | 7 | 23,485,785 | *IGF2BP3* | A/C | 0.021 | 0.004 | **1.52E-08** | 201,716 |  | 0.027 | 0.005 | **1.28E-08** | 113,666 |  | 0.014 | 0.006 | 0.009 | 88,236 | 2.770 | 0.096 | 63.9% |
| HIP | rs849141 | 7 | 28,151,616 | *JAZF1* | A/G | 0.032 | 0.004 | **1.64E-16** | 211,081 |  | 0.036 | 0.005 | **7.45E-13** | 117,321 |  | 0.028 | 0.006 | 5.23E-07 | 93,946 | 1.049 | 0.306 | 4.7% |
| HIP | rs42235 | 7 | 92,086,012 | *CDK6* | T/C | 0.036 | 0.004 | **8.39E-20** | 208,455 |  | 0.037 | 0.005 | **2.02E-13** | 114,861 |  | 0.033 | 0.006 | **2.30E-09** | 93,780 | 0.262 | 0.609 | 0.0% |
| HIP | rs13241538 | 7 | 130,090,402 | *KLF14* | C/G | 0.017 | 0.004 | 1.65E-06 | 210,935 |  | 0.033 | 0.005 | **9.89E-14** | 117,210 |  | -0.003 | 0.005 | 0.497 | 93,911 | 25.920 | **3.56E-07** | 96.1% |
| HIP | rs7008867 | 8 | 23,439,149 | *SLC25A37* | A/G | 0.024 | 0.004 | **6.01E-09** | 211,066 |  | 0.026 | 0.005 | 5.36E-07 | 117,318 |  | 0.021 | 0.006 | 3.05E-04 | 93,934 | 0.410 | 0.522 | 0.0% |
| HIP | rs10958476 | 8 | 57,258,362 | *PLAG1* | C/T | 0.028 | 0.005 | **8.81E-10** | 199,716 |  | 0.022 | 0.006 | 1.81E-04 | 111,294 |  | 0.035 | 0.007 | 2.21E-07 | 88,607 | 1.988 | 0.159 | 49.7% |
| HIP | rs6470764 | 8 | 130,794,847 | *GSDMC* | C/T | 0.039 | 0.005 | **8.38E-18** | 210,864 |  | 0.035 | 0.006 | **1.84E-09** | 117,209 |  | 0.044 | 0.006 | **1.17E-11** | 93,841 | 1.125 | 0.289 | 11.1% |
| HIP | rs7007820 | 8 | 135,685,919 | *ZFAT* | A/G | 0.021 | 0.004 | **1.04E-08** | 211,016 |  | 0.023 | 0.005 | 4.42E-07 | 117,303 |  | 0.018 | 0.005 | 4.92E-04 | 93,899 | 0.500 | 0.480 | 0.0% |
| HIP | rs473902 | 9 | 97,296,056 | *PTCH1* | T/G | 0.058 | 0.008 | **6.60E-15** | 184,792 |  | 0.058 | 0.010 | **2.19E-09** | 102,592 |  | 0.059 | 0.011 | **2.44E-08** | 82,387 | 0.005 | 0.946 | 0.0% |
| HIP | rs10123368 | 9 | 98,151,205 | *SLC35D2* | C/T | 0.026 | 0.004 | **5.00E-09** | 210,933 |  | 0.019 | 0.006 | 6.80E-04 | 117,265 |  | 0.036 | 0.006 | **2.07E-08** | 93,854 | 4.014 | 0.045 | 75.1% |
| HIP | rs7044106 | 9 | 122,533,883 | *C5-FBXW2* | C/G | 0.023 | 0.006 | 4.10E-05 | 143,412 |  | 0.039 | 0.007 | **5.73E-09** | 86,733 |  | -0.003 | 0.008 | 0.687 | 56,865 | 15.611 | **7.78E-05** | 93.6% |
| HIP | rs11231694 | 11 | 63,619,458 | *MACROD1* | G/C | 0.046 | 0.010 | 7.39E-06 | 131,983 |  | 0.070 | 0.013 | **4.33E-08** | 80,519 |  | 0.012 | 0.015 | 0.436 | 51,651 | 8.538 | 0.003 | 88.3% |
| HIP | rs686320 | 11 | 65,001,114 | *MALAT1* | G/C | 0.038 | 0.006 | **6.83E-12** | 199,308 |  | 0.038 | 0.007 | 6.80E-08 | 110,930 |  | 0.040 | 0.008 | 6.30E-07 | 88,564 | 0.035 | 0.851 | 0.0% |
| HIP | rs606452 | 11 | 74,953,826 | *SERPINH1* | A/C | 0.029 | 0.005 | **9.75E-09** | 210,552 |  | 0.028 | 0.007 | 2.16E-05 | 117,011 |  | 0.032 | 0.007 | 1.64E-05 | 93,727 | 0.163 | 0.686 | 0.0% |
| HIP | rs11612228 | 12 | 447,245 | *B4GALNT3* | T/C | 0.023 | 0.004 | **2.78E-08** | 181,613 |  | 0.016 | 0.005 | 0.002 | 102,084 |  | 0.031 | 0.006 | 1.47E-07 | 79,714 | 3.689 | 0.055 | 72.9% |
| HIP | rs2638953 | 12 | 28,425,682 | *CCDC91* | C/G | 0.024 | 0.004 | **2.04E-10** | 207,847 |  | 0.022 | 0.005 | 3.94E-06 | 115,827 |  | 0.027 | 0.006 | 6.17E-07 | 92,206 | 0.410 | 0.522 | 0.0% |
| HIP | rs1351394 | 12 | 64,638,093 | *HMGA2* | T/C | 0.025 | 0.004 | **4.94E-13** | 210,068 |  | 0.022 | 0.005 | 7.59E-07 | 116,398 |  | 0.028 | 0.005 | **2.08E-08** | 93,856 | 0.720 | 0.396 | 0.0% |
| HIP | rs10748128 | 12 | 68,113,925 | *FRS2* | T/G | 0.023 | 0.004 | **3.96E-09** | 197,305 |  | 0.019 | 0.005 | 2.01E-04 | 109,831 |  | 0.028 | 0.006 | 9.41E-07 | 87,660 | 1.328 | 0.249 | 24.7% |
| HIP | rs12817549 | 12 | 92,645,445 | *CRADD* | T/C | 0.029 | 0.004 | **2.22E-16** | 210,856 |  | 0.031 | 0.005 | **1.50E-11** | 117,196 |  | 0.029 | 0.005 | **1.71E-08** | 93,847 | 0.080 | 0.777 | 0.0% |
| HIP | rs1727294 | 12 | 122,182,467 | *PITPNM2* | A/G | 0.032 | 0.004 | **8.27E-14** | 208,707 |  | 0.041 | 0.006 | **6.19E-14** | 115,024 |  | 0.020 | 0.006 | 0.001 | 93,869 | 6.125 | 0.013 | 83.7% |
| HIP | rs1809889 | 12 | 123,367,179 | *FAM101A* | T/C | 0.023 | 0.004 | **1.57E-08** | 208,493 |  | 0.022 | 0.005 | 3.10E-05 | 115,944 |  | 0.025 | 0.006 | 1.76E-05 | 92,735 | 0.148 | 0.701 | 0.0% |
| HIP | rs558003 | 13 | 50,070,964 | *DLEU7* | A/G | 0.049 | 0.006 | **3.83E-15** | 199,267 |  | 0.053 | 0.008 | **5.22E-11** | 110,894 |  | 0.046 | 0.009 | 3.12E-07 | 88,559 | 0.338 | 0.561 | 0.0% |
| HIP | rs10140922 | 14 | 34,899,865 | *NFKBIA* | G/T | 0.030 | 0.005 | **4.57E-11** | 143,568 |  | 0.034 | 0.006 | **2.99E-09** | 86,815 |  | 0.024 | 0.007 | 5.90E-04 | 56,940 | 1.176 | 0.278 | 15.0% |
| HIP | rs1254257 | 14 | 59,901,932 | *PPM1A* | C/T | 0.029 | 0.005 | **9.06E-10** | 143,808 |  | 0.034 | 0.006 | **7.46E-09** | 86,955 |  | 0.021 | 0.007 | 0.003 | 57,040 | 1.988 | 0.159 | 49.7% |
| HIP | rs7162542 | 15 | 82,305,294 | *ADAMTSL3* | G/C | 0.041 | 0.004 | **1.79E-30** | 209,929 |  | 0.043 | 0.005 | **1.19E-20** | 116,556 |  | 0.038 | 0.005 | **5.36E-14** | 93,559 | 0.500 | 0.480 | 0.0% |
| HIP | rs16942341 | 15 | 87,189,909 | *ACAN* | C/T | 0.066 | 0.012 | **3.83E-08** | 192,440 |  | 0.061 | 0.015 | 6.88E-05 | 106,755 |  | 0.075 | 0.017 | 1.63E-05 | 85,661 | 0.381 | 0.537 | 0.0% |
| HIP | rs4246307 | 15 | 98,581,347 | *ADAMTS17* | G/A | 0.024 | 0.004 | **4.25E-08** | 143,549 |  | 0.018 | 0.005 | 7.45E-04 | 86,811 |  | 0.032 | 0.007 | 1.16E-06 | 56,924 | 2.649 | 0.104 | 62.2% |
| HIP | rs3747579 | 16 | 4,385,328 | *CORO7* | T/C | 0.023 | 0.004 | **6.61E-09** | 210,715 |  | 0.023 | 0.005 | 5.51E-06 | 117,131 |  | 0.023 | 0.006 | 5.48E-05 | 93,770 | 0.000 | 1.000 | 0.0% |
| HIP | rs7187776 | 16 | 28,765,146 | *TUFM* | G/A | 0.020 | 0.004 | **3.97E-08** | 210,057 |  | 0.025 | 0.005 | 7.12E-08 | 117,208 |  | 0.013 | 0.005 | 0.009 | 93,034 | 2.880 | 0.090 | 65.3% |
| HIP | rs17193922 | 16 | 52,102,131 | *AKTIP* | G/C | 0.024 | 0.004 | **1.44E-09** | 198,589 |  | 0.025 | 0.005 | 1.03E-06 | 110,555 |  | 0.022 | 0.006 | 2.11E-04 | 88,220 | 0.148 | 0.701 | 0.0% |
| HIP | rs2377058 | 16 | 88,262,332 | *C16orf55* | G/A | 0.023 | 0.004 | **6.88E-10** | 210,642 |  | 0.021 | 0.005 | 1.26E-05 | 117,110 |  | 0.027 | 0.005 | 8.52E-07 | 93,717 | 0.720 | 0.396 | 0.0% |
| HIP | rs2034088 | 17 | 369,801 | *VPF53-FAM101B* | T/C | 0.021 | 0.004 | **4.80E-09** | 210,737 |  | 0.028 | 0.005 | **9.63E-10** | 117,142 |  | 0.014 | 0.005 | 0.007 | 93,781 | 3.920 | 0.048 | 74.5% |
| HIP | rs9890032 | 17 | 26,190,060 | *ATAD5* | C/G | 0.026 | 0.004 | **2.04E-12** | 207,385 |  | 0.022 | 0.005 | 2.61E-06 | 115,524 |  | 0.030 | 0.005 | **8.02E-09** | 92,047 | 1.280 | 0.258 | 21.9% |
| HIP | rs561341 | 17 | 27,340,498 | *SUZ12* | G/T | 0.031 | 0.005 | **3.30E-10** | 211,106 |  | 0.027 | 0.006 | 2.45E-05 | 117,333 |  | 0.036 | 0.007 | 8.48E-07 | 93,959 | 0.953 | 0.329 | 0.0% |
| HIP | rs13695 | 17 | 35,798,719 | *TOP2A* | T/C | 0.027 | 0.004 | **9.52E-10** | 190,010 |  | 0.029 | 0.006 | 3.11E-07 | 106,768 |  | 0.026 | 0.007 | 7.55E-05 | 83,428 | 0.106 | 0.745 | 0.0% |
| HIP | rs7223966 | 17 | 59,247,130 | *DDX42* | A/G | 0.029 | 0.004 | **1.68E-13** | 211,080 |  | 0.024 | 0.005 | 1.40E-06 | 117,323 |  | 0.035 | 0.006 | **8.45E-10** | 93,943 | 1.984 | 0.159 | 49.6% |
| HIP | rs1396517 | 17 | 65,933,398 | *KCNJ2* | C/T | 0.021 | 0.004 | **3.64E-09** | 211,031 |  | 0.028 | 0.005 | **3.43E-10** | 117,299 |  | 0.012 | 0.005 | 0.017 | 93,919 | 5.120 | 0.024 | 80.5% |
| HIP | rs4369779 | 18 | 18,989,406 | *CABLES1* | C/T | 0.035 | 0.004 | **3.33E-15** | 210,787 |  | 0.036 | 0.006 | **5.87E-11** | 117,164 |  | 0.033 | 0.006 | 1.78E-07 | 93,809 | 0.125 | 0.724 | 0.0% |
| HIP | rs181553 | 18 | 44,816,028 | *DYM* | A/G | 0.029 | 0.004 | **8.93E-15** | 210,832 |  | 0.031 | 0.005 | **3.31E-10** | 117,206 |  | 0.028 | 0.005 | 2.76E-07 | 93,812 | 0.180 | 0.671 | 0.0% |
| HIP | rs12980348 | 19 | 2,132,607 | *DOT1L* | G/T | 0.029 | 0.004 | **8.81E-16** | 210,456 |  | 0.022 | 0.005 | 1.90E-06 | 116,999 |  | 0.038 | 0.005 | **2.70E-13** | 93,642 | 5.120 | 0.024 | 80.5% |
| HIP | rs891088 | 19 | 7,135,762 | *INSR* | G/A | 0.026 | 0.004 | **1.04E-10** | 210,109 |  | 0.027 | 0.005 | 3.08E-07 | 116,817 |  | 0.026 | 0.006 | 6.92E-06 | 93,478 | 0.016 | 0.898 | 0.0% |
| HIP | rs2326788 | 20 | 6,418,094 | *BMP2* | G/A | 0.022 | 0.004 | **1.48E-09** | 210,499 |  | 0.021 | 0.005 | 9.97E-06 | 116,990 |  | 0.023 | 0.005 | 1.16E-05 | 93,695 | 0.080 | 0.777 | 0.0% |
| HIP | rs169797 | 20 | 31,390,151 | *CDK5RAP1* | A/G | 0.024 | 0.004 | **9.72E-10** | 204,594 |  | 0.021 | 0.005 | 4.05E-05 | 114,641 |  | 0.030 | 0.006 | 1.76E-07 | 90,139 | 1.328 | 0.249 | 24.7% |
| HIP | rs6088619 | 20 | 32,875,532 | *NCOA6* | G/A | 0.039 | 0.005 | **9.31E-13** | 199,166 |  | 0.038 | 0.007 | 7.74E-08 | 110,812 |  | 0.041 | 0.008 | 1.88E-07 | 88,539 | 0.080 | 0.778 | 0.0% |
| HIP | rs143384 | 20 | 33,489,170 | *GDF5* | G/A | 0.044 | 0.004 | **1.12E-31** | 209,682 |  | 0.040 | 0.005 | **2.91E-16** | 116,552 |  | 0.051 | 0.005 | **4.72E-21** | 93,316 | 2.420 | 0.120 | 58.7% |
| HIP | rs6060717 | 20 | 34,008,061 | *SCAND1* | C/T | 0.032 | 0.005 | **6.25E-12** | 211,073 |  | 0.031 | 0.006 | 1.48E-07 | 117,314 |  | 0.033 | 0.007 | 7.76E-07 | 93,945 | 0.047 | 0.828 | 0.0% |
| HIP | rs1053593 | 22 | 33,990,875 | *HMGXB4* | T/G | 0.021 | 0.004 | **3.90E-08** | 202,070 |  | 0.029 | 0.005 | **1.76E-09** | 114,347 |  | 0.011 | 0.005 | 0.051 | 87,908 | 6.480 | 0.011 | 84.6% |

SNP, single nucleotide polymorphism; CHR, chromosome; POS, position; A1, effect allele, is the trait-increasing allele; A2, alternative allele; BETA, SNP effect size; SE, standard error of the SNP effect size; *P* and *N* represent *P* value and sample size, respectively. Note, also given are the Cochran’s Q statistic, *P*_het_ value and I^2^ statistic. Genome-wide significant SNPs of the anthropometric traits (*P*<5.00E-8) and the *P*_het_ values significant at the Bonferroni threshold of 0.05/*k* (*k* is the number of instrumental variables for a given anthropometric trait) are marked in bold.

**Supplementary Table 4** Association of anthropometric trait with the risk of breast cancer using sex-combined instruments and after removing instruments with sex heterogeneity

| exposure | sex-combined IVs | |  | remove IVs with sex heterogeneity | |
| --- | --- | --- | --- | --- | --- |
|  | *k*_0_/*k*_1_ | OR (95%CI, *P*) |  | *k*_0_/*k*_1_ | OR (95%CI, *P*) |
| BMI | 97/92 | 0.845 (0.756~0.945, 0.003) |  | 95/90 | 0.849 (0.759~0.950, 0.004) |
| WHR | 39/38 | 1.002 (0.841~1.194, 0.984) |  | 30/29 | 1.008 (0.821~1.237, 0.942) |
| WC | 70/66 | 0.866 (0.767~0.978, 0.020) |  | 65/61 | 0.889 (0.784~1.009, 0.069) |
| HIP | 89/86 | 0.988 (0.885~1.102, 0.823) |  | 88/85 | 0.997 (0.892~1.114, 0.956) |

IV, instrumental variable; BMI, body mass index; WHR, waist-to-hip ratio; WC, waist circumference; HIP, hip circumference; OR, odds ratio; CI, confidence internal. Note, *k*_0_ is the number of candidate instruments, while *k*_1_ is the final number of instruments employed in the analysis.

**Supplementary Table 5** Association of anthropometric trait with the risk of prostate cancer using sex-combined instruments and after removing instruments with sex heterogeneity

| exposure | sex-combined IVs | |  | remove IVs with sex heterogeneity | |
| --- | --- | --- | --- | --- | --- |
|  | *k*_0_/*k*_1_ | OR (95%CI, *P*) |  | *k*_0_/*k*_1_ | OR (95%CI, *P*) |
| BMI | 97/60 | 0.865 (0.764~0.979, 0.022) |  | 95/58 | 0.847 (0.746~0.963, 0.011) |
| WHR | 39/21 | 0.854 (0.681~1.071, 0.172) |  | 30/17 | 0.856 (0.655~1.117, 0.252) |
| WC | 70/50 | 0.954 (0.808~1.126, 0.577) |  | 65/47 | 0.953 (0.800~1.135, 0.589) |
| HIP | 89/64 | 1.016 (0.886~1.166, 0.817) |  | 88/64 | 1.016 (0.886~1.166, 0.817) |

IV, instrumental variable; BMI, body mass index; WHR, waist-to-hip ratio; WC, waist circumference; HIP, hip circumference; OR, odds ratio; CI, confidence internal. Note, *k*_0_ is the number of candidate instruments, while *k*_1_ is the final number of instruments employed in the analysis.


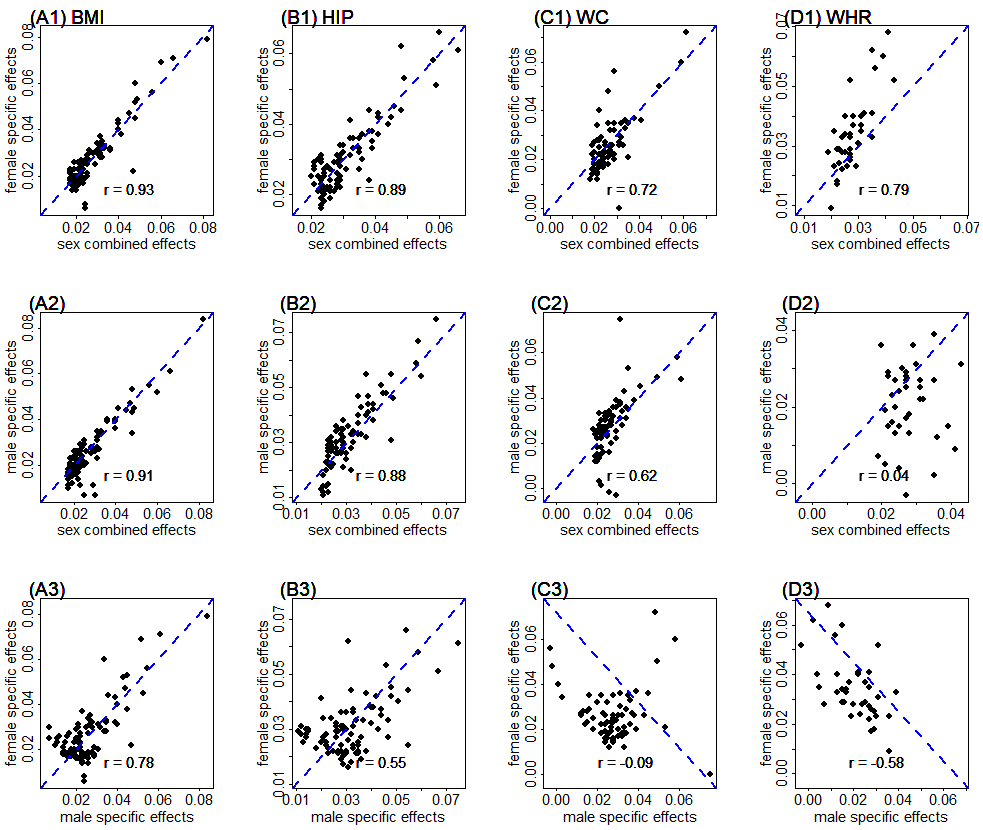


**Supplementary Figure 1** Pearson’s correlation *r_g_* between the sex-combined and sex-specific instruments as well as between the male-specific and female-specific instruments for four anthropometric traits. The estimated correlation coefficients are typically larger than zero but less than one except WC in (C3) with $\hat{r}_{g}$=-0.09 and WHR in (D3) with $\hat{r}_{g}$=-0.58.


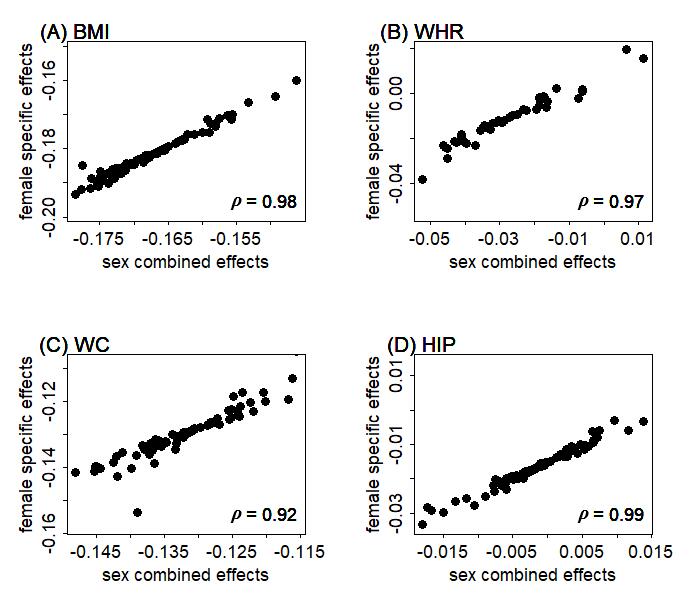


**Supplementary Figure 2** Correlation between the leave-one-out causal effect obtained with sex-combined instrumental variables and that yielded with female-specific instrumental variables of each anthropometric trait for breast cancer. The leave-one-out causal effects were estimated by omitting one SNP at a time; a total of 97 SNPs for BMI, 48 SNPs for WHR, 76 SNPs for WC and 95 SNPs for HIP were included into the analysis (see Supplementary Table S3 for details).


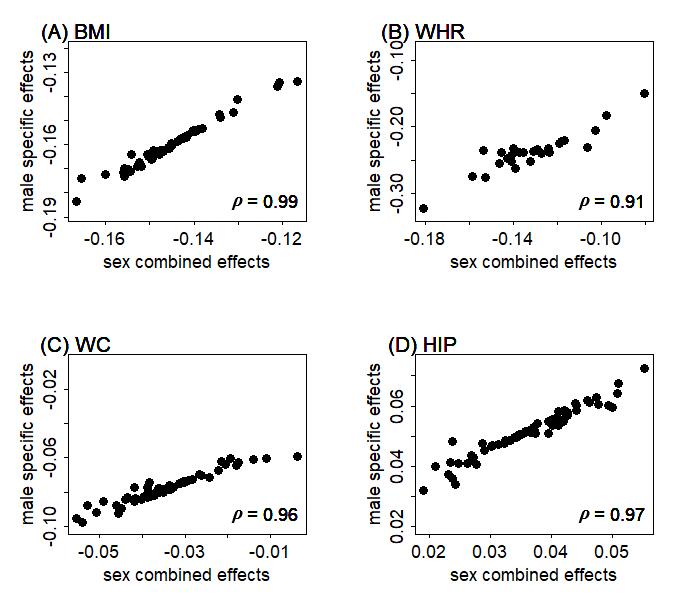


**Supplementary Figure 3** Correlation between the leave-one-out causal effect obtained with sex-combined instrumental variables and that yielded with male-specific instrumental variables of each anthropometric trait for prostate cancer. The leave-one-out causal effects were estimated by omitting one SNP at a time; a total of 97 SNPs for BMI, 48 SNPs for WHR, 76 SNPs for WC and 95 SNPs for HIP were included into the analysis (see Supplementary Table S3 for details).


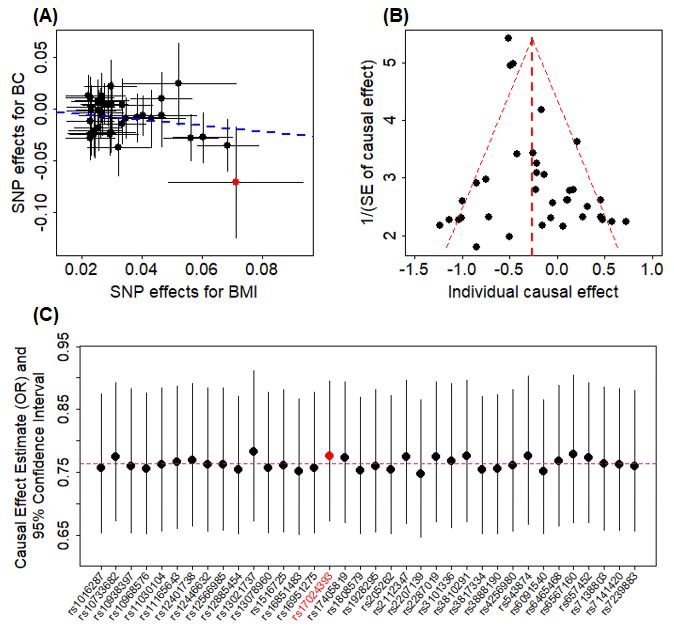


**Supplementary Figure 4** Scatter, funnel, and forest plots. (A) Relationship between the SNP effect sizes of BMI (x-axis) and the corresponding effect sizes of breast cancer (y-axis); in the plot horizontal/vertical lines represent the 95% confidence interval; the line in blue represents the estimated causal effect with the IVW method; the red dot is identified as a potential outlier (i.e. rs17024393 on gene *GNAT2* with the large effect size of 0.071 on BMI); (B) Funnel plot for individual causal effects of BMI on breast cancer; the horizontal dot line denotes the overall estimated causal effect with IVW method; (C) Estimated causal effects and 95% confidence interval for BMI on breast cancer in the leave-one-out analysis by omitting one SNP (x-axis) at a time; estimation is carried out using the random-effects inverse-variance weighted method; in the plot the potential instrumental outlier (i.e. rs17024393) is highlighted in red; The horizontal line represents the causal effect estimate using all instruments together.

**References**

1. Locke AE, Kahali B, Berndt SI, Justice AE, Pers TH, Felix R, et al. Genetic studies of body mass index yield new insights for obesity biology. Nature. 2015; 518: 197-U401.

2. Guo Q, Burgess S, Turman C, Bolla MK, Wang Q, Lush M, et al. Body mass index and breast cancer survival: a Mendelian randomization analysis. Int J Epidemiol. 2017; 46: 1814-22.

3. Qian F, Wang S, Mitchell J, McGuffog L, Barrowdale D, Leslie G, et al. Height and Body Mass Index as Modifiers of Breast Cancer Risk in BRCA1/2 Mutation Carriers: A Mendelian Randomization Study. J Natl Cancer Inst. 2019; 111: 350-64.

4. Ooi BNS, Loh H, Ho PJ, Milne RL, Giles G, Gao C, et al. The genetic interplay between body mass index, breast size and breast cancer risk: a Mendelian randomization analysis. Int J Epidemiol. 2019.

5. Turcot V, Lu YC, Highland HM, Schurmann C, Justice AE, Fine RS, et al. Protein-altering variants associated with body mass index implicate pathways that control energy intake and expenditure in obesity (vol 50, pg 26, 2017). Nat Genet. 2019; 51: 1191-2.

6. Shu X, Wu L, Khankari NK, Shu XO, Wang TJ, Michailidou K, et al. Associations of obesity and circulating insulin and glucose with breast cancer risk: a Mendelian randomization analysis. Int J Epidemiol. 2018.

7. Felix JF, Bradfield JP, Monnereau C, van der Valk RJP, Stergiakouli E, Chesi A, et al. Genome-wide association analysis identifies three new susceptibility loci for childhood body mass index. Hum Mol Genet. 2016; 25: 389-403.

8. Gao C, Patel CJ, Michailidou K, Peters U, Gong J, Schildkraut J, et al. Mendelian randomization study of adiposity-related traits and risk of breast, ovarian, prostate, lung and colorectal cancer. Int J Epidemiol. 2016; 45: 896-908.

9. Hindorff LA, Sethupathy P, Junkins HA, Ramos EM, Mehta JP, Collins FS, et al. Potential etiologic and functional implications of genome-wide association loci for human diseases and traits. Proc Natl Acad Sci U S A. 2009; 106: 9362-7.

10. Guo Y, Andersen SW, Shu XO, Michailidou K, Bolla MK, Wang Q, et al. Genetically Predicted Body Mass Index and Breast Cancer Risk: Mendelian Randomization Analyses of Data from 145,000 Women of European Descent. PLoS Med. 2016; 13.

11. Speliotes EK, Willer CJ, Berndt SI, Monda KL, Thorleifsson G, Jackson AU, et al. Association analyses of 249,796 individuals reveal 18 new loci associated with body mass index. Nat Genet. 2010; 42: 937-U53.

12. Benn M, Tybjaerg-Hansen A, Smith GD, Nordestgaard BG. High body mass index and cancer risk-a Mendelian randomisation study. Eur J Epidemiol. 2016; 31: 879-92.

13. Gudbjartsson DF, Walters GB, Thorleifsson G, Stefansson H, Halldorsson BV, Zusmanovich P, et al. Many sequence variants affecting diversity of adult human height. Nat Genet. 2008; 40: 609-15.

14. Weedon MN, Lettre G, Freathy RM, Lindgren CM, Voight BF, Perry JRB, et al. A common variant of HMGA2 is associated with adult and childhood height in the general population. Nat Genet. 2007; 39: 1245-50.

15. Sanna S, Jackson AU, Nagaraja R, Willer CJ, Chen WM, Bonnycastle LL, et al. Common variants in the GDF5-UQCC region are associated with variation in human height. Nat Genet. 2008; 40: 198-203.

16. Lettre G, Jackson AU, Gieger C, Schumacher FR, Berndt SI, Sanna S, et al. Identification of ten loci associated with height highlights new biological pathways in human growth. Nat Genet. 2008; 40: 584-91.

17. Weedon MN, Lango H, Lindgren CM, Wallace C, Evans DM, Mangino M, et al. Genome-wide association analysis identifies 20 loci that influence adult height. Nat Genet. 2008; 40: 575-83.

18. Lango Allen H, Estrada K, Lettre G, Berndt SI, Weedon MN, Rivadeneira F, et al. Hundreds of variants clustered in genomic loci and biological pathways affect human height. Nature. 2010; 467: 832-8.

19. Zhang B, Shu XO, Delahanty RJ, Zeng CJ, Michailidou K, Bolla MK, et al. Height and Breast Cancer Risk: Evidence From Prospective Studies and Mendelian Randomization. Jnci-Journal of the National Cancer Institute. 2015; 107.

20. Wood AR, Esko T, Yang J, Vedantam S, Pers TH, Gustafsson S, et al. Defining the role of common variation in the genomic and biological architecture of adult human height. Nat Genet. 2014; 46: 1173-86.

21. Khankari NK, Shu XO, Wen WQ, Kraft P, Lindstrom S, Peters U, et al. Association between Adult Height and Risk of Colorectal, Lung, and Prostate Cancer: Results from Meta-analyses of Prospective Studies and Mendelian Randomization Analyses. PLoS Med. 2016; 13.

22. Lai FY, Nath M, Hamby SE, Thompson JR, Nelson CP, Samani NJ. Adult height and risk of 50 diseases: a combined epidemiological and genetic analysis. BMC Med. 2018; 16: 187.

23. Teslovich TM, Musunuru K, Smith AV, Edmondson AC, Stylianou IM, Koseki M, et al. Biological, clinical and population relevance of 95 loci for blood lipids. Nature. 2010; 466: 707-13.

24. Orho-Melander M, Hindy G, Borgquist S, Schulz CA, Manjer J, Melander O, et al. Blood lipid genetic scores, the HMGCR gene and cancer risk: a Mendelian randomization study. Int J Epidemiol. 2018; 47: 495-505.

25. Willer CJ, Schmidt EM, Sengupta S, Peloso GM, Gustafsson S, Kanoni S, et al. Discovery and refinement of loci associated with lipid levels. Nat Genet. 2013; 45: 1274-83.

26. Beeghly-Fadiel A, Khankari NK, Delahanty RJ, Shu XO, Lu Y, Schmidt MK, et al. A Mendelian randomization analysis of circulating lipid traits and breast cancer risk. Int J Epidemiol. 2019.

27. Nowak C, Arnlov J. A Mendelian randomization study of the effects of blood lipids on breast cancer risk. Nat Commun. 2018; 9: 3957.

28. Klarin D, Damrauer SM, Cho K, Sun YV, Teslovich TM, Honerlaw J, et al. Genetics of blood lipids among ~300,000 multi-ethnic participants of the Million Veteran Program. Nat Genet. 2018; 50: 1514-23.

29. Johnson KE, Siewert KM, Klarin D, Damrauer SM, Chang KM, Tsao PS, et al. The relationship between circulating lipids and breast cancer risk: A Mendelian randomization study. PLoS Med. 2020; 17: e1003302.

30. Horikoshi M, Yaghootkar H, Mook-Kanamori DO, Sovio U, Taal HR, Hennig BJ, et al. New loci associated with birth weight identify genetic links between intrauterine growth and adult height and metabolism. Nat Genet. 2013; 45: 76-U115.

31. Horikoshi M, Beaumont RN, Day FR, Warrington NM, Kooijman MN, Fernandez-Tajes J, et al. Genome-wide associations for birth weight and correlations with adult disease. Nature. 2016; 538: 248-+.

32. Kar SP, Andrulis IL, Brenner H, Burgess S, Chang-Claude J, Considine D, et al. The association between weight at birth and breast cancer risk revisited using Mendelian randomisation. Eur J Epidemiol. 2018; 34: 591-600.

33. Heid IM, Jackson AU, Randall JC, Winkler TW, Qi L, Steinthorsdottir V, et al. Meta-analysis identifies 13 new loci associated with waist-hip ratio and reveals sexual dimorphism in the genetic basis of fat distribution. Nat Genet. 2011; 43: 1164-.

34. Elsworth BL, Mitchell RE, Raistrick CA. MRC IEU UK Biobank GWAS pipeline version 2. 2019.

35. Richardson TG, Sanderson E, Elsworth B, Tilling K, Davey Smith G. Use of genetic variation to separate the effects of early and later life adiposity on disease risk: mendelian randomisation study. BMJ. 2020; 369: m1203.

36. Day FR, Thompson DJ, Helgason H, Chasman DI, Finucane H, Sulem P, et al. Genomic analyses identify hundreds of variants associated with age at menarche and support a role for puberty timing in cancer risk. Nat Genet. 2017; 49: 834-+.

37. Burgess S, Thompson DJ, Rees JMB, Day FR, Perry JR, Ong KK. Dissecting Causal Pathways Using Mendelian Randomization with Summarized Genetic Data: Application to Age at Menarche and Risk of Breast Cancer. Genetics. 2017; 207: 481-7.

38. Perry JRB, Day F, Elks CE, Sulem P, Thompson DJ, Ferreira T, et al. Parent-of-origin-specific allelic associations among 106 genomic loci for age at menarche. Nature. 2014; 514: 92-+.

39. Lunetta KL, Day FR, Sulem P, Ruth KS, Tung JY, Hinds DA, et al. Rare coding variants and X-linked loci associated with age at menarche. Nat Commun. 2015; 6.

40. Anderson D, Holt BJ, Pennell CE, Holt PG, Hart PH, Blackwell JM. Genome-wide association study of vitamin D levels in children: replication in the Western Australian Pregnancy Cohort (Raine) study. Genes Immun. 2014; 15: 578-83.

41. Cheng WW, Wang ZK, Shangguan HF, Zhu Q, Zhang HY. Are vitamins relevant to cancer risks? A Mendelian randomization investigation. Nutrition. 2020; 78: 110870.

42. Ahn J, Yu K, Stolzenberg-Solomon R, Simon KC, McCullough ML, Gallicchio L, et al. Genome-wide association study of circulating vitamin D levels. Hum Mol Genet. 2010; 19: 2739-45.

43. Wang TJ, Zhang F, Richards JB, Kestenbaum B, van Meurs JB, Berry D, et al. Common genetic determinants of vitamin D insufficiency: a genome-wide association study. Lancet. 2010; 376: 180-8.

44. Dimitrakopoulou VI, Tsilidis KK, Haycock PC, Dimou NL, Al-Dabhani K, Martin RM, et al. Circulating vitamin D concentration and risk of seven cancers: Mendelian randomisation study. BMJ. 2017; 359: j4761.

45. Jiang X, O'Reilly PF, Aschard H, Hsu YH, Richards JB, Dupuis J, et al. Genome-wide association study in 79,366 European-ancestry individuals informs the genetic architecture of 25-hydroxyvitamin D levels. Nat Commun. 2018; 9: 260.

46. Jiang X, Dimou NL, Al-Dabhani K, Lewis SJ, Martin RM, Haycock PC, et al. Circulating vitamin D concentrations and risk of breast and prostate cancer: a Mendelian randomization study. Int J Epidemiol. 2018.

47. Hiraki LT, Major JM, Chen C, Cornelis MC, Hunter DJ, Rimm EB, et al. Exploring the genetic architecture of circulating 25-hydroxyvitamin D. Genet Epidemiol. 2013; 37: 92-8.

48. Chandler PD, Tobias DK, Wang L, Smith-Warner SA, Chasman DI, Rose L, et al. Association between Vitamin D Genetic Risk Score and Cancer Risk in a Large Cohort of US Women. Nutrients. 2018; 10.

49. Manousaki D, Paternoster L, Standl M, Moffatt MF, Farrall M, Bouzigon E, et al. Vitamin D levels and susceptibility to asthma, elevated immunoglobulin E levels, and atopic dermatitis: A Mendelian randomization study. PLoS Med. 2017; 14: e1002294.

50. Wang S, Huo D, Kupfer S, Alleyne D, Ogundiran TO, Ojengbede O, et al. Genetic variation in the vitamin D related pathway and breast cancer risk in women of African ancestry in the root consortium. Int J Cancer. 2018; 142: 36-43.

51. Vimaleswaran KS, Cavadino A, Berry DJ, Genetic Investigation of Anthropometric Traits C, Whittaker JC, Power C, et al. Genetic association analysis of vitamin D pathway with obesity traits. Int J Obes (Lond). 2013; 37: 1399-406.

52. Ong JS, Cuellar-Partida G, Lu Y, Australian Ovarian Cancer S, Fasching PA, Hein A, et al. Association of vitamin D levels and risk of ovarian cancer: a Mendelian randomization study. Int J Epidemiol. 2016; 45: 1619-30.

53. Taylor AE, Burgess S, Ware JJ, Gage SH, Richards JB, Smith GD, et al. Investigating causality in the association between 25(OH)D and schizophrenia. Sci Rep. 2016; 6.

54. Ong JS, Gharahkhani P, An JY, Law MH, Whiteman DC, Neale RE, et al. Vitamin D and overall cancer risk and cancer mortality: a Mendelian randomization study. Hum Mol Genet. 2018; 27: 4315-22.

55. Pooley KA, Bojesen SE, Weischer M, Nielsen SF, Thompson D, Al Olama AA, et al. A genome-wide association scan (GWAS) for mean telomere length within the COGS project: identified loci show little association with hormone-related cancer risk. Hum Mol Genet. 2013; 22: 5056-64.

56. Mangino M, Hwang SJ, Spector TD, Hunt SC, Kimura M, Fitzpatrick AL, et al. Genome-wide meta-analysis points to CTC1 and ZNF676 as genes regulating telomere homeostasis in humans. Hum Mol Genet. 2012; 21: 5385-94.

57. Gu JA, Chen M, Shete S, Amos CI, Kamat A, Ye YQ, et al. A Genome-Wide Association Study Identifies a Locus on Chromosome 14q21 as a Predictor of Leukocyte Telomere Length and as a Marker of Susceptibility for Bladder Cancer. Cancer Prev Res. 2011; 4: 514-21.

58. Codd V, Nelson CP, Albrecht E, Mangino M, Deelen J, Buxton JL, et al. Identification of seven loci affecting mean telomere length and their association with disease. Nat Genet. 2013; 45: 422-7, 7e1-2.

59. Codd V, Mangino M, van der Harst P, Braund PS, Kaiser M, Beveridge AJ, et al. Common variants near TERC are associated with mean telomere length. Nat Genet. 2010; 42: 197-9.

60. Levy D, Neuhausen SL, Hunt SC, Kimura M, Hwang SJ, Chen W, et al. Genome-wide association identifies OBFC1 as a locus involved in human leukocyte telomere biology. Proc Natl Acad Sci U S A. 2010; 107: 9293-8.

61. Haycock PC, Burgess S, Nounu A, Zheng J, Okoli GN, Bowden J, et al. Association Between Telomere Length and Risk of Cancer and Non-Neoplastic Diseases A Mendelian Randomization Study. Jama Oncol. 2017; 3: 636-51.

62. Zhang C, Doherty JA, Burgess S, Hung RJ, Lindstrom S, Kraft P, et al. Genetic determinants of telomere length and risk of common cancers: a Mendelian randomization study. Hum Mol Genet. 2015; 24: 5356-66.

63. Li C, Stoma S, Lotta LA, Warner S, Albrecht E, Allione A, et al. Genome-wide Association Analysis in Humans Links Nucleotide Metabolism to Leukocyte Telomere Length. Am J Hum Genet. 2020; 106: 389-404.

64. Gao Y, Wei Y, Zhou X, Huang S, Zhao H, Zeng P. Assessing the Relationship Between Leukocyte Telomere Length and Cancer Risk/Mortality in UK Biobank and TCGA Datasets With the Genetic Risk Score and Mendelian Randomization Approaches. Front Genet. 2020; 11: 583106.

65. Ripke S, Neale BM, Corvin A, Walters JTR, Farh KH, Holmans PA, et al. Biological insights from 108 schizophrenia-associated genetic loci. Nature. 2014; 511: 421-+.

66. Pardinas AF, Holmans P, Pocklington AJ, Escott-Price V, Ripke S, Carrera N, et al. Common schizophrenia alleles are enriched in mutation-intolerant genes and in regions under strong background selection. Nat Genet. 2018; 50: 381-+.

67. Shi J, Wu L, Zheng W, Wen W, Wang S, Shu X, et al. Genetic Evidence for the Association between Schizophrenia and Breast Cancer. J Psychiatr Brain Sci. 2018; 3.

68. Byrne EM, Ferreira MAR, Xue A, Lindstrom S, Jiang X, Yang J, et al. Is Schizophrenia a Risk Factor for Breast Cancer?-Evidence From Genetic Data. Schizophr Bull. 2019; 45: 1251-6.

69. Scott RA, Lagou V, Welch RP, Wheeler E, Montasser ME, Luan JA, et al. Large-scale association analyses identify new loci influencing glycemic traits and provide insight into the underlying biological pathways. Nat Genet. 2012; 44: 991-+.

70. Nead KT, Sharp SJ, Thompson DJ, Painter JN, Savage DB, Semple RK, et al. Evidence of a Causal Association Between Insulinemia and Endometrial Cancer: A Mendelian Randomization Analysis. Jnci-Journal of the National Cancer Institute. 2015; 107.

71. Wheeler E, Leong A, Liu CT, Hivert MF, Strawbridge RJ, Podmore C, et al. Impact of common genetic determinants of Hemoglobin A1c on type 2 diabetes risk and diagnosis in ancestrally diverse populations: A transethnic genome-wide meta-analysis. PLoS Med. 2017; 14: e1002383.

72. Au Yeung SL, Schooling CM. Impact of glycemic traits, type 2 diabetes and metformin use on breast and prostate cancer risk: a Mendelian randomization study. BMJ open diabetes research & care. 2019; 7: e000872.

73. Jung SY, Mancuso N, Yu H, Papp J, Sobei E, Zhang ZF. Genome-Wide Meta-analysis of Gene-Environmental Interaction for Insulin Resistance Phenotypes and Breast Cancer Risk in Postmenopausal Women. Cancer Prev Res. 2019; 12: 31-41.

74. Jung SY, Mancuso N, Papp J, Sobel E, Zhang ZF. Post genome-wide gene-environment interaction study: The effect of genetically driven insulin resistance on breast cancer risk using Mendelian randomization. PLoS One. 2019; 14: e0218917.

75. Murphy N, Knuppel A, Papadimitriou N, Martin RM, Tsilidis KK, Smith-Byrne K, et al. Insulin-like growth factor-1, insulin-like growth factor-binding protein-3, and breast cancer risk: observational and Mendelian randomization analyses with approximately 430 000 women. Ann Oncol. 2020.

76. Sinnott-Armstrong N, Tanigawa Y, Amar D, Mars NJ, Aguirre M, Venkataraman GR, et al. Genetics of 38 blood and urine biomarkers in the UK Biobank. 2019: 660506.

77. Larsson SC, Carter P, Vithayathil M, Kar S, Mason AM, Burgess S. Insulin-like growth factor-1 and site-specific cancers: A Mendelian randomization study. Cancer medicine. 2020; 9: 6836-42.

78. Teumer A, Qi Q, Nethander M, Aschard H, Bandinelli S, Beekman M, et al. Genomewide meta-analysis identifies loci associated with IGF-I and IGFBP-3 levels with impact on age-related traits. Aging Cell. 2016; 15: 811-24.

79. Benyamin B, Esko T, Ried JS, Radhakrishnan A, Vermeulen SH, Traglia M, et al. Novel loci affecting iron homeostasis and their effects in individuals at risk for hemochromatosis. Nat Commun. 2014; 5: 4926.

80. Yuan S, Carter P, Vithayathil M, Kar S, Giovannucci E, Mason AM, et al. Iron Status and Cancer Risk in UK Biobank: A Two-Sample Mendelian Randomization Study. Nutrients. 2020; 12.

81. Meyer TE, Verwoert GC, Hwang SJ, Glazer NL, Smith AV, van Rooij FJ, et al. Genome-wide association studies of serum magnesium, potassium, and sodium concentrations identify six Loci influencing serum magnesium levels. PLoS Genet. 2010; 6.

82. Papadimitriou N, Dimou N, Gill D, Tzoulaki I, Murphy N, Riboli E, et al. Genetically predicted circulating concentrations of micronutrients and risk of breast cancer: A Mendelian randomization study. Int J Cancer. 2021; 148: 646-53.

83. Swerdlow DI, Holmes MV, Kuchenbaecker KB, Engmann JE, Shah T, Sofat R, et al. The interleukin-6 receptor as a target for prevention of coronary heart disease: a mendelian randomisation analysis. Lancet. 2012; 379: 1214-24.

84. Robinson T, Martin RM, Yarmolinsky J. Mendelian randomisation analysis of circulating adipokines and C-reactive protein on breast cancer risk. Int J Cancer. 2020.

85. Dastani Z, Hivert MF, Timpson N, Perry JR, Yuan X, Scott RA, et al. Novel loci for adiponectin levels and their influence on type 2 diabetes and metabolic traits: a multi-ethnic meta-analysis of 45,891 individuals. PLoS Genet. 2012; 8: e1002607.

86. Huang J, Sabater-Lleal M, Asselbergs FW, Tregouet D, Shin SY, Ding J, et al. Genome-wide association study for circulating levels of PAI-1 provides novel insights into its regulation. Blood. 2012; 120: 4873-81.

87. Sun BB, Maranville JC, Peters JE, Stacey D, Staley JR, Blackshaw J, et al. Genomic atlas of the human plasma proteome. Nature. 2018; 558: 73-+.

88. Ligthart S, Vaez A, Vosa U, Stathopoulou MG, de Vries PS, Prins BP, et al. Genome Analyses of >200,000 Individuals Identify 58 Loci for Chronic Inflammation and Highlight Pathways that Link Inflammation and Complex Disorders. Am J Hum Genet. 2018; 103: 691-706.

89. Ahola-Olli AV, Würtz P, Havulinna AS, Aalto K, Pitkänen N, Lehtimäki T, et al. Genome-wide Association Study Identifies 27 Loci Influencing Concentrations of Circulating Cytokines and Growth Factors. Am J Hum Genet. 2017; 100: 40-50.

90. Li S, Xu Y, Zhang Y, Nie L, Ma Z, Ma L, et al. Mendelian randomization analyses of genetically predicted circulating levels of cytokines with risk of breast cancer. NPJ precision oncology. 2020; 4: 25.

91. Yuan S, Carter P, Bruzelius M, Vithayathil M, Kar S, Mason AM, et al. Effects of tumour necrosis factor on cardiovascular disease and cancer: A two-sample Mendelian randomization study. EBioMedicine. 2020; 59: 102956.

92. Guan W, Steffen BT, Lemaitre RN, Wu JHY, Tanaka T, Manichaikul A, et al. Genome-wide association study of plasma N6 polyunsaturated fatty acids within the cohorts for heart and aging research in genomic epidemiology consortium. Circ Cardiovasc Genet. 2014; 7: 321-31.

93. Larsson SC, Carter P, Vithayathil M, Mason AM, Michaëlsson K, Baron JA, et al. Genetically predicted plasma phospholipid arachidonic acid concentrations and 10 site-specific cancers in UK biobank and genetic consortia participants: A mendelian randomization study. Clin Nutr. 2020.

94. Teumer A, Chaker L, Groeneweg S, Li Y, Di Munno C, Barbieri C, et al. Genome-wide analyses identify a role for SLC17A4 and AADAT in thyroid hormone regulation. Nat Commun. 2018; 9: 4455.

95. Yuan S, Kar S, Vithayathil M, Carter P, Mason AM, Burgess S, et al. Causal associations of thyroid function and dysfunction with overall, breast and thyroid cancer: A two-sample Mendelian randomization study. Int J Cancer. 2020.

96. Li MY, Kwok MK, Fong SSM, Schooling CM. Indoleamine 2,3-dioxygenase and ischemic heart disease: a Mendelian Randomization study. Sci Rep. 2019; 9.

97. Jiang JY, Thalamuthu A, Ho JE, Mahajan A, Ek WE, Brown DA, et al. A Meta-Analysis of Genome-Wide Association Studies of Growth Differentiation Factor-15 Concentration in Blood. Frontiers in Genetics. 2018; 9.

98. Au Yeung SL, Luo S, Schooling CM. The impact of GDF-15, a biomarker for metformin, on the risk of coronary artery disease, breast and colorectal cancer, and type 2 diabetes and metabolic traits: a Mendelian randomisation study. Diabetologia. 2019; 62: 1638-46.

99. Sulem P, Gudbjartsson DF, Stacey SN, Helgason A, Rafnar T, Magnusson KP, et al. Genetic determinants of hair, eye and skin pigmentation in Europeans. Nat Genet. 2007; 39: 1443-52.

100. Dadd T, Stokowski RP, Pant PVK, Fereday A, Hinds DA, Jarman C, et al. A Genome-wide association study of skin pigmentation in a South Asian population. Genet Epidemiol. 2007; 31: 607-.

101. Kayser M, Liu F, Janssens ACJW, Rivadeneira F, Lao O, van Duijn K, et al. Three genome-wide association studies and a linkage analysis identify HERC2 as a human iris color gene. Am J Hum Genet. 2008; 82: 411-23.

102. Han J, Kraft P, Nan H, Guo Q, Chen C, Qureshi A, et al. A genome-wide association study identifies novel alleles associated with hair color and skin pigmentation. PLoS Genet. 2008; 4: e1000074.

103. Sulem P, Gudbjartsson DF, Stacey SN, Helgason A, Rafnar T, Jakobsdottir M, et al. Two newly identified genetic determinants of pigmentation in Europeans. Nat Genet. 2008; 40: 835-7.

104. Nan H, Kraft P, Qureshi AA, Guo Q, Chen C, Hankinson SE, et al. Genome-wide association study of tanning phenotype in a population of European ancestry. J Invest Dermatol. 2009; 129: 2250-7.

105. Liu F, Wollstein A, Hysi PG, Ankra-Badu GA, Spector TD, Park D, et al. Digital quantification of human eye color highlights genetic association of three new loci. PLoS Genet. 2010; 6: e1000934.

106. Eriksson N, Macpherson JM, Tung JY, Hon LS, Naughton B, Saxonov S, et al. Web-Based, Participant-Driven Studies Yield Novel Genetic Associations for Common Traits. PLoS Genet. 2010; 6.

107. Zhang MF, Song FJ, Liang LM, Nan HM, Zhang JW, Liu HL, et al. Genome-wide association studies identify several new loci associated with pigmentation traits and skin cancer risk in European Americans. Hum Mol Genet. 2013; 22: 2948-59.

108. Jacobs LC, Hamer MA, Gunn DA, Deelen J, Lall JS, van Heemst D, et al. A Genome-Wide Association Study Identifies the Skin Color Genes IRF4, MC1R, ASIP, and BNC2 Influencing Facial Pigmented Spots. J Invest Dermatol. 2015; 135: 1735-42.

109. Liu F, Visser M, Duffy DL, Hysi PG, Jacobs LC, Lao O, et al. Genetics of skin color variation in Europeans: genome-wide association studies with functional follow-up. Hum Genet. 2015; 134: 823-35.

110. Adhikari K, Fontanil T, Cal S, Mendoza-Revilla J, Fuentes-Guajardo M, Chacon-Duque JC, et al. A genome-wide association scan in admixed Latin Americans identifies loci influencing facial and scalp hair features. Nat Commun. 2016; 7: 10815.

111. Pickrell JK, Berisa T, Liu JZ, Segurel L, Tung JY, Hinds DA. Detection and interpretation of shared genetic influences on 42 human traits. Nat Genet. 2016; 48: 709-17.

112. Gomez-Acebo I, Dierssen-Sotos T, Palazuelos C, Fernandez-Navarro P, Castano-Vinyals G, Alonso-Molero J, et al. Pigmentation phototype and prostate and breast cancer in a select Spanish population-A Mendelian randomization analysis in the MCC-Spain study. PLoS One. 2018; 13.

113. Jones SE, Lane JM, Wood AR, van Hees VT, Tyrrell J, Beaumont RN, et al. Genome-wide association analyses of chronotype in 697,828 individuals provides insights into circadian rhythms. Nat Commun. 2019; 10: 343.

114. Richmond RC, Anderson EL, Dashti HS, Jones SE, Lane JM, Strand LB, et al. Investigating causal relations between sleep traits and risk of breast cancer in women: mendelian randomisation study. Bmj-British Medical Journal. 2019; 365.

115. Dashti HS, Jones SE, Wood AR, Lane JM, van Hees VT, Wang H, et al. Genome-wide association study identifies genetic loci for self-reported habitual sleep duration supported by accelerometer-derived estimates. Nat Commun. 2019; 10: 1100.

116. Lane JM, Jones SE, Dashti HS, Wood AR, Aragam KG, van Hees VT, et al. Biological and clinical insights from genetics of insomnia symptoms. Nat Genet. 2019; 51: 387-+.

117. Eriksson N, Benton GM, Do CB, Kiefer AK, Mountain JL, Hinds DA, et al. Genetic variants associated with breast size also influence breast cancer risk. BMC Med Genet. 2012; 13.

118. Ohlsson C, Wallaschofski H, Lunetta KL, Stolk L, Perry JR, Koster A, et al. Genetic determinants of serum testosterone concentrations in men. PLoS Genet. 2011; 7: e1002313.

119. Coviello AD, Haring R, Wellons M, Vaidya D, Lehtimaki T, Keildson S, et al. A Genome-Wide Association Meta-Analysis of Circulating Sex Hormone-Binding Globulin Reveals Multiple Loci Implicated in Sex Steroid Hormone Regulation. PLoS Genet. 2012; 8.

120. Ruth KS, Campbell PJ, Chew S, Lim EM, Hadlow N, Stuckey BG, et al. Genome-wide association study with 1000 genomes imputation identifies signals for nine sex hormone-related phenotypes. Eur J Hum Genet. 2016; 24: 284-90.

121. Eriksson AL, Perry JRB, Coviello AD, Delgado GE, Ferrucci L, Hoffman AR, et al. Genetic Determinants of Circulating Estrogen Levels and Evidence of a Causal Effect of Estradiol on Bone Density in Men. J Clin Endocrinol Metab. 2018; 103: 991-1004.

122. Ruth KS, Day FR, Tyrrell J, Thompson DJ, Wood AR, Mahajan A, et al. Using human genetics to understand the disease impacts of testosterone in men and women. Nat Med. 2020; 26: 252-8.

123. Dimou NL, Papadimitriou N, Gill D, Christakoudi S, Murphy N, Gunter MJ, et al. Sex hormone binding globulin and risk of breast cancer: a Mendelian randomization study. Int J Epidemiol. 2019.

124. Liu M, Jiang Y, Wedow R, Li Y, Brazel DM, Chen F, et al. Association studies of up to 1.2 million individuals yield new insights into the genetic etiology of tobacco and alcohol use. Nat Genet. 2019; 51: 237-44.

125. Xu Z, Xu H, Lu Y. Genetic Liability to Smoking and Breast Cancer Risk. Clin Epidemiol. 2020; 12: 1145-8.

126. Larsson SC, Carter P, Kar S, Vithayathil M, Mason AM, Michaëlsson K, et al. Smoking, alcohol consumption, and cancer: A mendelian randomisation study in UK Biobank and international genetic consortia participants. PLoS Med. 2020; 17: e1003178.

127. Zhu J, Jiang X, Niu Z. Alcohol consumption and risk of breast and ovarian cancer: A Mendelian randomization study. Cancer Genet. 2020; 245: 35-41.

128. Kranzler HR, Zhou H, Kember RL, Vickers Smith R, Justice AC, Damrauer S, et al. Genome-wide association study of alcohol consumption and use disorder in 274,424 individuals from multiple populations. Nat Commun. 2019; 10: 1499.

129. Bycroft C, Freeman C, Petkova D, Band G, Elliott LT, Sharp K, et al. The UK Biobank resource with deep phenotyping and genomic data. Nature. 2018; 562: 203-9.

130. Ong JS, Derks EM, Eriksson M, An J, Hwang LD, Easton DF, et al. Evaluating the role of alcohol consumption in breast and ovarian cancer susceptibility using population-based cohort studies and two-sample Mendelian randomization analyses. Int J Cancer. 2020.

131. Bergholdt HKM, Larsen MK, Varbo A, Nordestgaard BG, Ellervik C. Lactase persistence, milk intake, hip fracture and bone mineral density: a study of 97 811 Danish individuals and a meta-analysis. J Intern Med. 2018; 284: 254-69.

132. Vissers LET, Sluijs I, van der Schouw YT, Forouhi NG, Imamura F, Burgess S, et al. Dairy Product Intake and Risk of Type 2 Diabetes in EPIC-InterAct: A Mendelian Randomization Study. Diabetes Care. 2019; 42: 568-75.

133. Larsson SC, Mason AM, Kar S, Vithayathil M, Carter P, Baron JA, et al. Genetically proxied milk consumption and risk of colorectal, bladder, breast, and prostate cancer: a two-sample Mendelian randomization study. BMC Med. 2020; 18: 370.

134. Cornelis MC, Byrne EM, Esko T, Nalls MA, Ganna A, Paynter N, et al. Genome-wide meta-analysis identifies six novel loci associated with habitual coffee consumption. Mol Psychiatry. 2015; 20: 647-56.

135. Ong J-S, Law MH, An J, Han X, Gharahkhani P, Whiteman DC, et al. Association between coffee consumption and overall risk of being diagnosed with or dying from cancer among >300 000 UK Biobank participants in a large-scale Mendelian randomization study. Int J Epidemiol. 2019; 48: 1447-56.

136. Sillah A, Watson NF, Gozal D, Phipps AI. Obstructive sleep apnea severity and subsequent risk for cancer incidence. Preventive medicine reports. 2019; 15: 100886.

137. Cade BE, Chen H, Stilp AM, Gleason KJ, Sofer T, Ancoli-Israel S, et al. Genetic Associations with Obstructive Sleep Apnea Traits in Hispanic/Latino Americans. Am J Respir Crit Care Med. 2016; 194: 886-97.

138. Gao XL, Jia ZM, Zhao FF, An DD, Wang B, Cheng EJ, et al. Obstructive sleep apnea syndrome and causal relationship with female breast cancer: a mendelian randomization study. Aging (Albany N Y). 2020; 12.

139. Day F, Karaderi T, Jones MR, Meun C, He C, Drong A, et al. Large-scale genome-wide meta-analysis of polycystic ovary syndrome suggests shared genetic architecture for different diagnosis criteria. PLoS Genet. 2018; 14: e1007813.

140. Wu PF, Li RZ, Zhang W, Hu HY, Wang W, Lin Y. Polycystic ovary syndrome is causally associated with estrogen receptor-positive instead of estrogen receptor-negative breast cancer: a Mendelian randomization study. Am J Obstet Gynecol. 2020.

141. Wen Y, Wu X, Peng H, Li C, Jiang Y, Su Z, et al. Breast cancer risk in patients with polycystic ovary syndrome: a Mendelian randomization analysis. Breast Cancer Res Treat. 2020.

142. Ferreira MA, Vonk JM, Baurecht H, Marenholz I, Tian C, Hoffman JD, et al. Shared genetic origin of asthma, hay fever and eczema elucidates allergic disease biology. Nat Genet. 2017; 49: 1752-7.

143. Zhu Z, Lee PH, Chaffin MD, Chung W, Loh PR, Lu Q, et al. A genome-wide cross-trait analysis from UK Biobank highlights the shared genetic architecture of asthma and allergic diseases. Nat Genet. 2018; 50: 857-64.

144. Jiang X, Dimou NL, Zhu Z, Bonilla C, Lewis SJ, Lindstrom S, et al. Allergy, asthma, and the risk of breast and prostate cancer: a Mendelian randomization study. Cancer Causes Control. 2020; 31: 273-82.

145. Xue A, Wu Y, Zhu Z, Zhang F, Kemper KE, Zheng Z, et al. Genome-wide association analyses identify 143 risk variants and putative regulatory mechanisms for type 2 diabetes. Nat Commun. 2018; 9: 2941.

146. Escala-Garcia M, Morra A, Canisius S, Chang-Claude J, Kar S, Zheng W, et al. Breast cancer risk factors and their effects on survival: a Mendelian randomisation study. BMC Med. 2020; 18: 327.

147. Mahajan A, Taliun D, Thurner M, Robertson NR, Torres JM, Rayner NW, et al. Fine-mapping type 2 diabetes loci to single-variant resolution using high-density imputation and islet-specific epigenome maps. Nat Genet. 2018; 50: 1505-13.

148. Yuan S, Kar S, Carter P, Vithayathil M, Mason AM, Burgess S, et al. Is Type 2 Diabetes Mellitus Causally Associated with Cancer Risk? Evidence From a Two-Sample Mendelian Randomisation Study. Diabetes. 2020.

149. Kochi Y, Okada Y, Suzuki A, Ikari K, Terao C, Takahashi A, et al. A regulatory variant in CCR6 is associated with rheumatoid arthritis susceptibility. Nat Genet. 2010; 42: 515-9.

150. Freudenberg J, Lee HS, Han BG, Shin HD, Kang YM, Sung YK, et al. Genome-wide association study of rheumatoid arthritis in Koreans: population-specific loci as well as overlap with European susceptibility loci. Arthritis Rheum. 2011; 63: 884-93.

151. Hu HJ, Jin EH, Yim SH, Yang SY, Jung SH, Shin SH, et al. Common variants at the promoter region of the APOM confer a risk of rheumatoid arthritis. Exp Mol Med. 2011; 43: 613-21.

152. Terao C, Yamada R, Ohmura K, Takahashi M, Kawaguchi T, Kochi Y, et al. The human AIRE gene at chromosome 21q22 is a genetic determinant for the predisposition to rheumatoid arthritis in Japanese population. Hum Mol Genet. 2011; 20: 2680-5.

153. Myouzen K, Kochi Y, Okada Y, Terao C, Suzuki A, Ikari K, et al. Functional variants in NFKBIE and RTKN2 involved in activation of the NF-κB pathway are associated with rheumatoid arthritis in Japanese. PLoS Genet. 2012; 8: e1002949.

154. Okada Y, Terao C, Ikari K, Kochi Y, Ohmura K, Suzuki A, et al. Meta-analysis identifies nine new loci associated with rheumatoid arthritis in the Japanese population. Nat Genet. 2012; 44: 511-6.

155. Ahn C, Lee S, Park SK. Causal Inference between Rheumatoid Arthritis and Breast Cancer in East Asian and European Population: A Two-Sample Mendelian Randomization. Cancers (Basel). 2020; 12.

156. Jin Y, Birlea SA, Fain PR, Gowan K, Riccardi SL, Holland PJ, et al. Variant of TYR and autoimmunity susceptibility loci in generalized vitiligo. N Engl J Med. 2010; 362: 1686-97.

157. Jin Y, Andersen G, Yorgov D, Ferrara TM, Ben S, Brownson KM, et al. Genome-wide association studies of autoimmune vitiligo identify 23 new risk loci and highlight key pathways and regulatory variants. Nat Genet. 2016; 48: 1418-24.

158. Wen Y, Wu X, Peng H, Li C, Jiang Y, Liang H, et al. Cancer risks in patients with vitiligo: a Mendelian randomization study. J Cancer Res Clin Oncol. 2020.

159. Doherty A, Smith-Byrne K, Ferreira T, Holmes MV, Holmes C, Pulit SL, et al. GWAS identifies 14 loci for device-measured physical activity and sleep duration. Nat Commun. 2018; 9: 5257.

160. Papadimitriou N, Dimou N, Tsilidis KK, Banbury B, Martin RM, Lewis SJ, et al. Physical activity and risks of breast and colorectal cancer: a Mendelian randomisation analysis. Nat Commun. 2020; 11: 597.

161. Davies NM, Gaunt TR, Lewis SJ, Holly J, Donovan JL, Hamdy FC, et al. The effects of height and BMI on prostate cancer incidence and mortality: a Mendelian randomization study in 20,848 cases and 20,214 controls from the PRACTICAL consortium. Cancer Causes Control. 2015; 26: 1603-16.

162. Yengo L, Sidorenko J, Kemper KE, Zheng Z, Wood AR, Weedon MN, et al. Meta-analysis of genome-wide association studies for height and body mass index in approximately 700000 individuals of European ancestry. Hum Mol Genet. 2018; 27: 3641-9.

163. Kazmi N, Haycock P, Tsilidis K, Lynch BM, Truong T, Martin RM, et al. Appraising causal relationships of dietary, nutritional and physical-activity exposures with overall and aggressive prostate cancer: two-sample Mendelian-randomization study based on 79 148 prostate-cancer cases and 61 106 controls. Int J Epidemiol. 2019.

164. Cousminer DL, Stergiakouli E, Berry DJ, Ang W, Groen-Blokhuis MM, Korner A, et al. Genome-wide association study of sexual maturation in males and females highlights a role for body mass and menarche loci in male puberty. Hum Mol Genet. 2014; 23: 4452-64.

165. Bonilla C, Lewis SJ, Martin RM, Donovan JL, Hamdy FC, Neal DE, et al. Pubertal development and prostate cancer risk: Mendelian randomization study in a population-based cohort. BMC Med. 2016; 14.

166. Do R, Willer CJ, Schmidt EM, Sengupta S, Gao C, Peloso GM, et al. Common variants associated with plasma triglycerides and risk for coronary artery disease. Nat Genet. 2013; 45: 1345-52.

167. Isaacs A, Willems SM, Bos D, Dehghan A, Hofman A, Ikram MA, et al. Risk scores of common genetic variants for lipid levels influence atherosclerosis and incident coronary heart disease. Arterioscler Thromb Vasc Biol. 2013; 33: 2233-9.

168. Bull CJ, Bonilla C, Holly JMP, Perks CM, Davies N, Haycock P, et al. Blood lipids and prostate cancer: a Mendelian randomization analysis. Cancer medicine. 2016; 5: 1125-36.

169. Cornelis MC, Monda KL, Yu K, Paynter N, Azzato EM, Bennett SN, et al. Genome-Wide Meta-Analysis Identifies Regions on 7p21 (AHR) and 15q24 (CYP1A2) As Determinants of Habitual Caffeine Consumption. PLoS Genet. 2011; 7.

170. Amin N, Byrne E, Johnson J, Chenevix-Trench G, Walter S, Nolte IM, et al. Genome-wide association analysis of coffee drinking suggests association with CYP1A1/CYP1A2 and NRCAM. Mol Psychiatry. 2012; 17: 1116-29.

171. Taylor AE, Martin RM, Geybels MS, Stanford JL, Shui I, Eeles R, et al. Investigating the possible causal role of coffee consumption with prostate cancer risk and progression using Mendelian randomization analysis. Int J Cancer. 2017; 140: 322-8.

172. Xu J, Chang WS, Tsai CW, Bau DT, Xu Y, Davis JW, et al. Leukocyte telomere length is associated with aggressive prostate cancer in localized prostate cancer patients. EBioMedicine. 2020; 52: 102616.

173. Watts EL, Fensom GK, Smith Byrne K, Perez-Cornago A, Allen NE, Knuppel A, et al. Circulating insulin-like growth factor-I, total and free testosterone concentrations and prostate cancer risk in 200 000 men in UK Biobank. Int J Cancer. 2020.

174. He C, Qian Y, Liu B, Yang S, Ye D, Sun X, et al. Genetically Predicted Circulating Level of C-Reactive Protein Is Not Associated With Prostate Cancer Risk. Front Oncol. 2020; 10: 545603.

175. Kettunen J, Demirkan A, Wurtz P, Draisma HHM, Haller T, Rawal R, et al. Genome-wide study for circulating metabolites identifies 62 loci and reveals novel systemic effects of LPA. Nat Commun. 2016; 7.

176. Adams CD, Richmond R, Ferreira DLS, Spiller W, Tan V, Zheng J, et al. Circulating Metabolic Biomarkers of Screen-Detected Prostate Cancer in the ProtecT Study. Cancer Epidem Biomar. 2019; 28: 208-16.

177. Beynon RA, Richmond RC, Ferreira DLS, Ness AR, May M, Smith GD, et al. Investigating the effects of lycopene and green tea on the metabolome of men at risk of prostate cancer: The ProDiet randomised controlled trial. Int J Cancer. 2019; 144: 1918-28.

178. Gerstenblith MR, Shi JX, Landi MT. Genome-wide association studies of pigmentation and skin cancer: a review and meta-analysis. Pigment Cell & Melanoma Research. 2010; 23: 587-606.

179. Bonilla C, Gilbert R, Kemp JP, Timpson NJ, Evans DM, Donovan JL, et al. Using Genetic Proxies for Lifecourse Sun Exposure to Assess the Causal Relationship of Sun Exposure with Circulating Vitamin D and Prostate Cancer Risk. Cancer Epidem Biomar. 2013; 22: 597-606.

180. Sudlow C, Gallacher J, Allen N, Beral V, Burton P, Danesh J, et al. UK biobank: an open access resource for identifying the causes of a wide range of complex diseases of middle and old age. PLoS Med. 2015; 12: e1001779.

181. Mohammadi-Shemirani P, Chong M, Pigeyre M, Morton RW, Gerstein HC, Paré G. Effects of lifelong testosterone exposure on health and disease using Mendelian randomization. eLife. 2020; 9.

182. Cornelis MC, Fornage M, Foy M, Xun PC, Gladyshev VN, Morris S, et al. Genome-wide association study of selenium concentrations. Hum Mol Genet. 2015; 24: 1469-77.

183. Evans DM, Zhu G, Dy V, Heath AC, Madden PAF, Kemp JP, et al. Genome-wide association study identifies loci affecting blood copper, selenium and zinc. Hum Mol Genet. 2013; 22: 3998-4006.

184. Yarmolinsky J, Bonilla C, Haycock PC, Langdon RJQ, Lotta LA, Langenberg C, et al. Circulating Selenium and Prostate Cancer Risk: A Mendelian Randomization Analysis. Jnci-Journal of the National Cancer Institute. 2018; 110: 1035-8.

185. Haiman CA, Stram DO, Vickers AJ, Wilkens LR, Braun K, Valtonen-Andre C, et al. Levels of beta-microseminoprotein in blood and risk of prostate cancer in multiple populations. J Natl Cancer Inst. 2013; 105: 237-43.

186. Smith Byrne K, Appleby PN, Key TJ, Holmes MV, Fensom GK, Agudo A, et al. The role of plasma microseminoprotein-beta in prostate cancer: an observational nested case-control and Mendelian randomization study in the European prospective investigation into cancer and nutrition. Ann Oncol. 2019.

187. O'Seaghdha CM, Wu H, Yang Q, Kapur K, Guessous I, Zuber AM, et al. Meta-analysis of genome-wide association studies identifies six new Loci for serum calcium concentrations. PLoS Genet. 2013; 9: e1003796.

188. Yarmolinsky J, Berryman K, Langdon R, Bonilla C, Davey Smith G, Martin RM, et al. Mendelian randomization does not support serum calcium in prostate cancer risk. Cancer Causes Control. 2018; 29: 1073-80.

189. Tan VY, Biernacka KM, Dudding T, Bonilla C, Gilbert R, Kaplan RC, et al. Reassessing the Association between Circulating Vitamin D and IGFBP-3: Observational and Mendelian Randomization Estimates from Independent Sources. Cancer Epidem Biomar. 2018; 27: 1462-71.

190. Hazra A, Kraft P, Selhub J, Giovannucci EL, Thomas G, Hoover RN, et al. Common variants of FUT2 are associated with plasma vitamin B12 levels. Nat Genet. 2008; 40: 1160-2.

191. Hazra A, Kraft P, Lazarus R, Chen C, Chanock SJ, Jacques P, et al. Genome-wide significant predictors of metabolites in the one-carbon metabolism pathway. Hum Mol Genet. 2009; 18: 4677-87.

192. Collin SM, Metcalfe C, Palmer TM, Refsum H, Lewis SJ, Smith GD, et al. The causal roles of vitamin B(12) and transcobalamin in prostate cancer: can Mendelian randomization analysis provide definitive answers? Int J Mol Epidemiol Genet. 2011; 2: 316-27.

193. Gueant JL, Chabi NW, Gueant-Rodriguez RM, Mutchinick OM, Debard R, Payet C, et al. Environmental influence on the worldwide prevalence of a 776C->G variant in the transcobalamin gene (TCN2). J Med Genet. 2007; 44: 363-7.

194. Khankari NK, Murff HJ, Zeng C, Wen W, Eeles RA, Easton DF, et al. Polyunsaturated fatty acids and prostate cancer risk: a Mendelian randomisation analysis from the PRACTICAL consortium. Br J Cancer. 2016; 115: 624-31.

195. Lemaitre RN, Tanaka T, Tang WH, Manichaikul A, Foy M, Kabagambe EK, et al. Genetic Loci Associated with Plasma Phospholipid n-3 Fatty Acids: A Meta-Analysis of Genome-Wide Association Studies from the CHARGE Consortium. PLoS Genet. 2011; 7.

196. Wray NR, Ripke S, Mattheisen M, Trzaskowski M, Byrne EM, Abdellaoui A, et al. Genome-wide association analyses identify 44 risk variants and refine the genetic architecture of major depression. Nat Genet. 2018; 50: 668-81.

197. Chen X, Kong J, Diao X, Cai J, Zheng J, Xie W, et al. Depression and prostate cancer risk: A Mendelian randomization study. Cancer medicine. 2020; 9: 9160-7.

198. Klimentidis YC, Raichlen DA, Bea J, Garcia DO, Wineinger NE, Mandarino LJ, et al. Genome-wide association study of habitual physical activity in over 377,000 UK Biobank participants identifies multiple variants including CADM2 and APOE. Int J Obes (Lond). 2018; 42: 1161-76.

199. Shungin D, Winkler TW, Croteau-Chonka DC, Ferreira T, Locke AE, Magi R, et al. New genetic loci link adipose and insulin biology to body fat distribution. Nature. 2015; 518: 187-96.
